# TiF_4_-mediated, one-pot, reductive amination of carboxylic acids with borane–ammonia†

**DOI:** 10.1039/d4ra05888g

**Published:** 2024-09-30

**Authors:** Madison J. Snyder, Abdulkhaliq A. Alawaed, Chunge Li, Samantha Pacentine, Henry J. Hamann, P. Veeraraghavan Ramachandran

**Affiliations:** a Herbert C. Brown Center for Borane Research, Department of Chemistry, Purdue University West Lafayette Indiana 47907 USA chandran@purdue.edu

## Abstract

A facile one-pot, two-step, reductive alkylation of amines with carboxylic acids has been achieved with BH_3_–NH_3_ as an air- and moisture-stable reductant in the presence of TiF_4_. The catalyst is effective for both amidation and reduction steps, and the product amines are isolated in high yields as either the free amines, for those products containing an arylamine, or the borane-complexes. The free amine can be separated from these complexes using BF_3_–Et_2_O, followed by hydrolysis. The amide reduction has been demonstrated for primary, secondary, and tertiary amides, as well as lactams, and the reductive amination is applicable to a wide variety of aromatic and aliphatic acids as well as amines.

## Abstract 2

Reductive alkylation of amines with carboxylic acids is achieved using BH_3_–NH_3_ and TiF_4_, yielding free amines or their borane-complexes.

## Graphical abstract

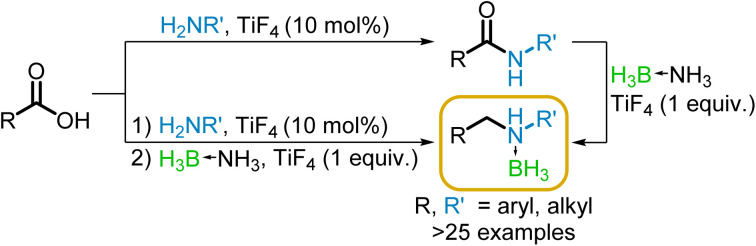

## Introduction

Amines constitute an integral part of natural and synthetic molecules, with wide-ranging applications, particularly in agrochemical and pharmaceutical industry.^1^ Nucleophilic substitutions and transition-metal catalyzed aminations are common procedures to prepare alkyl or aryl amines.^2–5^ Reduction of nitriles,^6,7^ amides,^8,9^ or imines, and reductive amination of carbonyls (or reductive alkylation of amines) are also well-established protocols to access amines. Borohydrides and borane–Lewis bases, particularly borane–amines, are well studied for both classes of reactions.^10–12^

While the reductive amination of aldehydes and ketones has been well studied,^13–16^ a similar reaction of carboxylic acids is undergoing a renewed interest as a field of research. Recent reports on the reductive amination of carboxylic acids involve conversion of acids to amides, followed by reduction with silanes^17–19^ or selective reduction of acids to aldehydes or silyl acetals,^20–25^ followed by reductive amination. The silane reduction protocols suffer from several drawbacks including stoichiometric waste, moisture sensitivity, reagent cost, and the cumbersome separation of the amine from the siloxane byproduct. Hydrogenation of a mixture of acid and amine in the presence of homogeneous and heterogeneous catalysts has been studied with a variety of metals and catalysts.^26–33^ Intriguingly, there are no reported practical procedures for the reductive amination of carboxylic acids utilizing versatile borane–amines.

**Scheme 1:**
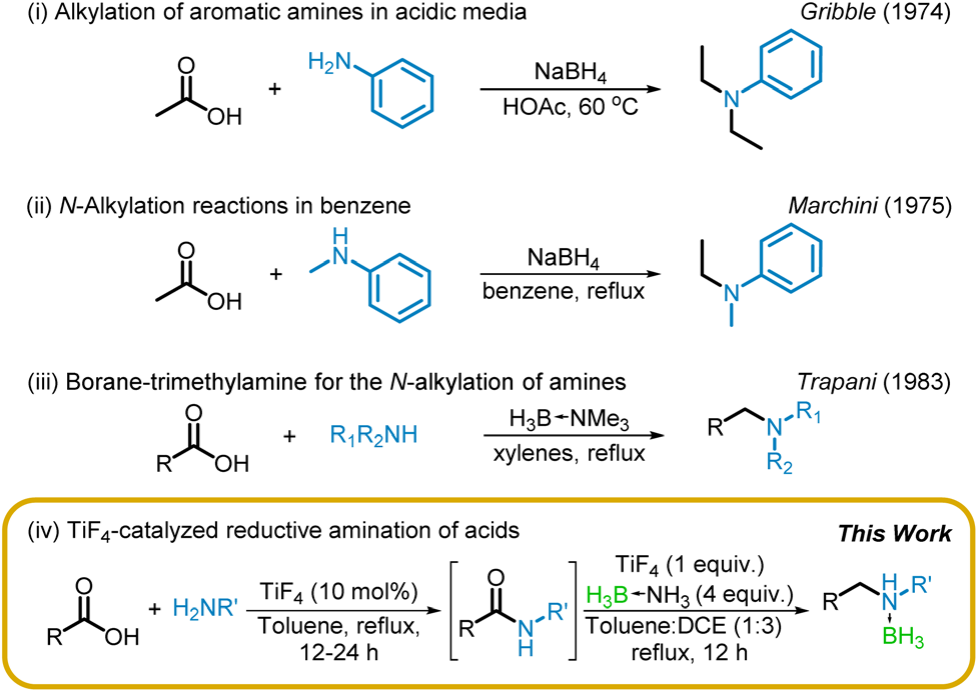
Reductive amination of acids *via* boranes.

The first report of a reductive alkylation of amines with acids as the electrophile was described, independently, by Gribble^34^ and Marchini^35^ and their co-workers five decades ago (Scheme 1(i) and (ii)). They described the use of sodium borohydride as the reductant in carboxylic acid or benzene solvent, although the reaction, presumably, proceeded *via* the acyloxyborohydride. However, these protocols are not practical and, accordingly, have not received much attention. A similar reductive alkylation *via* the intermediacy of amides, in varying yields, with borane–trimethylamine was described, nearly a decade later, by Trapani^36^ and co-workers (Scheme 1(iii)). Given the popularity of borane–amines for reductive amination,^37–39^ it is surprising that this work has not received much attention.

Our interest in the chemistry of borane–amines, particularly the reactions of borane–ammonia catalyzed by titanium tetrachloride^40,41^ has led to the successful reduction of carboxamides.^42^ We have also recently reported that TiF_4_ is an efficient catalyst for the direct amidation of acids.^43^ This prompted us to examine (i) the efficacy of TiF_4_ for the reduction of amides, so that (ii) a one-pot reductive amination of acids can be developed. Our success in both fronts resulting in a TiF_4_-mediated reductive amination of acids is detailed below (Scheme 1(iv)).

## Results and discussion

Our initial focus was to achieve the reduction of amides using borane–ammonia (BH_3_NH_3_). Other borane–amines were not examined for the reduction due to the menace of amine byproduct that will be difficult to separate. Prior TiCl_4_ catalyzed^42,44^ and tris(pentafluorophenyl)borane [(C_6_F_5_)_3_B] catalyzed^45^ amide reductions using BH_3_NH_3_ gave further motivation for the project.

With *N*-benzylbenzamide (1a) as the representative amide, we began optimizing the reduction. The results are summarized in Table 1. Following our reported TiCl_4_ amide reduction using two equiv. of borane–ammonia and 20 mol% of the catalyst, the reaction was carried out using TiF_4_ in refluxing DCE for 24 h. Workup revealed, by ^1^H NMR spectroscopy, a mixture of 36% of *N*,*N*-dibenzylamine (2a) and 32% of a second product identified as the 2a–BH_3_ complex,^46^ along with 32% of the unreacted amide (Table 1, entry 1). Given the low conversion, the catalyst was increased to 100 mol%, which resulted in a 44% conversion to the amine–borane as the sole product (entry 2). It is noteworthy that the TiCl_4_ catalyzed reduction yields the amine or the amine–hydrochloride product in some cases,^42,44^ the halide being delivered from the catalyst. Noting that earlier reported^45^ borane reductions of amides have used either 3 or 4 equiv. of the reducing agent, the reaction was repeated with 3 and 4 equivalents of BH_3_NH_3_, resulting in a respective increase in yield of the borane–amine to 90% and 97% in 16 h (entries 3 and 4). Decreasing the reaction time to 12 h had no deleterious effect on the yield (96%, entry 5). Having obtained nearly quantitative transformation of the amide to the borane–amine, an attempt was made to decrease TiF_4_ load to 50 mol% when the yield decreased to 75% (entry 6).

**Table 1:** Optimization of TiF_4_-mediated reduction of *N*-benzylbenzamide with BH_3_NH_3_

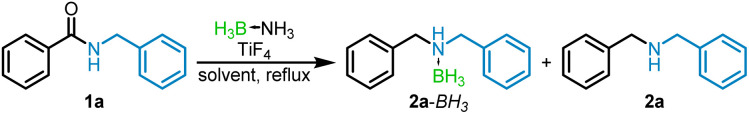					
Entry	BH_3_NH_3_ (equiv.)	TiF_4_ (equiv.)	Solvent	Time (h)	Conversion^a^2a–BH_3_ : 2a : 1a
1	2	0.2	DCE	24	32 : 36 : 32
2	2	1	DCE	24	44 : 0 : 56
3	3	1	DCE	24	(90)^b^
**4**	**4**	**1**	**DCE**	**16**	(**97**)^b^
5	4	1	DCE	12	(96)^b^
6	4	0.5	DCE	24	(75)^b^
7	2	0.2	Toluene	24	4 : 32 : 64^c^
8	2.5	1	Toluene	24	13 : 59 : 28^c^
9	3	1	Toluene	24	4 : 71 : 25^c^
10	4	1	Xylenes	12	(67)^d^
11	2 (HBpin)	0.1	Toluene	24	0 : 71 : 29
12	3 (HBpin)	0.1	Toluene	24	0 : 100 : 0 (88)^d^

a Ratio determined by ^1^H NMR spectroscopy.

b Isolated yield of 2a–BH_3_.

c Combined value of benzyl alcohol and 1a.

d Isolated yield of 2a.

**Scheme 2:**
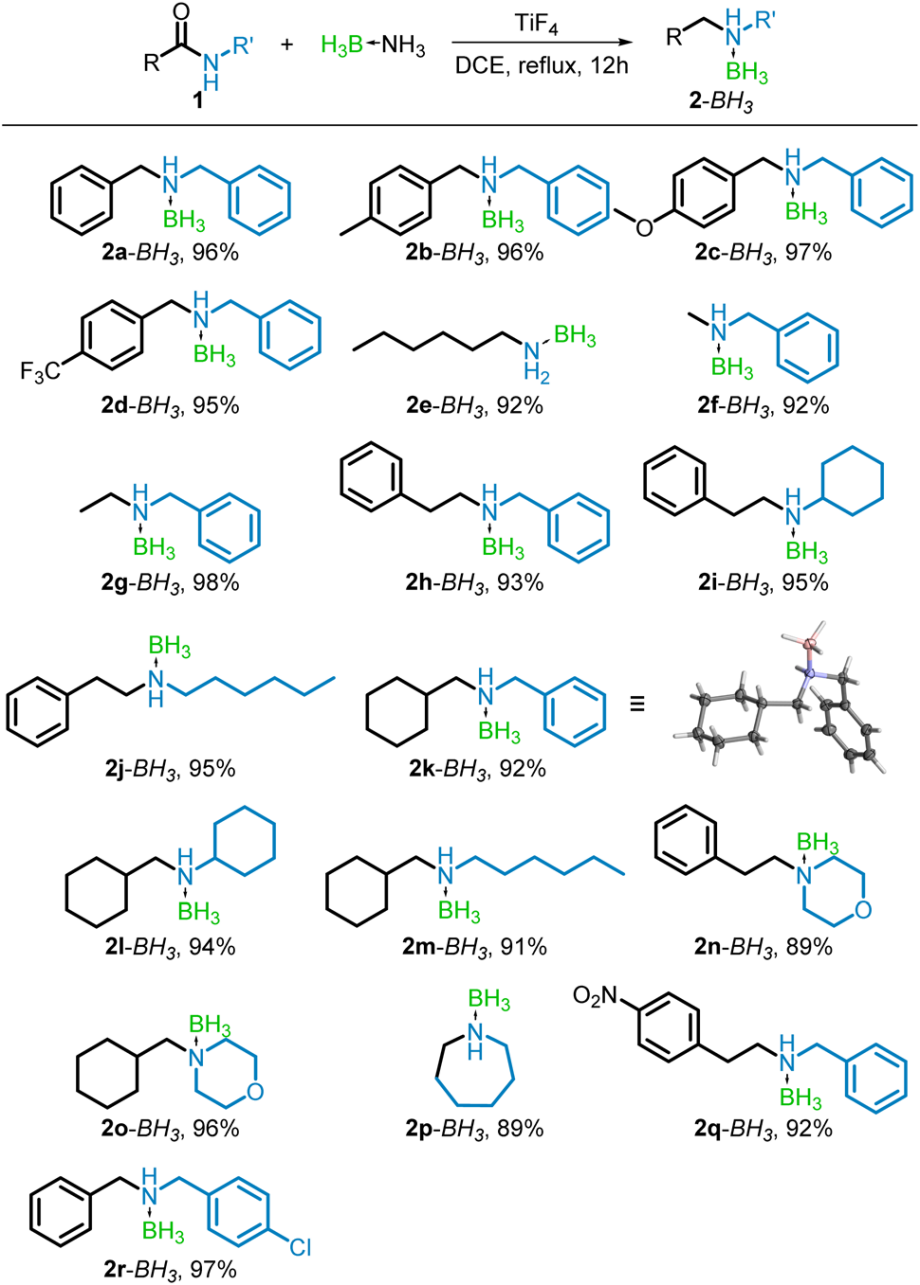
TiF_4_-catalyzed reduction of amides with BH_3_NH_3_. Isolated yields shown.

Since the TiF_4_-mediated amidation is more efficient in toluene as the solvent,^43^ the reduction was now optimized in toluene, to develop a one-pot reductive amination of acids. Although it was noticed that the use of toluene greatly encouraged conversion to the free amine (32%) over the borane–amine (4%), very low combined yields were obtained (entry 7). Increasing the catalyst or reductant, or switching the solvent to xylenes did not improve the results considerably (entries 8–10). In addition, competing reduction to the alcohol was also observed! As can be seen from entries 11 and 12, satisfactory conversions to 2a were achieved using pinacolborane (HBpin) as an alternate reductant, however, isolation of the product was hindered by the presence of byproduct pinacol. Thus, a reaction in refluxing DCE with four equiv. of BH_3_NH_3_ and 100 mol% TiF_4_ was chosen as the optimal conditions for subsequent studies. The scope of the TiF_4_-catalyzed BH_3_NH_3_ reduction of amides was examined for a variety of amides derived from both aromatic and aliphatic acids and amines. The borane–amines synthesized using this reduction protocol and their respective yields are summarized in Scheme 2. For the reduction of amides derived from aromatic acids, electron donating groups in the *para* position (–CH_3_ and –OMe) of the aryl moiety were well tolerated, producing excellent yields of 2b–BH_3_ and 2c–BH_3_ (96% and 97% respectively). The electron withdrawing –CF_3_ group performed similarly (95% yield of 2d–BH_3_) showing tolerance for electronic substitution. Amides derived from aliphatic acids (1e–1q) were also reduced in excellent yields (89–98%). X-ray crystallographic analysis of 2k–BH_3_, crystallized from hexane, served to confirm the formation of the borane–amine (Scheme 2, 2k–BH_3_). As a representative primary amine, *N*-hexanamide (1e) was reduced, providing a 92% yield of the corresponding borane–amine (2e–BH_3_). *N*-Benzylformamide (1f) and *N*-phenylacetamide (1g) were reduced in 92% and 98% yields respectively. The method also proved effective for the reduction of a cyclic amide, caprolactam (1p), to borane–azepane (2p–BH_3_) in 89% yield.

**Scheme 3:**
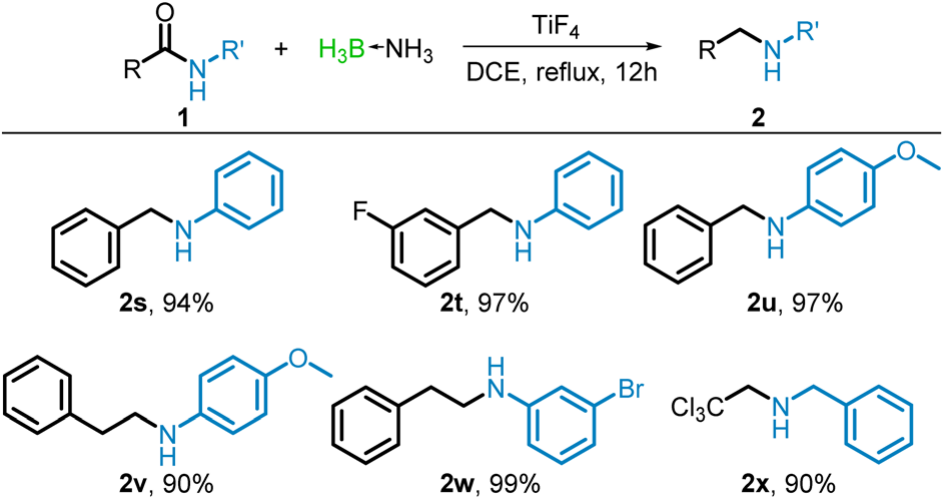
TiF_4_-catalyzed reduction of anilides with BH_3_NH_3_. Isolated yields shown.

Notably, the reduction of amides (anilides) derived from aromatic amines (1s–1x) resulted in none of the borane–amine, and the free amines (2s–2x) were isolated in 90–99% yields. This can be rationalized by the weak nucleophilicity of anilines resulting in poor coordination with borane. Thus, *N*-phenylbenzamide (1s) was reduced to *N*-benzylaniline (2s) in 94% yield. When the acyl moiety of the amide contained an electron withdrawing fluoride in the *meta* position (1t) the reduction resulted in excellent yields (97%) of the *N*-benzylaniline (2t). As with the acyl moiety, the aniline moiety tolerated both electron withdrawing and donating groups. Both *N*-(4-methoxyphenyl)benzamide (1u) and *N*-(4-methoxyphenyl)-2-phenylacetamide (1v) were reduced to their corresponding amines (2u and 2v) in excellent yields (97% and 90%) and 3-bromo-*N*-phenethylaniline (1w) yielded 99% of 2w. Curiously, the reduction of *N*-benzyl-2,2,2-trichloroacetamide (1x) also resulted in producing the corresponding free amine (2x), probably due to the strong inductive effect of the –CCl_3_ group weakening the borane coordination (Scheme 3).

**Scheme 4:**
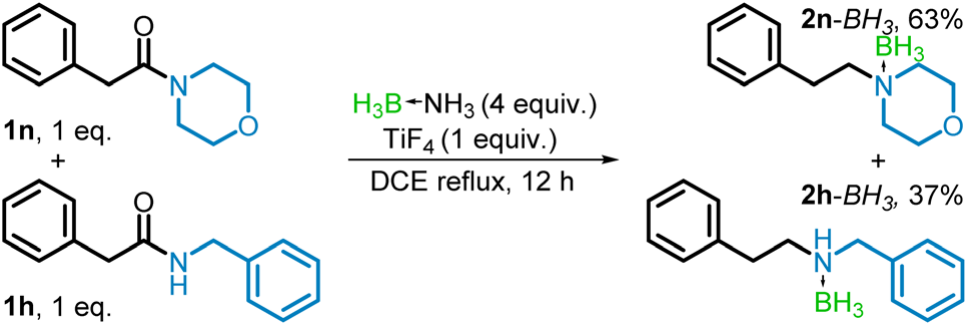
Competitive reduction of 2° *vs.* 3° amide.

The amide reduction methodology was then applied to a competitive reaction between a secondary (1h) and tertiary amide (1n). Using the standard condition (4 equiv. BH_3_NH_3_ and 1 equiv. TiF_4_ in refluxing DCE), the reaction mixture was analyzed using ^1^H NMR after 12 h. The expected borane–amine products 2h–BH_3_ and 2n–BH_3_ were present in a ratio 37% to 63% respectively, along with the corresponding quantities of unreacted amide. These results indicate a nearly 2 to 1 preference for the reduction of the tertiary *vs.* the secondary amide (Scheme 4).

After achieving the TiF_4_-mediated BH_3_NH_3_ reduction of a variety of amides to the corresponding amines or borane–amines in good to excellent yields, optimization of a one-pot tandem reductive amination procedure was performed. First, the amidation was carried out with the previously reported conditions,^43^ in refluxing toluene for 12 h for aliphatic and 24 h for aromatic acids. The challenge was overcoming the change of solvent from toluene to DCE for the reduction. As discussed above, the reduction in toluene is not satisfactory, but removing toluene to substitute DCE will make the process tedious. Accordingly, the use of mixed solvent system for the reduction step was envisioned. After completing the amidation in toluene in the presence of 10 mol% TiF_4_, three reduction experiments were performed by adding DCE so that the overall ratios of toluene and DCE were 1 : 3, 1 : 1, and 3 : 1. An equiv. of TiF_4_ and 4 equiv. of BH_3_NH_3_ were also added and the reductions completed. Among these, the 1 : 3 ratio of toluene : DCE was found to provide the best yields of dibenzylamine–borane (89%) with the least amount of the benzyl alcohol side-product (*vide supra*: optimization). This solvent mixture was used for all subsequent one-pot reductive aminations. An attempt to carry out the amidation of benzoic acid with benzylamine in this solvent mixture was not satisfactory.

**Scheme 5:**
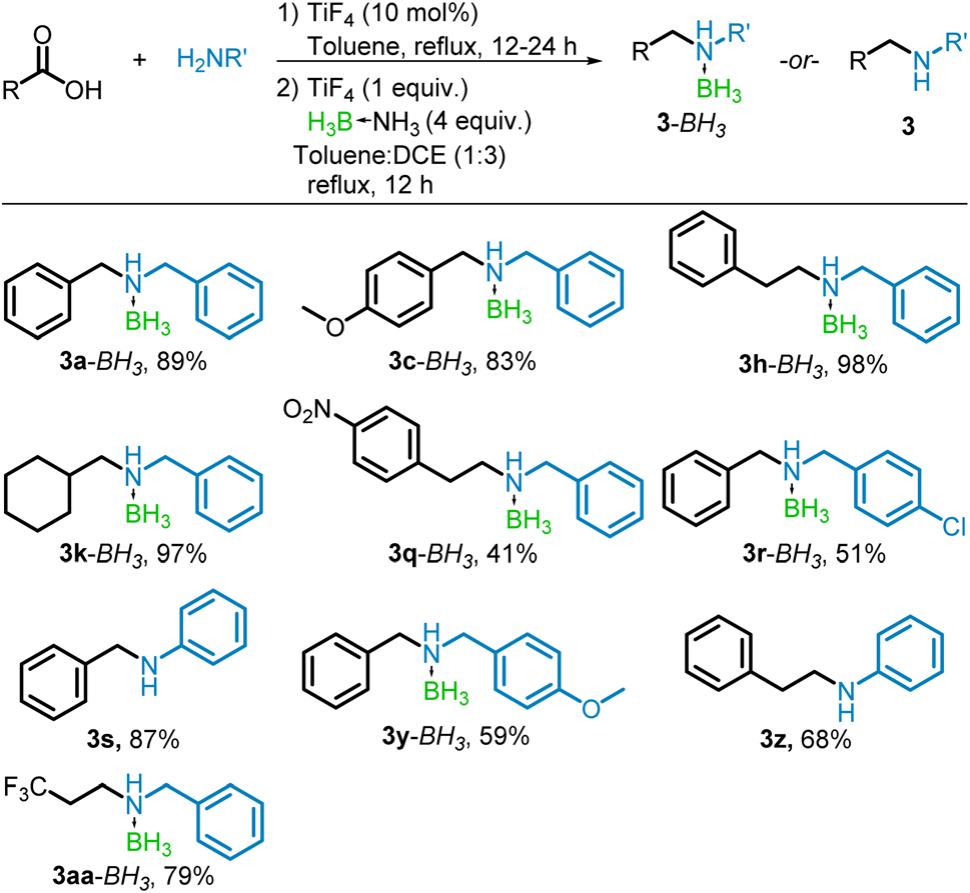
One pot TiF_4_-catalyzed reductive amination of acids with BH_3_NH_3_. Isolated yields shown.

The tandem conversion of the carboxylic acid to the borane–amine proved in many cases to be equally effective as the stepwise process, resulting in good to excellent yields of the borane–amine or free-amine products. The reaction of benzoic, phenylacetic, cyclohexanecarboxylic, and trifluoropropanoic acids with benzylamine yielded the borane–amines (3a–BH_3_, 3h–BH_3_, 3k–BH_3_, and 3aa–BH_3_) without difficulty (79–98%). In cases where trace amounts of alcohol formed, it was simply washed away with hexane, leaving pure borane–amine. Decorating the carboxylic acid with an electron donating group (–OMe) in the *para* position, resulted in a near quantitative conversion to the borane–amine 3c–BH_3_ (98%). However, 4-nitrophenylacetic acid had moderate conversion to 3q–BH_3_ after column chromatography (41%) due to the reduction to the alcohol in place of the amine. Similarly, the use of both 4-chlorobenzylamine and 4-methoxybenzylamine resulted in significant formation of the alcohol product. After separation by column chromatography, they yielded their respective borane–amines (3r–BH_3_ and 3y–BH_3_) in 51% and 59%. The conversion to the free amine by use of aniline proceeded in good yields when paired with benzoic and phenylacetic acids (87 and 68% respectively for 3s and 3z) with the lower yield of *N*-phenylethylaniline (3z) due to alcohol formation. These results are summarized in Scheme 5.

**Scheme 6:**
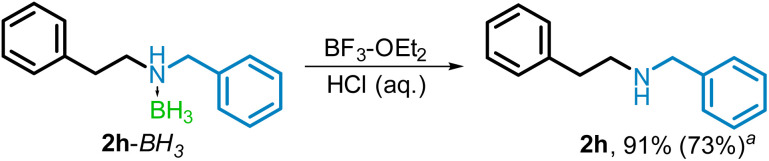
Conversion of borane–amine to free amine. ^*a*^Yield of 2h when amidation, reduction and removal of BH_3_ are carried out sequentially in a one-pot reaction.

While the reaction products containing an aromatic amine (2s–2w, 3s, 3z) or a strongly withdrawing group at the alpha position (2x) were obtained as the free amine, the majority of the products of the amide reduction and reductive amination were isolated as the corresponding complexes with borane (BH_3_). The simple conversion of BH_3_ complex to free amine was demonstrated by reacting product 2h–BH_3_ with BF_3_–OEt_2_, where, after acidic aqueous workup, the free amine (2h) was obtained in 91% isolated yield (Scheme 6). A one-pot reaction to prepare 2h, without isolation of 2h–BH_3_ was also examined. Starting from phenylacetic acid and benzylamine, the amidation, reduction, and removal of BH_3_ using BF_3_–OEt_2_ were carried out sequentially, resulting in an 73% isolated yield of 2h.

**Scheme 7:**
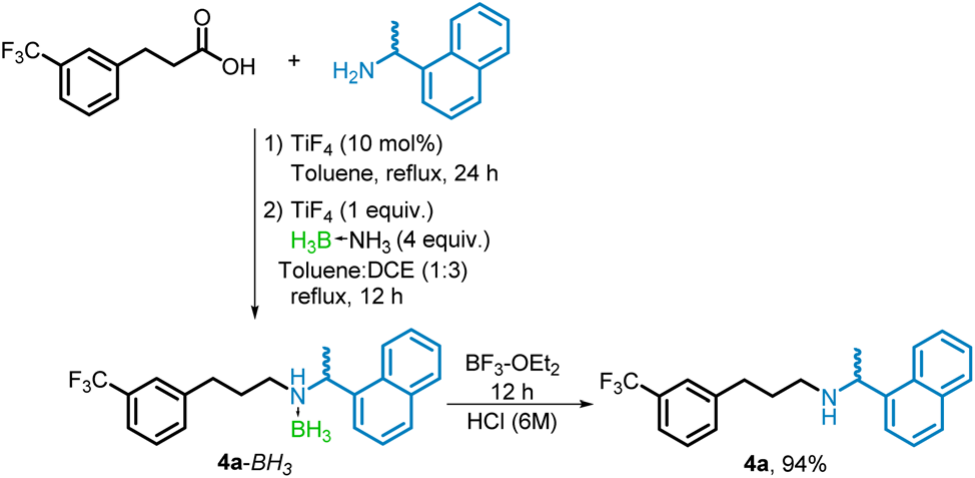
Synthesis of racemic cinacalcet (4a).

The tandem conversion of the carboxylic acid to the borane–amine followed by the process for the decomplexation of BH_3_ using BF_3_–OEt_2_ was additionally applied to the synthesis of racemic cinacalcet. The chiral (*R*)-cinacalcet is used as a calcimimetic agent. In our procedure 3-(3-(trifluoromethyl)phenyl)propanoic acid and (±)1-(1-naphthyl)ethylamine were subjected to the tandem amidation/reduction conditions to provide the corresponding borane–amine 4a–BH_3_. Curiously, the NMR characterization of this adduct showed the presence of 2 components. This is thought to be caused by a sort of isomerism arising from the adjacent chiral center and borane-coordinated amine, as the two components were separable by column chromatography. The decomplexation protocol using BF_3_–OEt_2_ was applied to this two component mixture whereupon the peaks recoalesced to a single species which was identified as racemic cinacalcet (4a). The product 4a was isolated in 94% yield, which is an improvement over similar protocols.^25^ The decomplexation of 4a–BH_3_ to 4a did require a somewhat longer reaction time (12 h) and the use a stronger acid (6 M) in the workup procedure compared with the conversion of 2h–BH_3_ to 2h (Scheme 7).

## Conclusions

In conclusion, we have developed open-air processes for the reduction of amides and a one-pot, reductive amination of carboxylic acids to the corresponding amines or borane–amines using borane–ammonia with titanium tetrafluoride as the activator in refluxing dichloroethane. This new synthesis tolerates amides derived from aromatic and aliphatic acids and amines as well as some functional group tolerability towards nitro, halogens, and electron donating groups. This process can also be viewed as a new process for the synthesis of borane–amines from carboxylic acids, though the amines can be readily freed from their borane complexes using boron trifluoride diethyl etherate, a process which was applied to the synthesis of racemic cinacalcet.

## Data availability

The data supporting this article have been included as part of the ESI.†

## Conflicts of interest

There are no conflicts to declare.

## Supplementary Material

RA-014-D4RA05888G-s001

RA-014-D4RA05888G-s002

## References

[cit1] Afanasyev O. I., Kuchuk E., Usanov D. L., Chusov D. (2019). Chem. Rev..

[cit2] Yang Q., Wang Q. F., Yu Z. K. (2015). Chem. Soc. Rev..

[cit3] Park Y., Kim Y., Chang S. (2017). Chem. Rev..

[cit4] Trowbridge A., Walton S. M., Gaunt M. J. (2020). Chem. Rev..

[cit5] Kuai M. Y., Jia Z. H., Chen L. J., Gao S., Fang W. W. (2024). Eur. J. Org Chem..

[cit6] Pandey V. K., Tiwari C. S., Rit A. (2021). Org. Lett..

[cit7] Kumar R., Meher R. K., Sharma J., Sau A., Panda T. K. (2023). Org. Lett..

[cit8] Ye P. Q., Shao Y. L., Ye X. Z., Zhang F. J., Li R. H., Sun J. N., Xu B. H., Chen J. X. (2020). Org. Lett..

[cit9] Vinayagam V., Sadhukhan S. K., Karre S. K., Srinath R., Maroju R. K., Karra P. R., Bathula H., Kundrapu S., Surukonti S. R. (2023). Org. Lett..

[cit10] Matos K. and Burkhardt E. R., in Pharmaceutical Process Chemistry, Wiley-VCH Verlag GmbH & Co. KGaA, 2011, pp. 127–143

[cit11] Baxter E. W. and Reitz A. B., in Organic Reactions, 2002, pp. 1–714

[cit12] Abdel-Magid A. F., Mehrman S. J. (2006). Org. Process Res. Dev..

[cit13] Podyacheva E., Afanasyev O. I., Tsygankov A. A., Makarova M., Chusov D. (2019). Synthesis.

[cit14] Reshi N. U. D., Saptal V. B., Beller M., Bera J. K. (2021). ACS Catal..

[cit15] Irrgang T., Kempe R. (2020). Chem. Rev..

[cit16] Bhattacharyya S., Chatterjee A., Williamson J. S. (1995). Synlett.

[cit17] Stoll E. L., Tongue T., Andrews K. G., Valette D., Hirst D. J., Denton R. M. (2020). Chem. Sci..

[cit18] Andrews K. G., Summers D. M., Donnelly L. J., Denton R. M. (2016). Chem. Commun..

[cit19] Fu M.-C., Shang R., Cheng W.-M., Fu Y. (2015). Angew. Chem., Int. Ed..

[cit20] Ouyang L., Miao R., Yang Z. H., Luo R. S. (2023). J. Catal..

[cit21] Minakawa M., Okubo M., Kawatsura M. (2016). Tetrahedron Lett..

[cit22] Andrews K. G., Faizova R., Denton R. M. (2017). Nat. Commun..

[cit23] Zhu L., Wang L. S., Li B. J., Li W., Fu B. Q. (2016). Catal. Sci. Technol..

[cit24] Nguyen T. V. Q., Yoo W. J., Kobayashi S. (2016). Adv. Synth. Catal..

[cit25] Sorribes I., Junge K., Beller M. (2014). J. Am. Chem. Soc..

[cit26] Coeck R., Meeprasert J., Li G. N., Altantzis T., Bals S., Pidko E. A., De Vos D. E. (2021). ACS Catal..

[cit27] Emayavaramban B., Chakraborty P., Sundararaju B. (2019). ChemSusChem.

[cit28] Liu W. P., Sahoo B., Spannenberg A., Junge K., Beller M. (2018). Angew. Chem., Int. Ed..

[cit29] Toyao T., Siddiki S., Morita Y., Kamachi T., Touchy A. S., Onodera W., Kon K., Furukawa S., Ariga H., Asakura K., Yoshizawa K., Shimizu K. (2017). Chem.–Eur. J..

[cit30] Shi Y. P., Kamer P. C. J., Cole-Hamilton D. J. (2017). Green Chem..

[cit31] Qiao C., Liu X. F., Liu X., He L. N. (2017). Org. Lett..

[cit32] Sorribes I., Cabrero-Antonino J. R., Vicent C., Junge K., Beller M. (2015). J. Am. Chem. Soc..

[cit33] Nuñez A. A., Eastham G. R., Cole-Hamilton D. J. (2007). Chem. Commun..

[cit34] Gribble G. W., Lord P. D., Skotnicki J., Dietz S. E., Eaton J. T., Johnson J. L. (1974). J. Am. Chem. Soc..

[cit35] Marchini P., Liso G., Reho A., Liberatore F., Moracci F. M. (1975). J. Org. Chem..

[cit36] Trapani G., Reho A., Latrofa A. (1983). Synthesis.

[cit37] Zhou Q. W., Meng W., Yang J., Du H. F. (2018). Angew. Chem., Int. Ed..

[cit38] Liao W. Y., Chen Y. F., Liu Y. X., Duan H. F., Petersen J. L., Shi X. D. (2009). Chem. Commun..

[cit39] Sato S., Sakamoto T., Miyazawa E., Kikugawa Y. (2004). Tetrahedron.

[cit40] Ramachandran P. V., Alawaed A. A., Hamann H. J. (2023). Org. Lett..

[cit41] Ramachandran P. V., Alawaed A. A., Hamann H. J. (2023). Org. Lett..

[cit42] Ramachandran P. V., Alawaed A. A., Singh A. (2023). Molecules.

[cit43] Alawaed A. A., Ramachandran P. V. (2024). Org. Biomol. Chem..

[cit44] Zang Y., Sui Q., Xu Q., Ma M., Li G., Zhu F. (2023). Tetrahedron Lett..

[cit45] Pan Y., Luo Z., Han J., Xu X., Chen C., Zhao H., Xu L., Fan Q., Xiao J. (2019). Adv. Synth. Catal..

[cit46] Ramachandran P. V., Hamann H. J., Lin R. (2021). Dalton Trans..

