# Supplementary material for: TiF4-mediated, one-pot, reductive amination of carboxylic acids with borane–ammonia
Source: RSC Adv. 2024 Sep 30;14(42):31205–9. doi: 10.1039/d4ra05888g (PMC11441464; doi:10.1039/d4ra05888g)
Supplement: RA-014-D4RA05888G-s001 [file RA-014-D4RA05888G-s001.pdf]

Supplementary Information (SI) for RSC Advances.
This journal is © The Royal Society of Chemistry 2024

SUPPORTING INFORMATION:

TiF_4-mediated, one-pot, reductive amination of carboxylic acids with borane-ammonia

Madison J. Snyder, Abdulkhaliq A. Alawaed, Chunge Li, Samantha Pacentine, Henry J. Hamann, and P. Veeraraghavan Ramachandran*

Herbert C. Brown Center for Borane Research, Department of Chemistry, Purdue University, West Lafayette, Indiana 47907, United States

*E-mail: chandran@purdue.edu

Contents:

General Information: S2

General procedure (1) for the preparation of amines and borane-amines from amides: S2

General procedure (2) for the preparation of amines and borane-amines from carboxylic acids: S2-S3

Procedure (3) for the competitive reduction of tertiary and secondary amides: S3

Procedure (4) for the recovery of the amine from the borane-amine: S3

Procedure (5) for the one-pot preparation of N-benzyl-2-phenylethan-1-amine (**2h**): S3-S4

Procedure for the crystallization of borane-*N*-benzyl-1-cyclohexylmethanamine (**2k**-BH_3): S4

Characterization of amine and borane-amine products: S5-S13

References: S14

NMR spectra of amine and borane-amine products: S15-S60

General Information:

The borane–ammonia was prepared according to our earlier published procedures.[1] The carboxamides used in this project were prepared according to a previously published procedure using TiF_4.[2] The other reagents, TiF4, sodium borohydride, solvents, carboxylic acids, as well as the amides used to prepare the carboxamides, were purchased from Sigma-Aldrich, Oakwood Chemical, or Fisher Scientific and used as received. Thin-layer chromatography (TLC) was performed on silica gel F60 plates and visualized under UV light. The structures of the product amines and borane-amines were confirmed with nuclear magnetic resonance (NMR) spectroscopy and measured in δ values in parts per million (ppm). The ^{1}H, ^{13}C, ^{11}B, and ^{19}F NMR spectra of the reduction products were recorded on a Bruker 300 MHz spectrometer at ambient temperature. ^{1}H NMR spectra of reduction products were recorded from a Bruker 300 MHz spectrometer at ambient temperature and calibrated against the residual solvent peak of $CDCl_3$ (δ = 7.26 ppm) as an internal standard. The ^{13}C NMR spectra were recorded at 75 MHz (297 K) and calibrated using $CDCl_3$ (δ = 77.0 ppm) as an internal standard. The $_{19}F$ NMR spectra were recorded at 282 MHz and calibrated using $CFCl_3$ (δ = 0 ppm) as the external standard. The coupling constants (J) were given in hertz (Hz), and the signal multiplicities were described for the NMR data as s = singlet, d = doublet, t = triplet, dd = doublet of doublets, dt = doublet of triplets, qd = quartet of doublets, q = quartet, quint and p = pentet, m = multiplet, and br = broad. The ^{11}B NMR spectra of the synthesized borane–ammonia and borane-amines were recorded at 96 MHz on a Bruker 300 MHz NMR instrument and chemical shifts are reported relative to the external standard, BF_3-OEt_2 (δ = 0 ppm).

General procedure (1) for the preparation of amines and borane-amines from amides:

The preparation of dibenzylamine-borane from *N*-benzylbenzamide is typical. A 25 mL oven dried round bottom flask was charged with TiF_4 (1 mmol, 1 equiv.), amide (1 mmol, 1 equiv.), ammonia-borane (4 mmol, 4 equiv.), and a magnetic stirring bar respectively. 1,2-dichloroethane (DCE) (5 mL) was added, and a water-cooled condenser was attached to the flask before heating the solution using an oil bath to reflux. The reaction mixture was stirred and monitored by TLC until completion (~12h). On completion of the reaction, the crude mixture was brought to room temperature and quenched by slow addition of 1 M HCl, then transferred to a separatory funnel and extracted with dichloromethane (DCM) (3 × 5 mL). The combined organic layers were washed with brine (1 x 3 mL), dried over anhydrous sodium sulfate, filtered through cotton, and concentrated under aspirator vacuum using a rotary evaporator. Any remaining traces of solvent were removed by subjecting to high vacuum to afford the desired product.

General procedure (2) for the preparation of amines and borane-amines from carboxylic acids:

In a 25 ml round bottom flask containing a magnetic stir bar was added TiF_4 (0.1 mmol, 0.1 equiv.). Toluene (3 mL) was then added to the flask, followed by the addition of carboxylic acid (1.1 mmol, 1.1 equiv.) and amine (1 mmol, 1 equiv.). A reflux condenser was attached to the flask, and the reaction mixture was brought to reflux, using an oil bath, and monitored by TLC. After completion (~24 hours for aromatic carboxylic acids and ~12 hours for aliphatic carboxylic acids) the reaction was cooled to approximately 60°C, the condenser was briefly removed, and DCE (9 mL) was added to the reaction mixture followed by TiF_4 (1 mmol, 1 equiv.) and borane-ammonia (4 mmol, 4 equiv.). The condenser was reattached and the mixture was stirred, heated to reflux, and monitored by TLC once again. After completion of the reaction (~12 hours), the reaction mixture was quenched with 1 M HCl (3 mL), transferred to a separatory funnel, and extracted with DCM

(3 × 5 mL). The organic layer was washed with 5M sodium hydroxide solution (2 mL) and then brine (1 x 3 mL). The organic layer was dried with sodium sulfate, filtered through cotton, and concentrated under aspirator vacuum using a rotary evaporator. The reaction residue was diluted with methanol (3 mL) and condensed via rotary evaporation to remove toluene solvent if needed. Any remaining traces of solvent were removed by subjecting the solution to high vacuum for 30 min. Column chromatography was performed only if necessary to further purify the product using a hexane: ethyl acetate (v/v = 90:10) solvent system to afford the desired product.

Procedure (3) for the competitive reduction of tertiary and secondary amides:
A 25 mL oven dried round bottom flask was charged with TiF_4 (1 mmol, 1 equiv.), *N*-(phenylacetyl)morpholine (1 mmol, 1 equiv.), *N*-benzylphenylacetamide (1 mmol, 1 equiv.), borane-ammonia (4 mmol, 4 equiv.), and a magnetic stirring bar respectively. 1,2-dichloroethane (DCE) (5 mL) was added, and a water-cooled condenser was attached to the flask before heating the solution using an oil bath to reflux. The reaction mixture was stirred and monitored by TLC until completion (~12h). On completion of the reaction, the crude mixture was brought to room temperature and quenched by slow addition of 1 M HCl, then transferred to a separatory funnel and extracted with dichloromethane (DCM) (3 × 5 mL). The combined organic layers were washed with brine (1 x 3 mL), dried over anhydrous sodium sulfate, filtered through cotton, and concentrated under aspirator vacuum using a rotary evaporator. Any remaining traces of solvent were removed by subjecting to high vacuum to afford the desired product. Percent conversion was determined by ^{1}HNMR.

Procedure (4) for the recovery of the amine from the borane-amine:
A 25 mL oven dried round bottom flask was charged with borane-*N*-benzyl-2-phenylethan-1-amine (1 mmol, 1 equiv.) and a magnetic stirring bar. The amine borane was dissolved in tetrahydrofuran (5 mL) and BF_3-OEt_2 (2 mmol, 2 equiv.) was added. The reaction mixture was stirred for one hour before the addition of 1 M HCL (15 mL). The mixture was further stirred overnight before being transferred to a separatory funnel and extracted with dichloromethane (DCM) (2 × 3 mL). The aqueous layer was basified with 5M NaOH and extracted with DCM (3 x 2 mL). The organic layer was dried over anhydrous sodium sulfate, filtered through cotton, and concentrated under aspirator vacuum using a rotary evaporator. Any remaining traces of solvent were removed by subjecting to high vacuum to afford the desired amine product.

Procedure (5) for the one-pot preparation of N-benzyl-2-phenylethan-1-amine (2h):
In a 50 ml round bottom flask containing a magnetic stir bar was added TiF_4 (0.1 mmol, 0.1 equiv.). Toluene (3 mL) was then added to the flask, followed by the addition of carboxylic acid (1.1 mmol, 1.1 equiv.) and amine (1 mmol, 1 equiv.). A reflux condenser was attached to the flask, and the reaction mixture was brought to reflux, using an oil bath, and monitored by TLC. After completion (~12 hours) the reaction was cooled to approximately 60°C, the condenser was briefly removed, and DCE (9 mL) was added to the reaction mixture followed by TiF_4 (1 mmol, 1 equiv.) and ammonia-borane (4 mmol, 4 equiv.). The condenser was reattached and the mixture was stirred, heated to reflux, and monitored by TLC once again. After completion of the reaction (~12 hours), BF_3-OEt_2 (2.5 mmol, 2.5 equiv.) was added and allowed to stir until the reaction was complete by ^{11}BNMR (~3 hours). The reaction mixture condensed via rotary evaporation and the residue was dissolved in THF (10 mL), followed by the addition of 2 M HCl (10 mL). The mixture was further stirred overnight before being transferred to a separatory funnel and extracted with dichloromethane (DCM) (2 × 3 mL). The aqueous layer was basified with 5M NaOH and extracted

with DCM (3 x 2 mL). The organic layer was dried over anhydrous sodium sulfate, filtered through cotton, and concentrated under aspirator vacuum using a rotary evaporator. Any remaining traces of solvent were removed by subjecting to high vacuum to afford the desired amine product.

Procedure for the crystallization of borane-*N*-benzyl-1-cyclohexylmethanamine (2k-BH3):
Borane-*N*-benzyl-1-cyclohexylmethanamine (**2k**-BH_3) (0.5 mmol) was dissolved in hexanes (20 mL) and crystalized by slow evaporation. The structure was determined by single crystal x-ray crystallography utilizing a Bruker Quest diffractometer with a fixed chi angle, a sealed tube fine focus X-ray tube, single crystal curved graphite incident beam monochromator, a Photon II area detector and an Oxford Cryosystems low temperature device. Examination and data collection were performed with Mo Kα radiation (λ = 0.71073 Å) at 150 K. Complete crystallographic data, in CIF format, have been deposited with the Cambridge Crystallographic Data Centre. CCDC 2373783 contains the supplementary crystallographic data for this paper. These data can be obtained free of charge from The Cambridge Crystallographic Data Centre via www.ccdc.cam.ac.uk/data_request/cif.

Characterization of amine and amine-borane products:

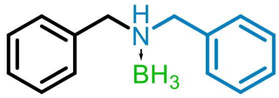

borane-dibenzylamine (2a-BH_3); The compound was prepared as described in *general procedure (1)* (white solid, mass = 202 mg, 96% yield); **^{1}H NMR** (400 MHz, $CDCl_3$) δ 7.4 – 7.3 (m, 6H), 7.2 – 7.1 (m, 4H), 4.0 (dd, *J* = 12.9, 5.3 Hz, 2H), 3.8 – 3.8 (m, 2H), 3.7 (s, 1H), 1.9 – 1.2 (m, 3H). **^{13}C {H} NMR** (101 MHz, $CDCl_3$) δ 134.4, 129.6, 129.0, 128.8, 58.6. **^{11}B NMR** (96 MHz, CDCl3) δ -14.9. Compound characterization is in accordance with previous reports.[3]

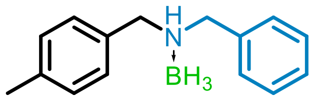

borane-*N*-benzyl-1-(p-tolyl)methanamine (2b-BH_3); The compound was prepared as described in *general procedure (1)* (white solid, mass = 216 mg, 96% yield); **^{1}H NMR** (300 MHz, $CDCl_3$) δ 7.3 – 7.3 (m, 3H), 7.1 (dd, *J* = 9.2, 4.5 Hz, 4H), 7.0 (d, *J* = 8.1 Hz, 2H), 3.9 (dt, *J* = 13.1, 5.3 Hz, 2H), 3.7 (td, *J* = 13.6, 6.3 Hz, 2H), 3.5 (s, 1H), 2.3 (s, 3H), 2.1 – 1.1 (m, 3H). **^{13}C {H} NMR** (75 MHz, $CDCl_3$) δ 138.7, 134.5, 131.4, 129.6, 129.5, 129.0, 128.8, 58.5, 21.2. **^{11}B NMR** (96 MHz, $CDCl_3$) δ -15.5.

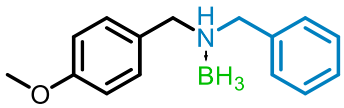

borane-*N*-benzyl-1-(4-methoxyphenyl)methanamine (2c-BH_3); The compound was prepared as described in *general procedure (1)* (white solid, mass = 233 mg, 97% yield); **^{1}H NMR** (300 MHz, CDCl3) δ 7.20 (dd, J = 5.4, 1.9 Hz, 3H), 7.08 (dd, J = 6.7, 2.9 Hz, 2H), 7.00 (d, J = 8.5 Hz, 2H), 6.72 (d, J = 8.7 Hz, 2H), 4.03 (s, 1H), 3.80 (ddd, J = 13.8, 8.7, 5.2 Hz, 2H), 3.73 – 3.51 (m, 5H), 1.63 (s, 3H). **^{13}C {H} NMR** (75 MHz, CDCl3) δ 159.5, 134.2, 131.1, 129.56, 128.57, 128.3, 126.1, 113.9, 57.8, 57.5, 55.1. **^{11}B NMR** (96 MHz, CDCl3) δ -15.1.

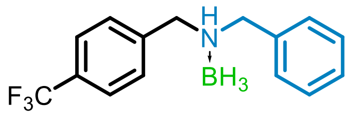

borane-*N*-benzyl-1-(4-(trifluoromethyl)phenyl)methanamine (2d-BH_3); The compound was prepared as described in *general procedure (1)* (white solid, mass = 265 mg, 95% yield); **^{1}H NMR** (300 MHz, $CDCl_3$) δ 7.5 (d, *J* = 8.1 Hz, 2H), 7.3 – 7.3 (m, 3H), 7.2 – 7.1 (m, 4H), 4.1 (dd, *J* = 13.2, 4.2 Hz, 1H), 4.0 (s, 1H), 3.8 (qd, *J* = 12.8, 5.6 Hz, 2H), 3.7 – 3.6 (m, 1H), 1.8 – 1.1 (m, 3H). **^{13}C {H} NMR** (75 MHz, $CDCl_3$) δ 138.0, 133.9, 130.2, 129.5, 129.1, 129.0, 125.7, 125.7, 59.3, 57.3. **^{19}F NMR** (282 MHz, $CDCl_3$) δ -62.81. **^{11}B NMR** (96 MHz, $CDCl_3$) δ -12.1 – -21.7 (m).

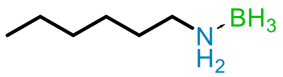

borane-hexylamine (2e-BH_3); The compound was prepared as described in *general procedure (1)* (colorless oil, mass = 106 mg, 92% yield); **^{1}H NMR** (300 MHz, $CDCl_3$) δ 3.6 (s, 1H), 2.7 (p, *J* = 7.2 Hz, 2H), 1.5 (p, *J* = 7.5 Hz, 3H), 1.3 – 1.2 (m, 8H), 0.9 – 0.8 (m, 3H). **^{13}C {H} NMR** (75 MHz, $CDCl_3$) δ 49.0, 31.3, 29.2, 26.2, 22.4, 13.9. **^{11}B NMR** (96 MHz, $CDCl_3$) δ -19.3 (q, *J* = 93.6 Hz).

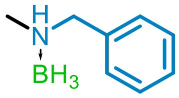

borane-*N*-methyl-1-phenylmethanamine (2f-BH_3); The compound was prepared as described in *general procedure (1)* (white solid, mass = 124 mg, 92% yield); ^{1}H NMR (400 MHz, $CDCl_3$) δ 7.4 – 7.3 (m, 3H), 7.3 – 7.3 (m, 2H), 4.3 (dd, *J* = 13.8, 3.0 Hz, 1H), 4.1 (s, 1H), 3.5 (dd, *J* = 13.8, 10.1 Hz, 1H), 2.4 (d, *J* = 5.8 Hz, 3H), 2.1 – 1.5 (m, 3H). **^{13}C {H} NMR** (101 MHz, $CDCl_3$) δ 134.1, 129.3, 129.0, 128.8, 60.9, 40.2. **^{11}B NMR** (96 MHz, $CDCl_3$) δ -13.8 (q, *J* = 93.8 Hz). Compound characterization is in accordance with previous reports.[4]

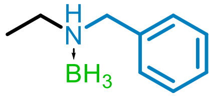

borane-*N*-benzylethanamine (2g-BH_3); The compound was prepared as described in *general procedure (1)* (clear oil, mass = 146 mg, 98% yield); **^{1}H NMR** (300 MHz, CDCl3) δ 7.35 – 7.20 (m, 5H), 4.11 (dd, J = 13.7, 3.5 Hz, 1H), 3.81 (s, 1H), 3.57 (dd, J = 13.7, 9.2 Hz, 1H), 2.72 (dtd, J = 14.5, 7.3, 3.3 Hz, 1H), 2.57 (dp, J = 12.1, 7.3 Hz, 1H), 1.72 (s, 3H), 1.12 (t, J = 7.3 Hz, 3H). **^{13}C {H} NMR** (75 MHz, CDCl3) δ 134.3, 129.3, 128.9, 128.6, 59.0, 47.4, 11.5. **^{11}B NMR** (96 MHz, CDCl3) δ -15.7 (d, J = 99.6 Hz).

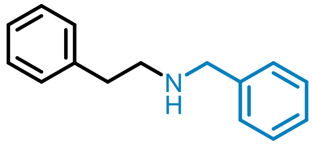

***N*-benzyl-2-phenylethan-1-amine (2h);** The compound was prepared as described in *general procedure (5)* (yellow oil, mass = 192 mg, 91% yield); **^{1}H NMR** (300 MHz, CDCl3) δ 7.31 – 6.99 (m, 10H), 3.70 (s, 2H), 2.90 – 2.77 (m, 2H), 2.72 (dd, J = 7.9, 4.8 Hz, 2H), 1.41 (s, 1H). **^{13}C {H} NMR** (75 MHz, $CDCl_3$) δ 140.2, 139.9, 128.6, 128.4, 128.3, 128.0, 126.8, 126.0, 53.8, 50.5, 36.3. Compound characterization is in accordance with previous reports.[5]

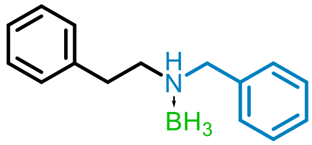

borane-*N*-benzyl-2-phenylethan-1-amine (2h-BH_3); The compound was prepared as described in *general procedure (1)* (white solid, mass = 209 mg, 93% yield); **^{1}H NMR** (300 MHz, CDCl3) δ 7.30 – 7.01 (m, 6H), 7.01 – 6.77 (m, 4H), 4.06 (dd, J = 13.6, 3.1 Hz, 1H), 3.63 (s, 1H), 3.49 (dd, J = 13.5, 9.5 Hz, 1H), 2.83 (dddd, J = 20.7, 18.1, 8.9, 5.7 Hz, 4H), 1.60 (s, 3H). **^{13}C {H} NMR** (75 MHz, CDCl3) δ 136.8, 133.6, 128.9, 128.7, 128.6, 128.4, 128.34, 126.6, 59.6, 53.2, 31.8. **^{11}B NMR** (96 MHz, CDCl3) δ -15.6.

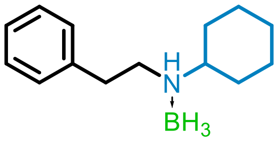

borane-*N*-phenethylcyclohexanamine (2i-BH_3); The compound was prepared as described in *general procedure (1)* (white solid, mass = 206 mg, 95% yield); **^{1}H NMR** (300 MHz, CDCl3) δ 7.34 – 7.04 (m, 5H), 3.09 (s, 1H), 3.03 – 2.81 (m, 4H), 2.77 – 2.54 (m, 1H), 1.85 (d, J = 12.1 Hz, 1H), 1.67 (d, J = 12.6 Hz, 3H), 1.57 – 1.26 (m, 5H), 1.09 (dddt, J = 24.0, 15.6, 12.4, 6.3 Hz, 4H). **^{13}C {H} NMR** (75 MHz, CDCl3) δ 137.2, 128.8, 128.6, 126.9, 62.8, 52.2, 32.6, 28.2, 28.0, 25.3, 25.1, 24.9. **^{11}B NMR** (96 MHz, CDCl3) δ -17.5.

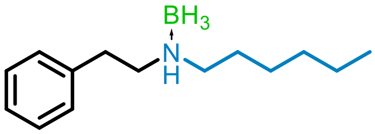

borane-*N*-phenethylhexan-1-amine (2j-BH_3); The compound was prepared as described in *general procedure (1)* (clear oil, mass = 208 mg, 95% yield); **^{1}H NMR** (300 MHz, $CDCl_3$) δ 7.35 – 7.07 (m, 5H), 3.20 – 2.82 (m, 5H), 2.81 – 2.52 (m, 2H), 1.77 (s, 1H), 1.51 – 1.41 (m, 3H), 1.27 – 0.89 (m, 7H), 0.78 (t, J = 6.8 Hz, 3H). **^{13}C {H} NMR** (75 MHz, $CDCl_3$) δ 137.2, 129.1, 128.7, 127.2, 55.8, 55.7, 32.3, 31.2, 26.1, 25.9, 22.4, 13.9. **^{11}B NMR** (96 MHz, $CDCl_3$) δ -16.3 (d, J = 112.5 Hz).

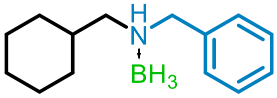

borane-*N*-benzyl-1-cyclohexylmethanamine (2k-BH_3); The compound was prepared as described in *general procedure (1)* (white solid, mass = 200 mg, 92% yield); **^{1}H NMR** (300 MHz, CDCl3) δ 7.39 – 7.11 (m, 5H), 4.16 (dd, J = 13.5, 3.1 Hz, 1H), 3.65 (s, 1H), 3.51 (dd, J = 13.4, 9.0 Hz, 1H), 2.39 (ddd, J = 17.7, 8.5, 4.0 Hz, 2H), 1.81 (s, 2H), 1.52 (d, J = 9.6 Hz, 6H), 1.24 – 0.86 (m, 4H), 0.70 (qd, J = 13.3, 4.3 Hz, 1H), 0.41 (qd, J = 12.2, 3.3 Hz, 1H). **^{13}C {H} NMR** (75 MHz, CDCl3) δ 134.2, 129.2, 128.8, 128.5, 60.3, 59.8, 33.8, 30.8, 29.8, 25.9, 25.3, 25.0. **^{11}B NMR** (96 MHz, CDCl3) δ -15.1.

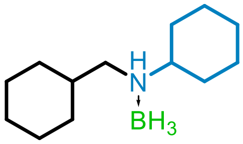

borane-*N*-(cyclohexylmethyl)cyclohexanamine (2l-BH_3); The compound was prepared as described in *general procedure (1)* (white solid, mass = 197 mg, 94% yield); **^{1}H NMR** (300 MHz, $CDCl_3$) δ 3.24 (s, 1H), 2.71 (td, J = 11.6, 2.6 Hz, 1H), 2.56 – 2.36 (m, 2H), 1.92 (d, J = 12.2 Hz, 1H), 1.81 – 1.54 (m, 12H), 1.53 – 1.41 (m, 2H), 1.27 – 1.02 (m, 7H), 0.84 (ddd, J = 23.7, 11.9, 9.0 Hz, 2H). **^{13}C {H} NMR** (75 MHz, $CDCl_3$) δ 62.6, 57.9, 34.0, 30.8, 30.3, 28.3, 28.1, 26.0, 25.4, 25.3, 25.2, 25.0. **^{11}B NMR** (96 MHz, $CDCl_3$) δ -17.8 (d, J = 92.9 Hz).

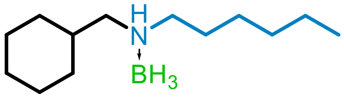

borane-*N*-(cyclohexylmethyl)hexan-1-amine (2m-BH_3); The compound was prepared as described in *general procedure (1)* (white solid, mass = 192 mg, 91% yield); **^{1}H NMR** (300 MHz, $CDCl_3$) δ 3.1 (s, 1H), 2.8 – 2.6 (m, 1H), 2.6 – 2.5 (m, 2H), 2.4 (ddd, *J* = 12.7, 7.9, 5.2 Hz, 1H), 1.7 – 1.5 (m, 8H), 1.3 – 1.2 (m, 9H), 0.9 – 0.8 (m, 5H). **^{13}C {H} NMR** (75 MHz, $CDCl_3$) δ 62.0, 55.9, 34.0, 31.4, 30.84, 30.6, 26.4, 26.3, 25.5, 25.4, 22.5, 14.0. **^{11}B NMR** (96 MHz, $CDCl_3$) δ -15.8 (q, *J* = 92.2 Hz).

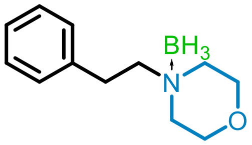

borane-4-phenethylmorpholine (2n-BH_3); The compound was prepared as described in *general procedure (1)* (white solid, mass = 183 mg, 89% yield); **^{1}H NMR** (300 MHz, $CDCl_3$) δ 7.17 (dt, J = 23.4, 7.4 Hz, 5H), 4.09 (t, J = 10.1 Hz, 2H), 3.60 (d, J = 12.6 Hz, 2H), 3.19 – 3.00 (m, 2H), 2.92 (d, J = 12.0 Hz, 4H), 2.65 (t, J = 9.3 Hz, 2H), 1.68 (s, 3H). **^{13}C {H} NMR** (75 MHz, $CDCl_3$) δ 137.9, 128.6, 128.5, 126.4, 67.1, 61.6, 57.8, 29.2. **^{11}B NMR** (96 MHz, $CDCl_3$) δ -13.6 (d, J = 110.2 Hz).

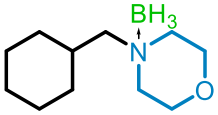

borane-4-(cyclohexylmethyl)morpholine (2o-BH_3); The compound was prepared as described in *general procedure (1)* (white solid, mass = 189 mg, 96% yield); **^{1}H NMR** (300 MHz, $CDCl_3$) δ 4.14 (ddd, J = 12.5, 9.9, 2.6 Hz, 2H), 3.62 (dt, J = 12.6, 3.5 Hz, 2H), 2.88 (dt, J = 12.2, 2.4 Hz, 2H), 2.67 (ddd, J = 12.8, 9.9, 3.6 Hz, 2H), 2.57 (d, J = 4.0 Hz, 2H), 2.01 (ddp, J = 11.5, 7.8, 3.9 Hz, 1H), 1.93 – 1.70 (m, 3H), 1.70 – 1.36 (m, 4H), 1.26 (qt, J = 13.5, 3.5 Hz, 3H), 1.15 – 0.90 (m, 3H). **^{13}C {H} NMR** (75 MHz, $CDCl_3$) δ 73.3, 61.8, 57.8, 34.3, 31.9, 26.0, 25.6. **^{11}B NMR** (96 MHz, $CDCl_3$) δ -13.8 (d, J = 99.5 Hz).

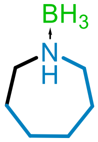

borane-azepane (2p-BH_3); The compound was prepared as described in *general procedure (1)* (white solid, mass = 100 mg, 89% yield); **^{1}H NMR** (300 MHz, $CDCl_3$) δ 3.3 – 3.1 (m, 2H), 2.8 – 2.6 (m, 2H), 1.9 – 1.5 (m, 9H). **^{13}C {H} NMR** (75 MHz, $CDCl_3$) δ 55.2, 27.2, 26.6. **^{11}B NMR** (96 MHz, $CDCl_3$) δ -12.8 – -18.4 (m). Compound characterization is in accordance with previous reports.[3]

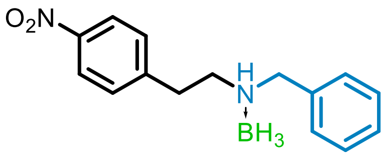

borane-*N*-benzyl-2-(4-nitrophenyl)ethan-1-amine (2q-BH_3); The compound was prepared as described in *general procedure (1)* (orange solid, mass = 248 mg, 92% yield); **^{1}H NMR** (300 MHz, CDCl3) δ 7.98 (d, J = 8.7 Hz, 2H), 7.27 (dd, J = 5.0, 2.0 Hz, 3H), 7.16 – 6.98 (m, 4H), 4.28 – 4.11 (m, 1H), 3.64 (s, 1H), 3.55 (dd, J = 13.3, 9.7 Hz, 1H), 3.14 – 3.01 (m, 2H), 3.01 – 2.73 (m, 2H), 1.75 (s, 3H). **^{13}C {H} NMR** (75 MHz, CDCl3) δ 146.8, 144.9, 133.5, 129.4, 129.1, 129.1, 128.9, 123.8, 60.1, 52.7, 31.9. **^{11}B NMR** (96 MHz, CDCl3) δ -15.7.

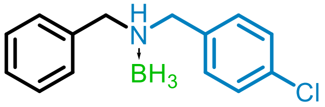

borane-*N*-benzyl-1-(4-chlorophenyl)methanamine (2r-BH_3); The compound was prepared as described in *general procedure (1)* (white solid, mass = 238 mg, 97% yield); **^{1}H NMR** (300 MHz, CDCl3) δ 7.34 – 7.08 (m, 7H), 7.00 (d, J = 8.4 Hz, 2H), 4.14 (s, 1H), 3.93 (dd, J = 13.3, 4.7 Hz, 1H), 3.76 (dd, J = 13.0, 5.8 Hz, 1H), 3.61 (ddd, J = 13.4, 9.3, 6.5 Hz, 2H), 1.68 (s, 3H). **^{13}C {H} NMR** (75 MHz, CDCl3) δ 134.5, 133.9, 132.4, 131.2, 129.6, 128.8, 128.8, 128.7, 58.6, 57.0. **^{11}B NMR** (96 MHz, CDCl3) δ -14.9.

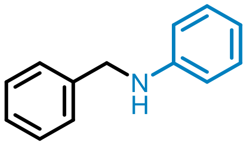

***N*-benzylaniline (2s);** The compound was prepared as described in *general procedure (1)* (colorless oil, mass = 172 mg, 94% yield); **^{1}H NMR** (400 MHz, $CDCl_3$) δ 7.5 – 7.3 (m, 4H), 7.3 – 7.3 (m, 1H), 7.2 (dd, *J* = 8.6, 7.3 Hz, 2H), 6.7 (tt, *J* = 7.3, 1.1 Hz, 1H), 6.7 (dd, *J* = 8.7, 1.1 Hz, 2H), 4.3 (s, 2H), 4.1 (s, 1H). **^{13}C {H} NMR** (101 MHz, $CDCl_3$) δ 148.1, 139.4, 129.2, 128.6, 127.5, 127.2, 117.6, 112.8, 48.3. Compound characterization is in accordance with previous reports.[6]

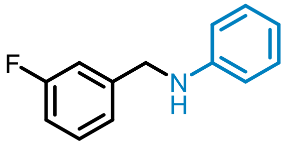

***N*-(3-fluorobenzyl)aniline (2t);** The compound was prepared as described in *general procedure (1)* (brown solid, mass = 195 mg, 97% yield); **^{1}H NMR** (300 MHz, CDCl3) δ 7.17 (td, J = 7.9, 5.8 Hz, 1H), 7.11 – 6.92 (m, 4H), 6.92 – 6.75 (m, 1H), 6.62 (tt, J = 7.3, 1.1 Hz, 1H), 6.56 – 6.40 (m, 2H), 4.20 (s, 2H), 3.87 (d, J = 62.1 Hz, 1H). **^{13}C {H} NMR** (75 MHz, CDCl3) δ 163.1 (d, *J* = 246.1 Hz), 147.7, 142.3 (d, *J* = 6.8 Hz), 130.0 (d, *J* = 8.1 Hz), 129.2, 122.7, 117.8, 114.2 (d, *J* = 9.5 Hz), 113.9 (d, *J* = 9.1 Hz), 112.8, 47.7. **^{19}F NMR** (282 MHz, CDCl3) δ -112.85 (td, J = 9.3, 5.8 Hz). Compound characterization is in accordance with previous reports.[7]

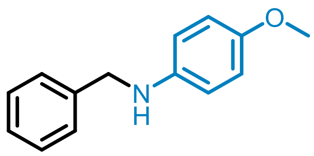

***N*-benzyl-4-methoxyaniline (2u);** The compound was prepared as described in *general procedure (1)* (brown oil, mass = 207 mg, 97% yield); **^{1}H NMR** (300 MHz, $CDCl_3$) δ 7.30 – 7.10 (m, 5H), 6.66 (d, J = 8.9 Hz, 2H), 6.47 (d, J = 8.9 Hz, 2H), 4.14 (s, 2H), 3.60 (s, 3H), 3.54 (s, 1H). **^{13}C {H} NMR** (75 MHz, $CDCl_3$) δ 152.1, 142.4, 139.6, 128.5, 127.4, 127.0, 114.8, 114.0, 55.7, 49.1. Compound characterization is in accordance with previous reports.[8]

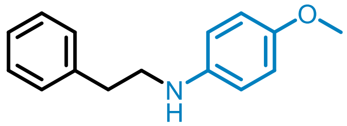

4-methoxy-*N*-phenethylaniline (2v); The compound was prepared as described in *general procedure (1)* (brown oil, mass = 204 mg, 90% yield); **^{1}H NMR** (300 MHz, CDCl3) δ 7.27 – 7.17 (m, 2H), 7.12 (td, J = 6.7, 1.8 Hz, 3H), 6.79 – 6.61 (m, 2H), 6.57 – 6.41 (m, 2H), 3.63 (s, 3H), 3.24 (t, J = 7.0 Hz, 3H), 2.78 (t, J = 7.0 Hz, 2H). **^{13}C {H} NMR** (75 MHz, $CDCl_3$) δ 152.1, 142.2, 139.3, 128.7, 128.5, 126.3, 114.8, 114.3, 55.7, 46.0, 35.5. Compound characterization is in accordance with previous reports.[9]

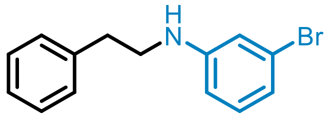

3-bromo-*N*-phenethylaniline (2w); The compound was prepared as described in *general procedure (1)* (brown oil, mass = 273 mg, 99% yield); **^{1}H NMR** (300 MHz, $CDCl_3$) δ 7.30 – 7.10 (m, 5H), 6.93 (t, J = 8.0 Hz, 1H), 6.73 (ddd, J = 7.8, 1.8, 0.9 Hz, 1H), 6.67 (t, J = 2.1 Hz, 1H), 6.43 (ddd, J = 8.2, 2.3, 0.9 Hz, 1H), 3.31 (t, J = 7.0 Hz, 2H), 2.84 (t, J = 6.9 Hz, 2H). **^{13}C {H} NMR** (75 MHz, $CDCl_3$) δ 149.1, 138.8, 130.4, 128.6, 128.5, 126.4, 123.2, 119.9, 115.2, 111.4, 44.5, 35.1. Compound characterization is in accordance with previous reports.[10]

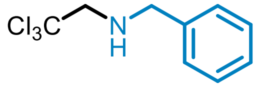

***N*-benzyl-2,2,2-trichloroethan-1-amine (2x);** The compound was prepared as described in *general procedure (1)* (white solid, mass = 214 mg, 90% yield); **^{1}H NMR** (300 MHz, CDCl3) δ 7.41 – 7.10 (m, 5H), 4.00 (s, 2H), 3.47 (s, 2H), 2.12 (s, 1H). **^{13}C {H} NMR** (75 MHz, CDCl3) δ 139.6, 128.5, 127.9, 127.3, 100.5, 67.4, 53.1.

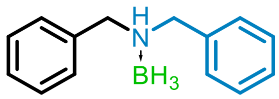

borane-dibenzylamine (3a-BH_3); The compound was prepared as described in *general procedure (2)* (white solid, mass = 188 mg, 89% yield). **^{1}H NMR**, **^{13}C {H} NMR,** and **^{11}B NMR** characterization is in accordance with compound 2a.

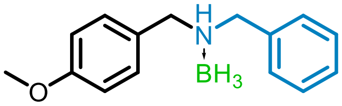

borane-*N*-benzyl-1-(4-methoxyphenyl)methanamine (3c-BH_3); The compound was prepared as described in *general procedure (2)* (white solid, mass = 200 mg, 83% yield); **^{1}H NMR**, **^{13}C {H} NMR,** and **^{11}B NMR** characterization is in accordance with compound 2c.

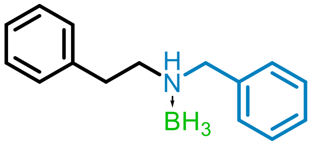

borane-*N*-benzyl-2-phenylethan-1-amine (3h-BH_3); The compound was prepared as described in *general procedure (2)* (white solid, mass = 221 mg, 98% yield) and the large-scale *procedure (3)* (white solid, mass = 142 mg, 63% yield); **^{1}H NMR**, **^{13}C {H} NMR,** and **^{11}B NMR** characterization is in accordance with compound 2h.

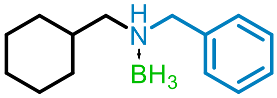

borane-*N*-benzyl-1-cyclohexylmethanamine (3k-BH_3); The compound was prepared as described in *general procedure (2)* (white solid, mass = 210 mg, 97% yield); **^{1}H NMR**, **^{13}C {H} NMR,** and **^{11}B NMR** characterization is in accordance with compound 2k.

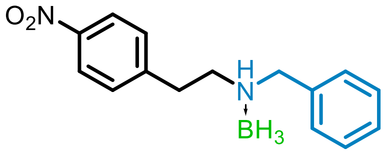

borane-*N*-benzyl-2-(4-nitrophenyl)ethan-1-amine (3q-BH_3); The compound was prepared as described in *general procedure (2)* (orange solid, mass = 111 mg, 41% yield); **^{1}H NMR**, **^{13}C {H} NMR,** and **^{11}B NMR** characterization is in accordance with compound 2q.

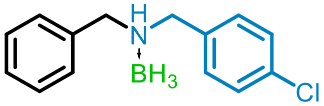

borane-*N*-benzyl-1-(4-chlorophenyl)methanamine (3r-BH_3); The compound was prepared as described in *general procedure (2)* (white solid, mass = 125 mg, 51% yield); **^{1}H NMR**, **^{13}C {H} NMR,** and **^{11}B NMR** characterization is in accordance with compound 2r.

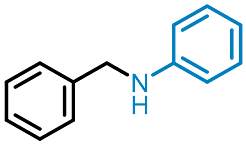

***N*-benzylaniline (3s);** The compound was prepared as described in *general procedure (2)* (colorless oil, mass = 159 mg, 87% yield); **^{1}H NMR**, **^{13}C {H} NMR,** and **^{11}B NMR** characterization is in accordance with compound 2s.

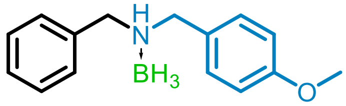

borane-*N*-benzyl-1-(4-methoxyphenyl)methanamine (3y-BH_3); The compound was prepared as described in *general procedure (2)* (white solid, mass = 142 mg, 59% yield); **^{1}H NMR**, **^{13}C {H} NMR,** and **^{11}B NMR** characterization is in accordance with compound 2c.

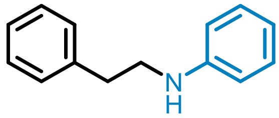

***N*-phenethylaniline (3z);** The compound was prepared as described in *general procedure (2)* (brown oil, mass = 134 mg, 68% yield); **^{1}H NMR** (300 MHz, CDCl3) δ 7.42 – 7.34 (m, 2H), 7.33 – 7.21 (m, 5H), 6.79 (tt, J = 7.3, 1.1 Hz, 1H), 6.66 (dd, J = 8.6, 1.1 Hz, 2H), 3.61 (s, 1H), 3.44 (t, J = 7.0 Hz, 2H), 2.95 (t, J = 7.1 Hz, 2H). **^{13}C {H} NMR** 13C NMR (75 MHz, CDCl3) δ 147.9, 139.2, 129.2, 128.7, 128.5, 126.3, 117.3, 112.9, 44.9, 35.4. Compound characterization is in accordance with previous reports.[11]

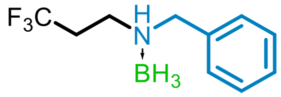

borane-benzenemethanamine, *N*-(3,3,3-trifluoropropyl) (3aa-BH_3); The compound was prepared as described in *general procedure (2)* (white solid, mass = 171 mg, 79% yield); **^{1}H NMR** (400 MHz, $CDCl_3$) δ 7.5 – 7.4 (m, 3H), 7.3 (dd, *J* = 7.3, 2.1 Hz, 2H), 4.3 (dd, *J* = 13.9, 3.2 Hz, 1H), 3.8 (s, 1H), 3.6 (dd, *J* = 13.9, 9.6 Hz, 1H), 3.1 – 2.9 (m, 1H), 2.8 – 2.7 (m, 1H), 2.7 – 2.6 (m, 1H), 2.6 – 2.5 (m, 1H), 2.0 – 1.1 (m, 3H). **^{13}C {H} NMR** (101 MHz, $CDCl_3$) δ 133.2, 129.4, 129.3, 129.2, 60.3, 45.8, 30.7, 30.4. **^{19}F NMR** (376 MHz, $CDCl_3$) δ -66.7 (t, *J* = 10.7 Hz). **^{11}B NMR** (128 MHz, $CDCl_3$) δ -15.76 (q, *J* = 96.8, 93.7 Hz).

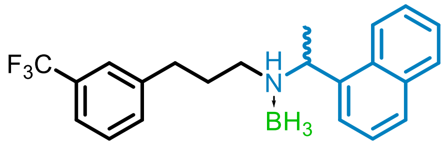

***N*-(1-(naphthalen-1-yl)ethyl)-3-(3-(trifluoromethyl)phenyl)propan-1-amine borane (4a-BH_3);** The compound was prepared as described in *general procedure (1)* (yellow oil, mass = 368 mg, 99% yield for mixture)

Characterization of Mixture:

^{1}H NMR (300 MHz, $CDCl_3$) δ 7.93 – 7.72 (m, 3H), 7.70 – 7.57 (m, 1H), 7.48 (ddd, J = 6.9, 4.5, 1.6 Hz, 2H), 7.40 – 7.35 (m, 1H), 7.30 – 7.23 (m, 1H), 7.18 – 7.05 (m, 2H), 7.00 – 6.95 (m, 1H), 5.26 – 4.50 (m, 1H), 3.65 (d, J = 28.1 Hz, 1H), 2.69 – 2.45 (m, 2H), 2.31 (dt, J = 8.3, 6.2 Hz, 2H), 2.23 – 1.94 (m, 2H), 1.84 (tdd, J = 8.9, 5.6, 2.9 Hz, 2H), 1.68 (dd, J = 11.3, 6.9 Hz, 3H), 1.56 (s, 1H). **^{13}C {H} NMR** (75 MHz, $CDCl_3$) δ 141.2, 140.9, 136.5, 134.2, 133.9, 133.6, 131.3, 130.8, 129.9, 129.8, 129.5, 129.0, 128.7, 128.2, 127.1, 126.4, 126.1, 125.6, 125.4, 125.2, 124.9, 124.7, 123.4, 122.9, 121.5, 121.3, 58.4, 54.0, 46.7, 32.6, 32.0, 28.2, 27.8, 20.2, 10.6. **^{19}F NMR** (282 MHz, $CDCl_3$) δ -62.5. **^{11}B NMR** (128 MHz, $CDCl_3$) δ -16.8.

Characterization of Component 1:

^{1}H NMR (300 MHz, $CDCl_3$) δ 7.97 (d, J = 8.5 Hz, 1H), 7.86 (dd, J = 8.1, 1.4 Hz, 1H), 7.78 (dd, J = 6.3, 3.2 Hz, 1H), 7.59 (ddd, J = 8.5, 6.9, 1.5 Hz, 1H), 7.49 (ddd, J = 8.1, 6.9, 1.1 Hz, 1H), 7.36 (q, J = 3.7 Hz, 2H), 7.24 (d, J = 7.8 Hz, 1H), 7.07 (t, J = 7.7 Hz, 1H), 6.96 (s, 1H), 6.74 (d, J = 7.7 Hz, 1H), 5.13 (qd, J = 7.0, 1.5 Hz, 1H), 3.59 (s, 1H), 2.61 – 2.35 (m, 2H), 2.29 – 1.74 (m, 5H),

1.65 (d, J = 7.0 Hz, 3H), 1.38 (td, J = 14.2, 7.3 Hz, 2H). **^{13}C {H} NMR** (75 MHz, $CDCl_3$) δ 140.9, 134.2, 133.6, 131.2, 130.7, 130.3, 129.8, 129.7, 128.7, 128.1, 126.3, 124.9, 124.6, 124.5, 122.9, 122.8, 121.2, 58.3, 46.6, 32.0, 28.1, 10.6. **^{19}F NMR** (96 MHz, $CDCl_3$) δ -62.5. **^{11}B NMR** (96 MHz, $CDCl_3$) δ -16.3.

Characterization of Component 2:

^{1}H NMR (300 MHz, $CDCl_3$) δ 7.94 – 7.80 (m, 2H), 7.80 – 7.71 (m, 1H), 7.55 – 7.32 (m, 4H), 7.27 (d, J = 7.8 Hz, 1H), 7.20 – 7.05 (m, 2H), 6.97 (d, J = 7.6 Hz, 1H), 4.73 – 4.51 (m, 1H), 3.74 (s, 1H), 2.72 – 2.46 (m, 2H), 2.30 (h, J = 7.4 Hz, 2H), 2.12 – 1.76 (m, 3H), 1.70 (d, J = 6.8 Hz, 3H), 1.55 (s, 2H). **^{13}C {H} NMR** (75 MHz, $CDCl_3$) δ 141.3, 136.5, 133.9, 131.3, 130.8, 130.3, 129.5, 129.0, 128.7, 127.1, 126.2, 125.4, 124.8, 124.7, 123.5, 122.9, 122.9, 121.5, 59.1, 54.0, 32.6, 27.8, 20.2. **^{19}F NMR** (282 MHz, $CDCl_3$) δ -62.6. **^{11}B NMR** (96 MHz, $CDCl_3$) δ -17.2.

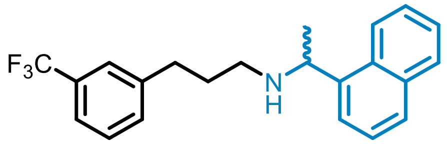

***N*-(1-(naphthalen-1-yl)ethyl)-3-(3-(trifluoromethyl)phenyl)propan-1-amine (cinacalcet) (4a);** The compound was prepared as described in *general procedure (1) and (4)* (clear oil, mass = 336 mg, 94% yield); **^{1}H NMR** (300 MHz, $CDCl_3$) δ 8.32 (d, J = 8.2 Hz, 1H), 7.98 (d, J = 9.6 Hz, 1H), 7.86 (d, J = 8.1 Hz, 1H), 7.77 (d, J = 6.6 Hz, 1H), 7.72 – 7.46 (m, 5H), 7.40 (q, J = 7.8 Hz, 2H), 4.73 (q, J = 6.6 Hz, 1H), 2.96 – 2.59 (m, 4H), 1.92 (p, J = 7.4 Hz, 2H), 1.78 (s, 1H), 1.61 (d, J = 6.6 Hz, 3H). **^{13}C {H} NMR** (75 MHz, $CDCl_3$) δ 143.0, 141.1, 133.9, 131.7, 131.2, 130.6, 130.2, 128.9, 128.5, 127.1, 125.6, 125.6, 125.2, 124.9, 124.9, 122.8, 122.5, 122.5, 122.4, 53.6, 47.1, 33.2, 31.7, 23.4. **^{19}F NMR** (282 MHz, $CDCl_3$) δ -62.2.

Compound characterization is in accordance with previous reports.[12]

References:

(1) Ramachandran, P. V.; Kulkarni, A. S. Water-Promoted, Safe and, Scalable Preparation of Ammonia Borane. *Int. J. Hydrog. Energy* **2017,** *42*, 1451-1455.

(2) Alawaed, A. A.; Ramachandran, P. V. TiF_4-Catalyzed Direct Amidation of Carboxylic Acids and Amino Acids with Amines. *Org. Biomol. Chem.* **2024,** *22*, 1915-1919.

(3) Ramachandran, P. V.; Hamann, H. J.; Mishra, S. Aminoboranes Via Tandem Iodination/Dehydroiodination for One-Pot Borylation. *ACS Omega* **2022,** *7*, 14377-14389.

(4) Jaska, C. A.; Temple, K.; Lough, A. J.; Manners, I. Transition Metal-Catalyzed Formation of Boron−Nitrogen Bonds: Catalytic Dehydrocoupling of Amine-Borane Adducts to Form Aminoboranes and Borazines. *J. Am. Chem. Soc.* **2003,** *125*, 9424-9434.

(5) Xie, Q.; Zhang, R.; Dong, G. Programmable Amine Synthesis Via Iterative Boron Homologation. *Angew. Chem., Int. Ed.* **2023,** *62*, e202307118.

(6) Ding, M.; Chang, J.; Mao, J.-X.; Zhang, J.; Chen, X. Pncnp Pincer Platinum Chloride Complex as a Catalyst for the Hydrosilylation of Unsaturated Carbon-Heteroatom Bonds. *Organometallics* **2022,** *41*, 3636-3644.

(7) Su, W.; Xie, Y.; Chen, M.; Xu, Z.; Shi, D.; Yu, W.; Jiang, F.; Yu, H. Catalytic N-Alkylation of Amines with Aldehydes by a Molecular Mo Oxide Catalyst. *Eur. J. Inorg. Chem.* **2023,** *26*, e202200751.

(8) Zhu, C.; Kale, A. P.; Yue, H.; Rueping, M. Redox-Neutral Cross-Coupling Amination with Weak N-Nucleophiles: Arylation of Anilines, Sulfonamides, Sulfoximines, Carbamates, and Imines Via Nickelaelectrocatalysis. *JACS Au* **2021,** *1*, 1057-1065.

(9) Qin, C.; Shen, T.; Tang, C.; Jiao, N. $FeCl_2$-Promoted Cleavage of the Unactivated C-C Bond of Alkylarenes and Polystyrene: Direct Synthesis of Arylamines. *Angew. Chem., Int. Ed.* **2012,** *51*, 6971-6975.

(10) Larrosa, M.; Guerrero, C.; Rodríguez, R.; Cruces, J. Selective Copper-Promoted Cross-Coupling of Aromatic Amines with Alkyl Boronic Acids. *Synlett* **2010,** *2010*, 2101-2105.

(11) Li, W.; Shi, J.; He, D.; Chen, H. Cascade Performance of Nitroarenes with Alcohols Boosted by a Hollow Flying Saucer-Shaped Ni–Al_2O_3 Catalyst Via a Mof-Templated Strategy Induced by the Kirkendall Effect. *Inorg. Chem.* **2023,** *62*, 21470-21478.

(12) I. Sorribes, K. Junge and M. Beller, Direct Catalytic *N*-Alkylation of Amines with Carboxylic Acids. *J. Am. Chem. Soc.*, **2014**, *136*, 14314-14319.

NMR spectra of amine and borane-amine products:

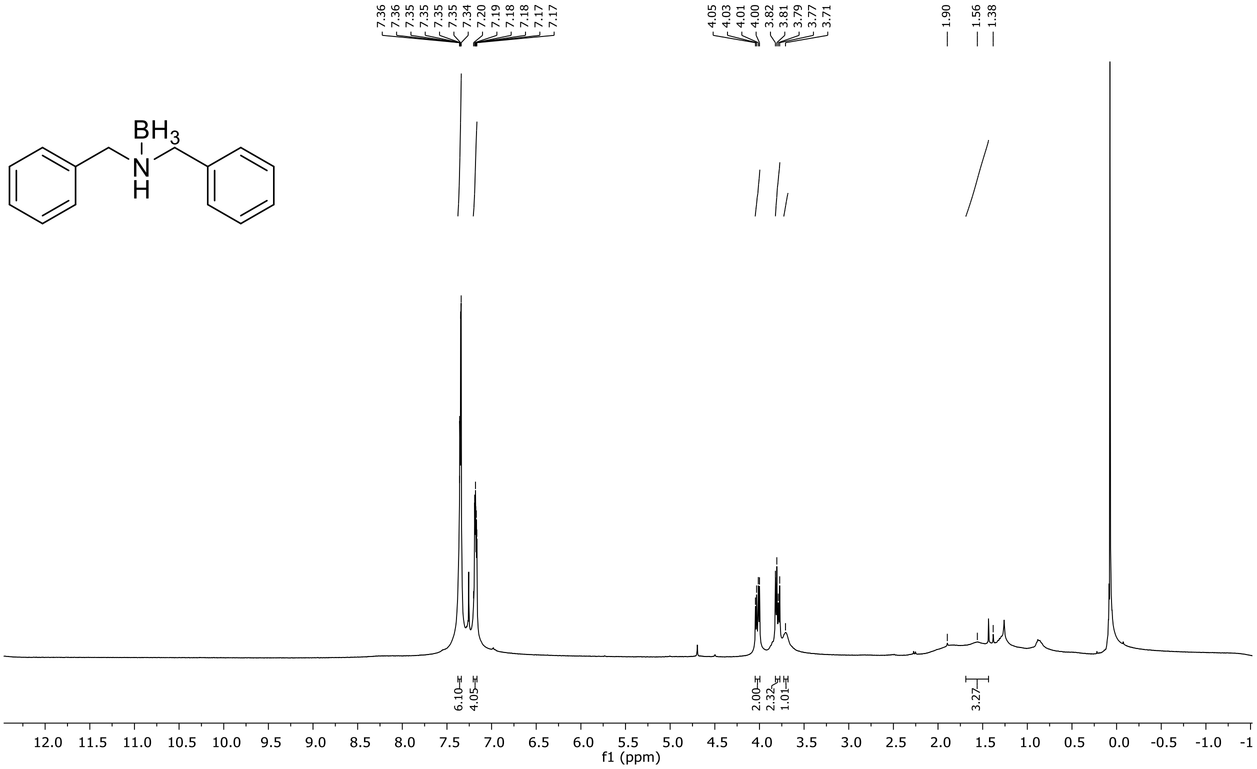

^{1}H NMR (400 MHz, $CDCl_3$) borane-dibenzylamine (**2a-BH$_3$**)

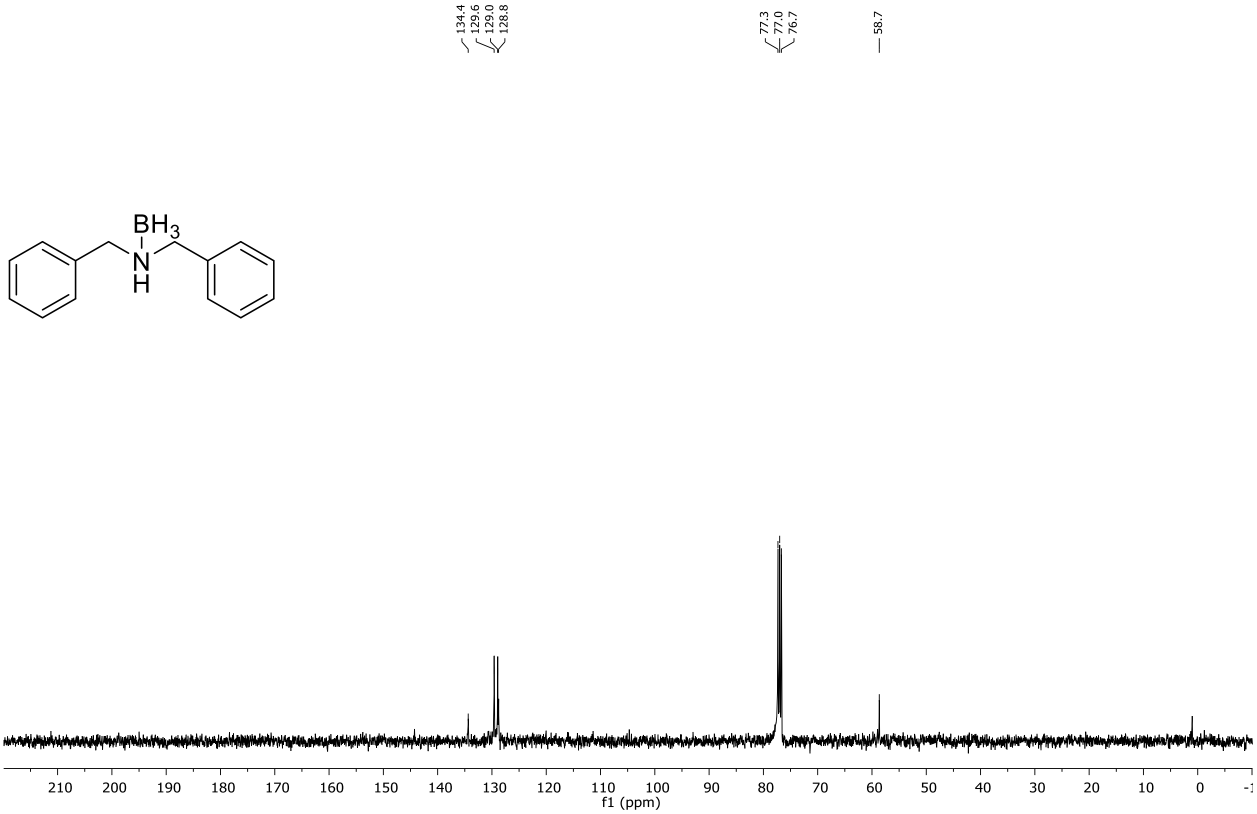

^{13}C {H} NMR (101 MHz, $CDCl_3$) borane-dibenzylamine (**2a-BH$_3$**)

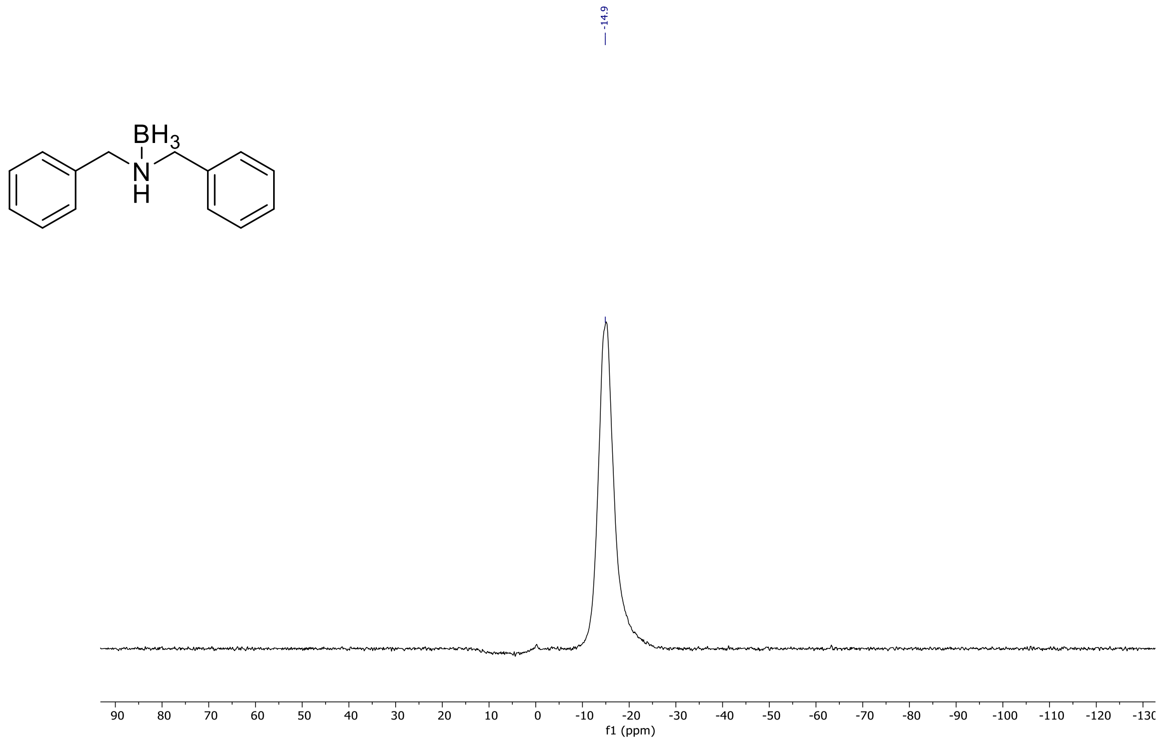

^{11}B NMR (96 MHz, $CDCl_3$) borane-dibenzylamine (**2a-BH$_3$**)

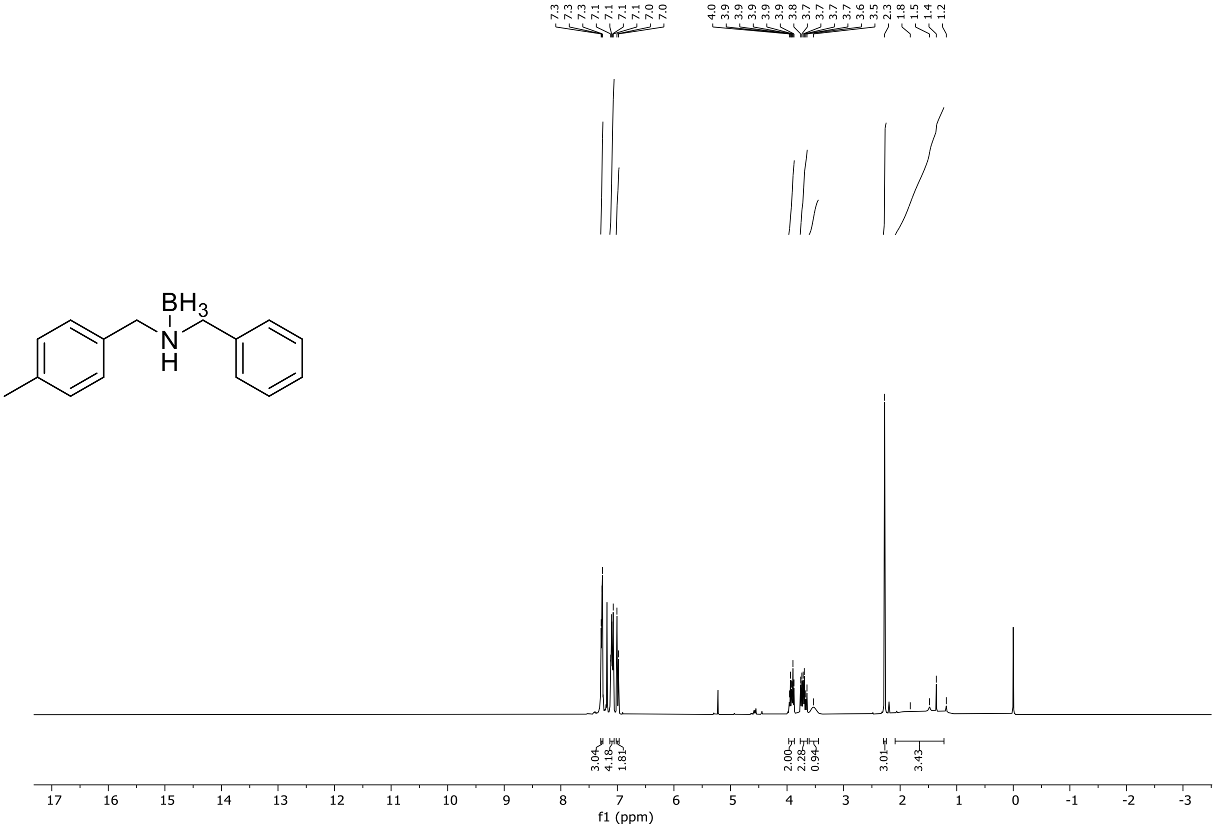

^{1}H NMR (300 MHz, $CDCl_3$) borane-*N*-benzyl-1-(p-tolyl)methanamine (**2b-BH$_3$**)

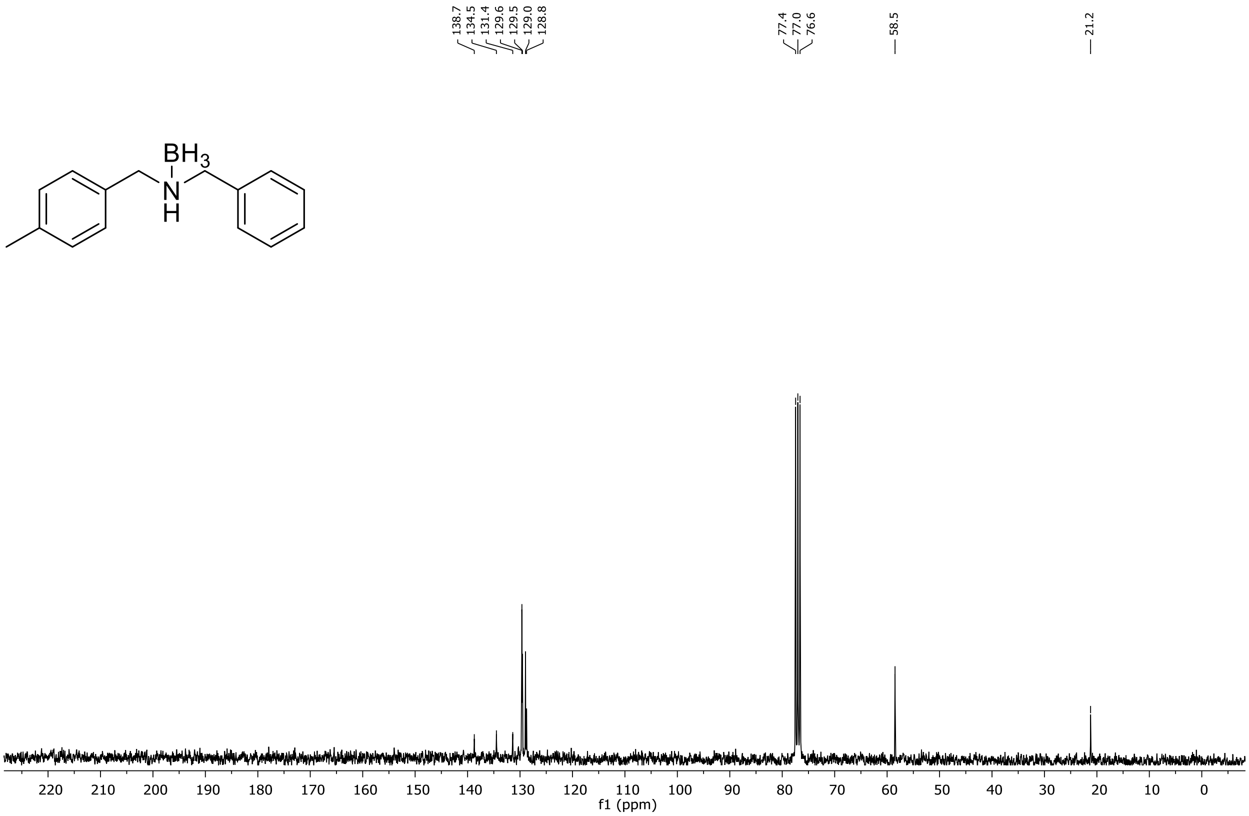

^{13}C {H} NMR (75 MHz, $CDCl_3$) borane-*N*-benzyl-1-(p-tolyl)methanamine (**2b-BH$_3$**)

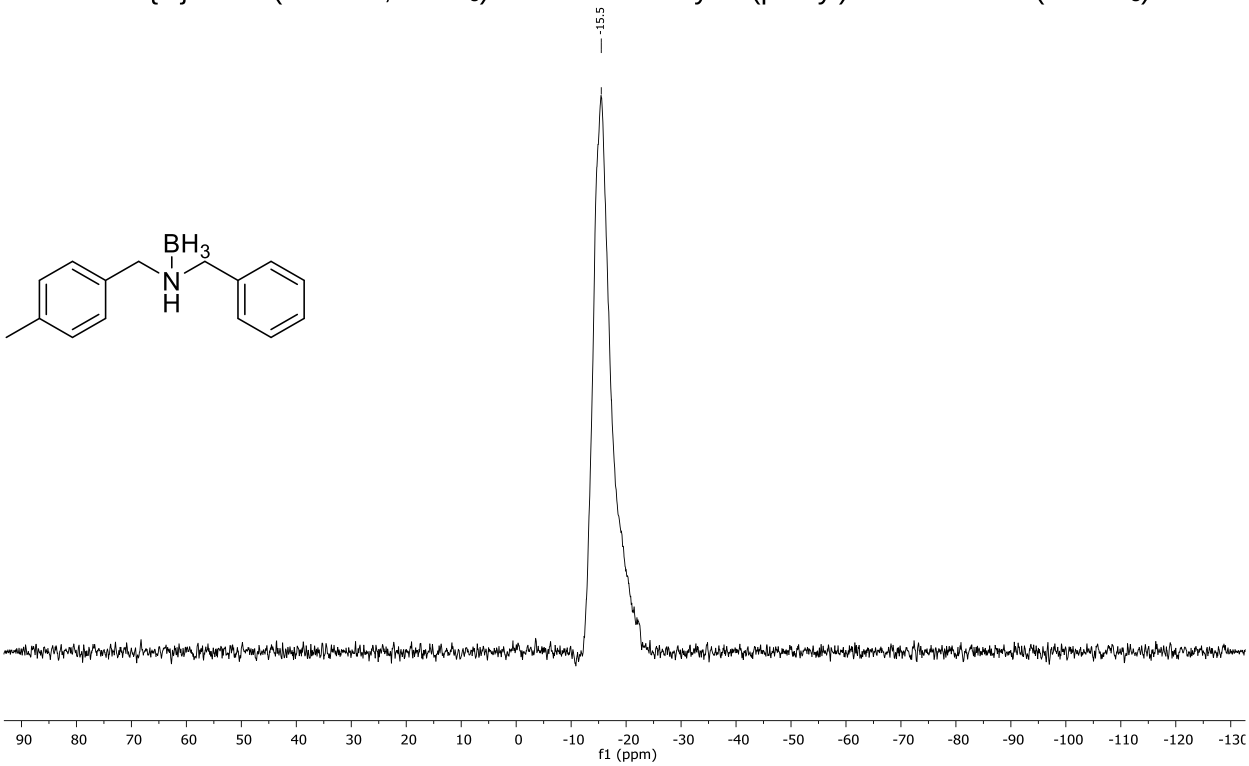

^{11}B NMR (96 MHz, $CDCl_3$) borane-*N*-benzyl-1-(p-tolyl)methanamine (**2b-BH$_3$**)

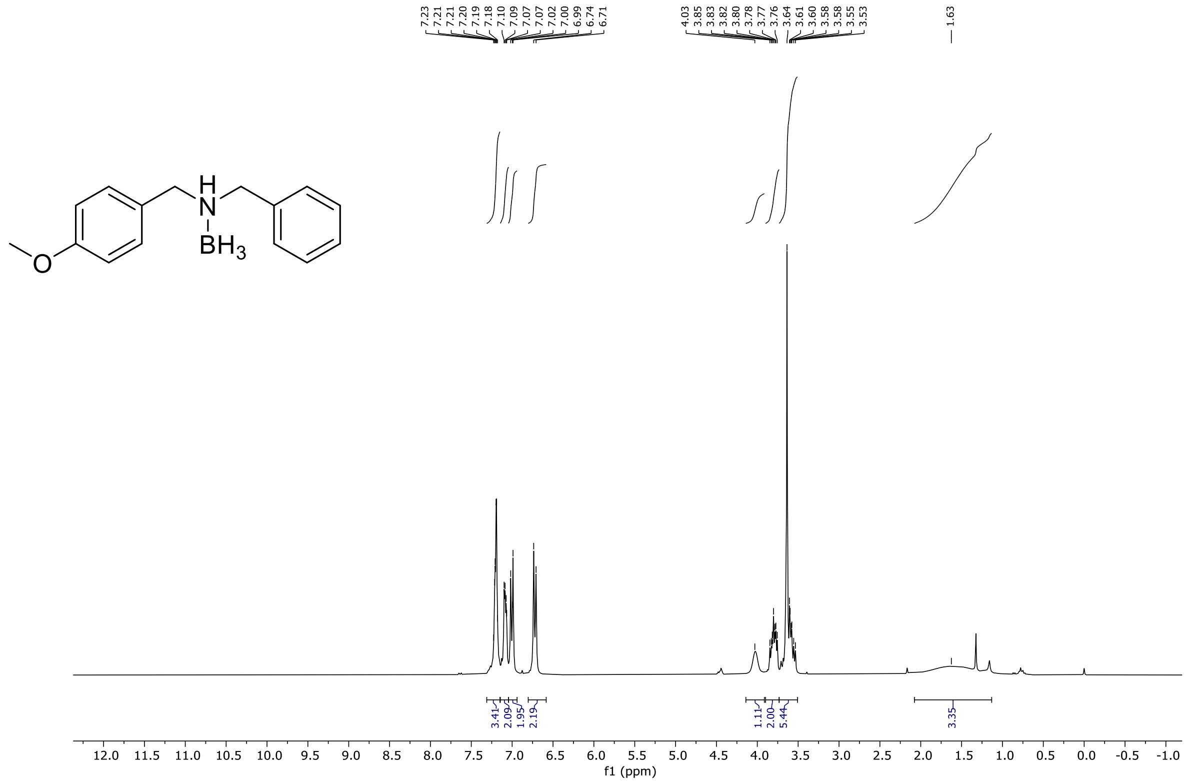

^{1}H NMR (300 MHz, $CDCl_3$) borane-*N*-benzyl-1-(4-methoxyphenyl)methanamine (**2c-BH$_3$**)

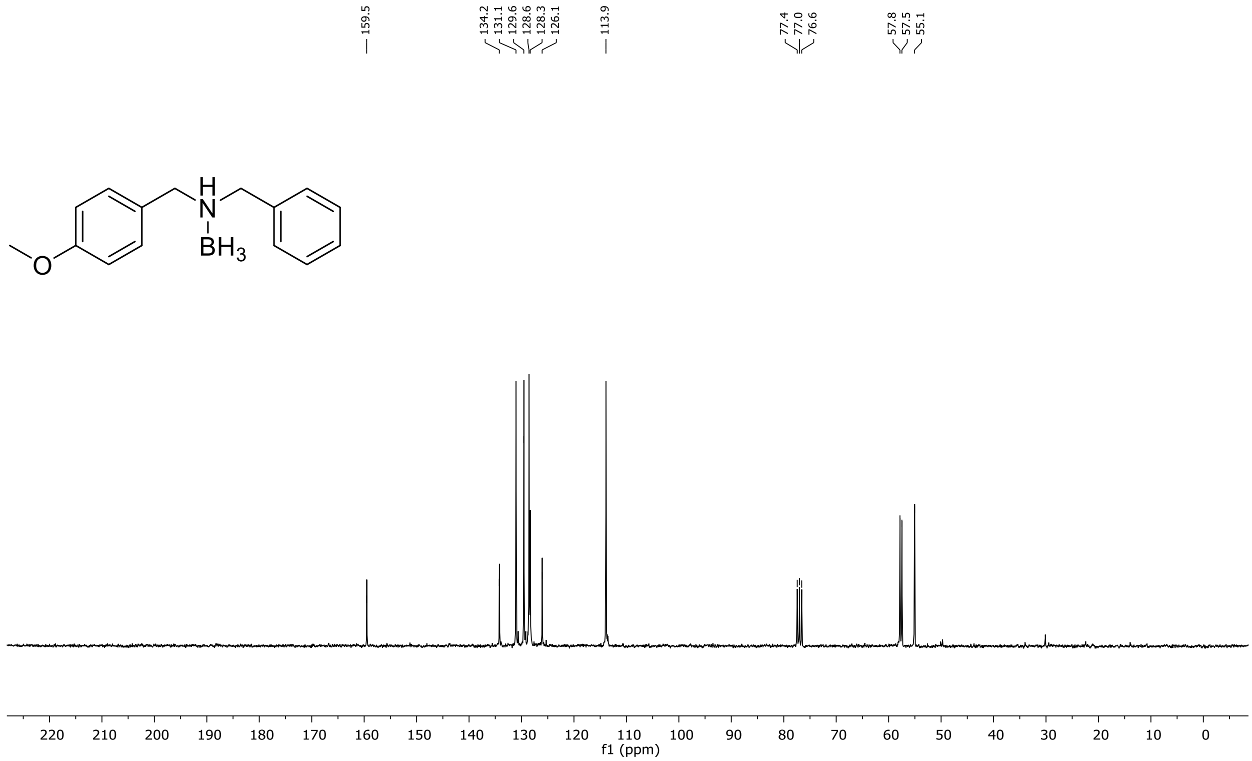

^{13}C {H} NMR (75 MHz, $CDCl_3$) borane-*N*-benzyl-1-(4-methoxyphenyl)methanamine (**2c-BH$_3$**)

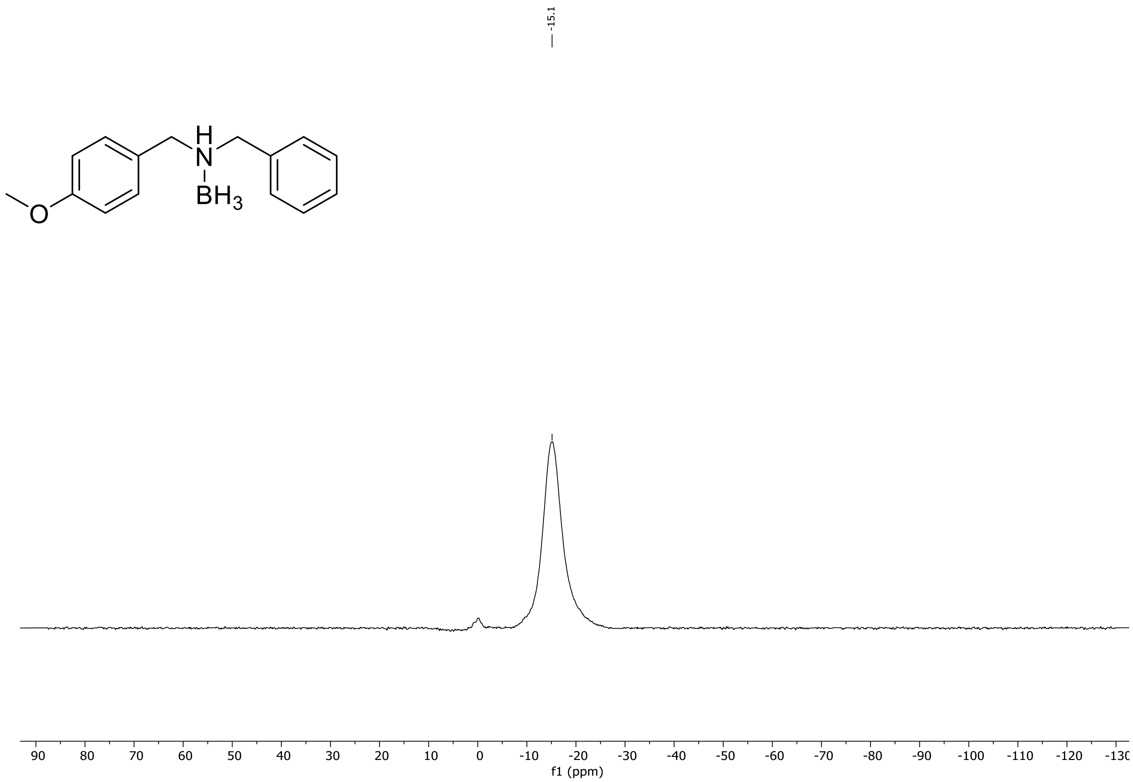

^{11}B NMR (96 MHz, $CDCl_3$) borane-*N*-benzyl-1-(4-methoxyphenyl)methanamine (**2c-BH$_3$**)

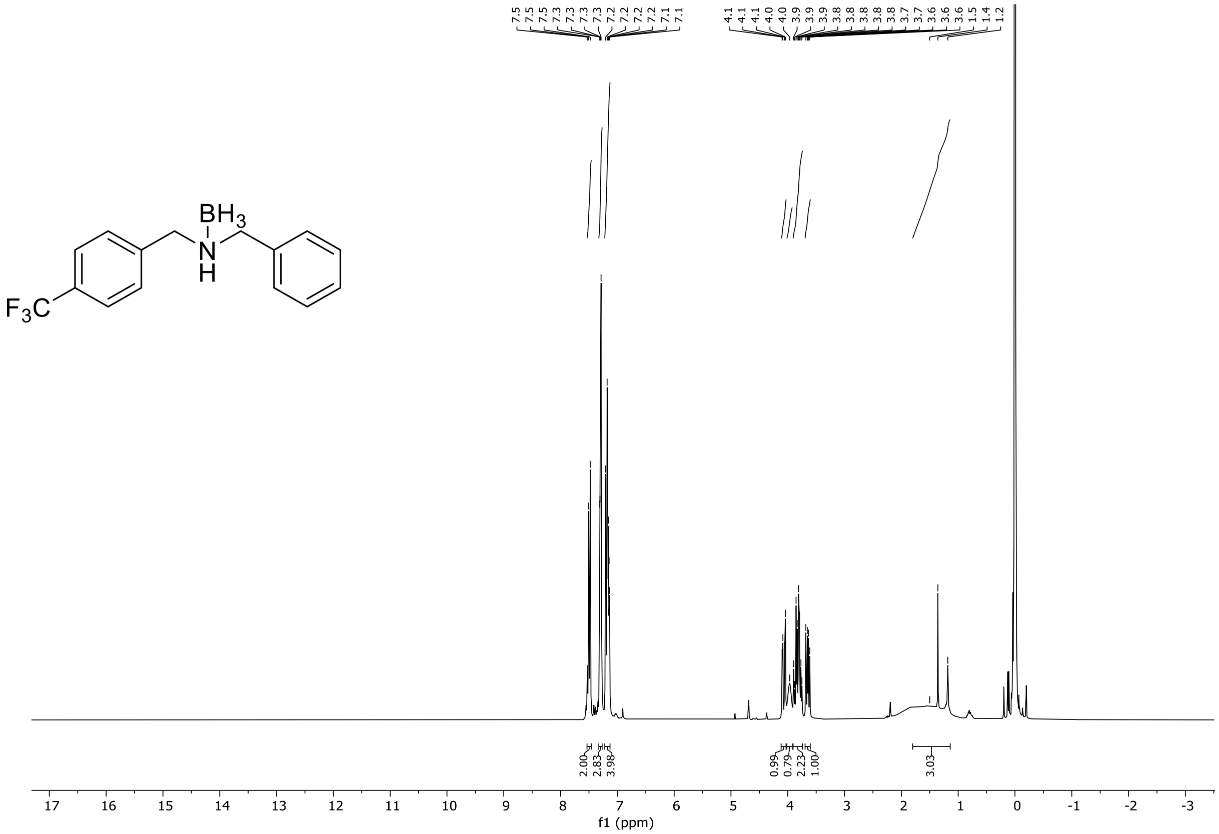

^{1}H NMR (300 MHz, $CDCl_3$) borane-*N*-benzyl-1-(4-(trifluoromethyl)phenyl)methanamine (**2d-BH$_3$**)

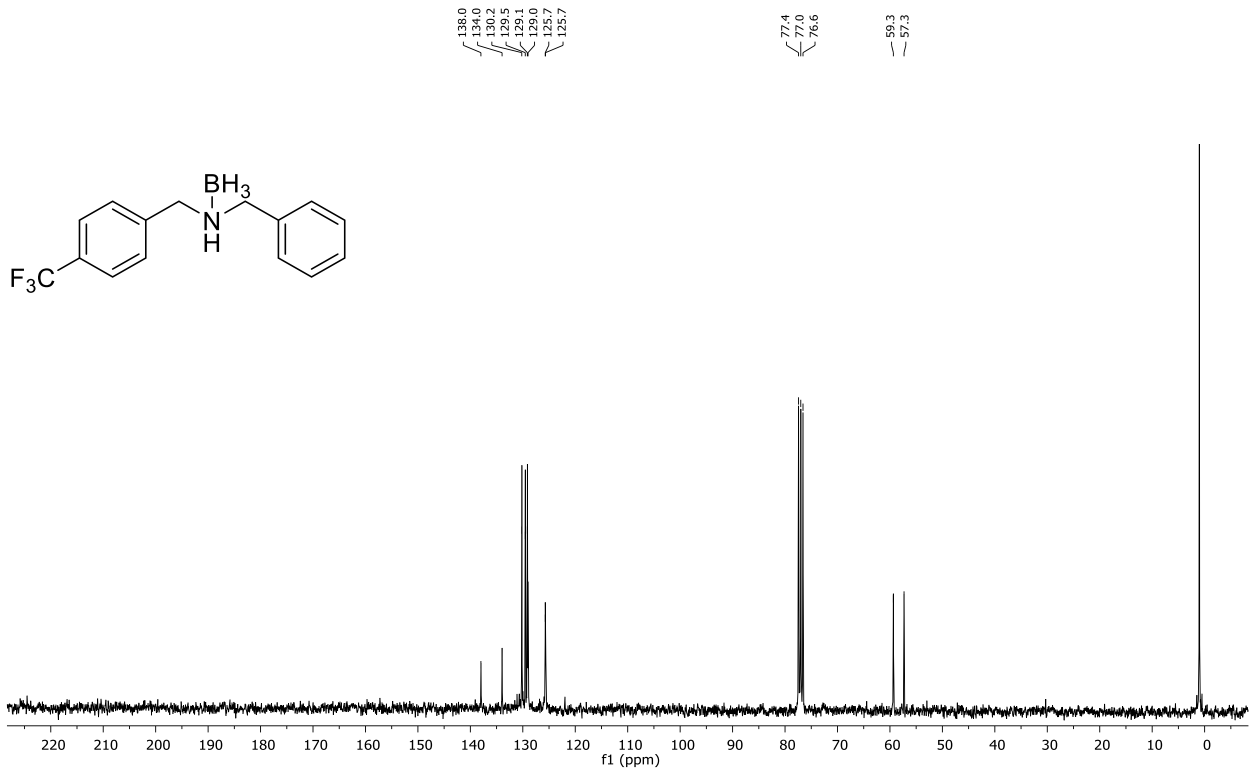

^{13}C {H} NMR (75 MHz, $CDCl_3$) borane-*N*-benzyl-1-(4-(trifluoromethyl)phenyl)methanamine (**2d-BH$_3$**)

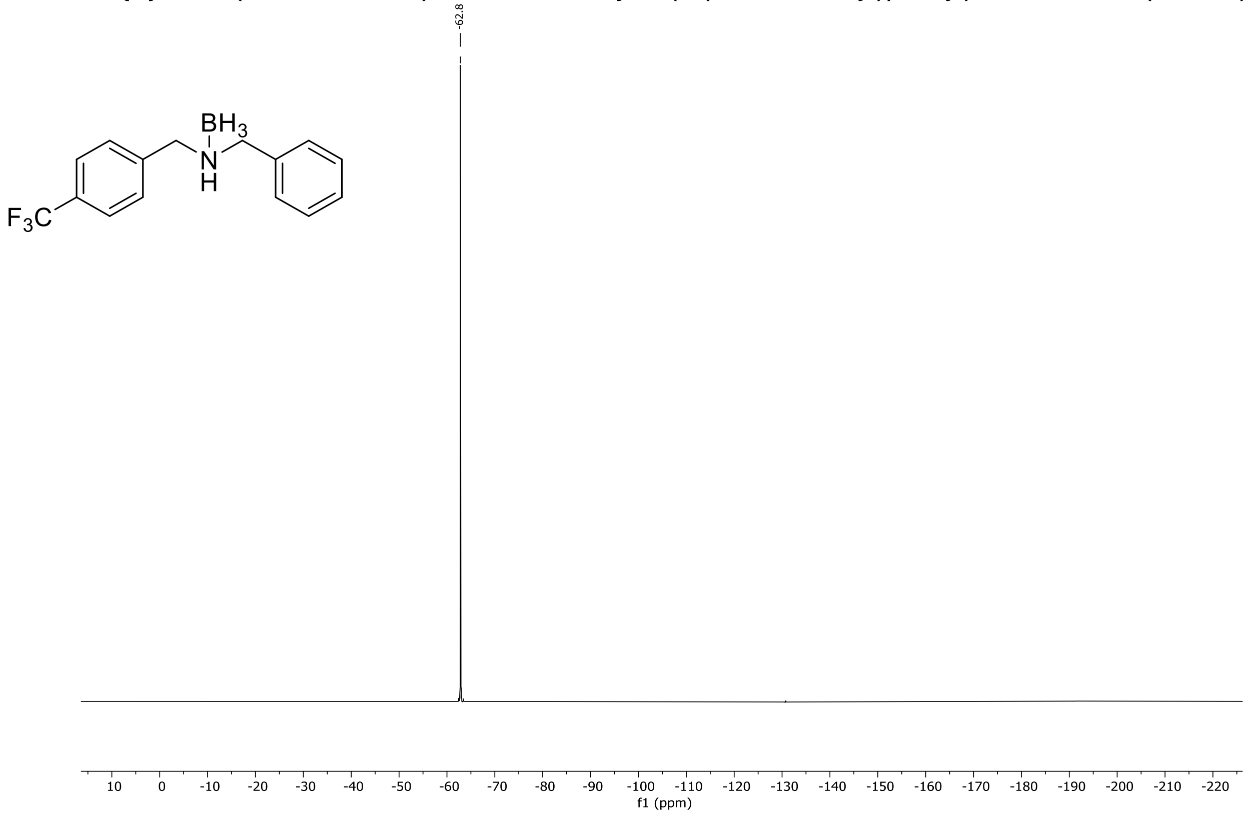

^{19}F NMR (282 MHz, $CDCl_3$) borane-*N*-benzyl-1-(4-(trifluoromethyl)phenyl)methanamine (**2d-BH$_3$**)

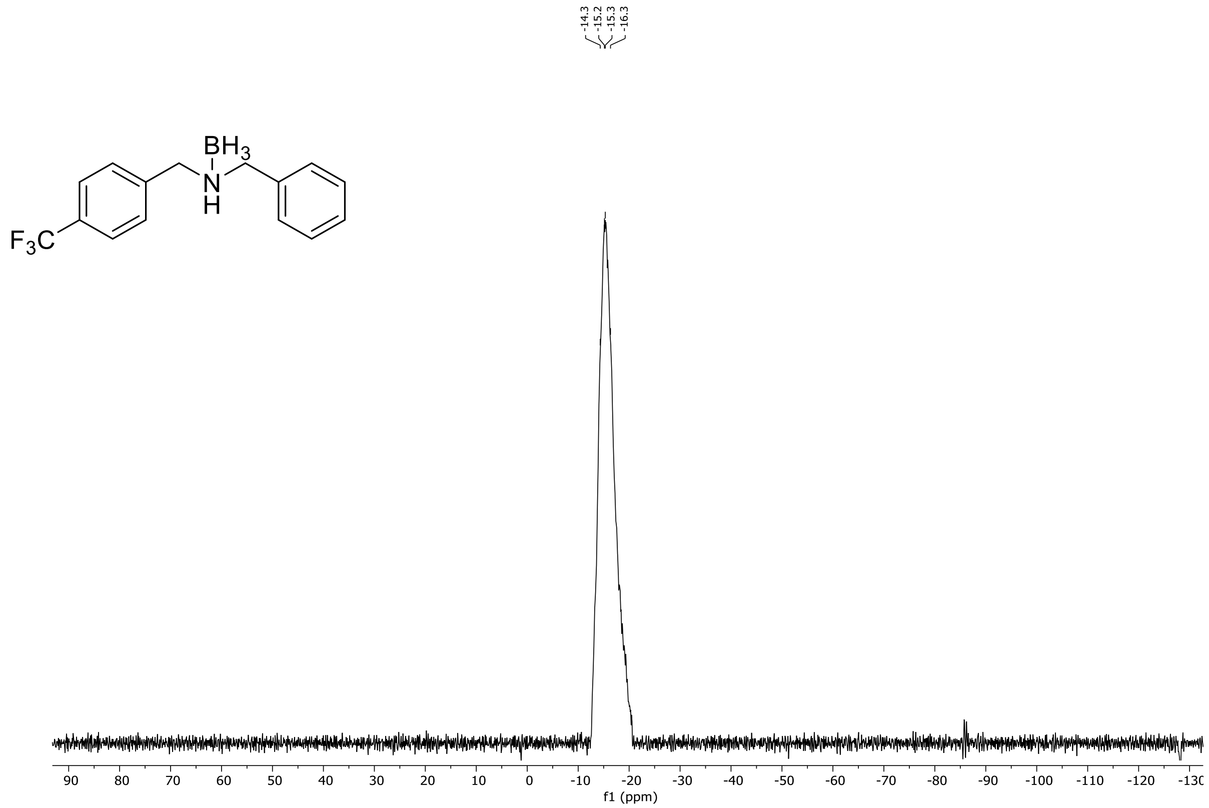

^{11}B NMR (96 MHz, $CDCl_3$) borane-*N*-benzyl-1-(4-(trifluoromethyl)phenyl)methanamine (**2d-BH$_3$**)

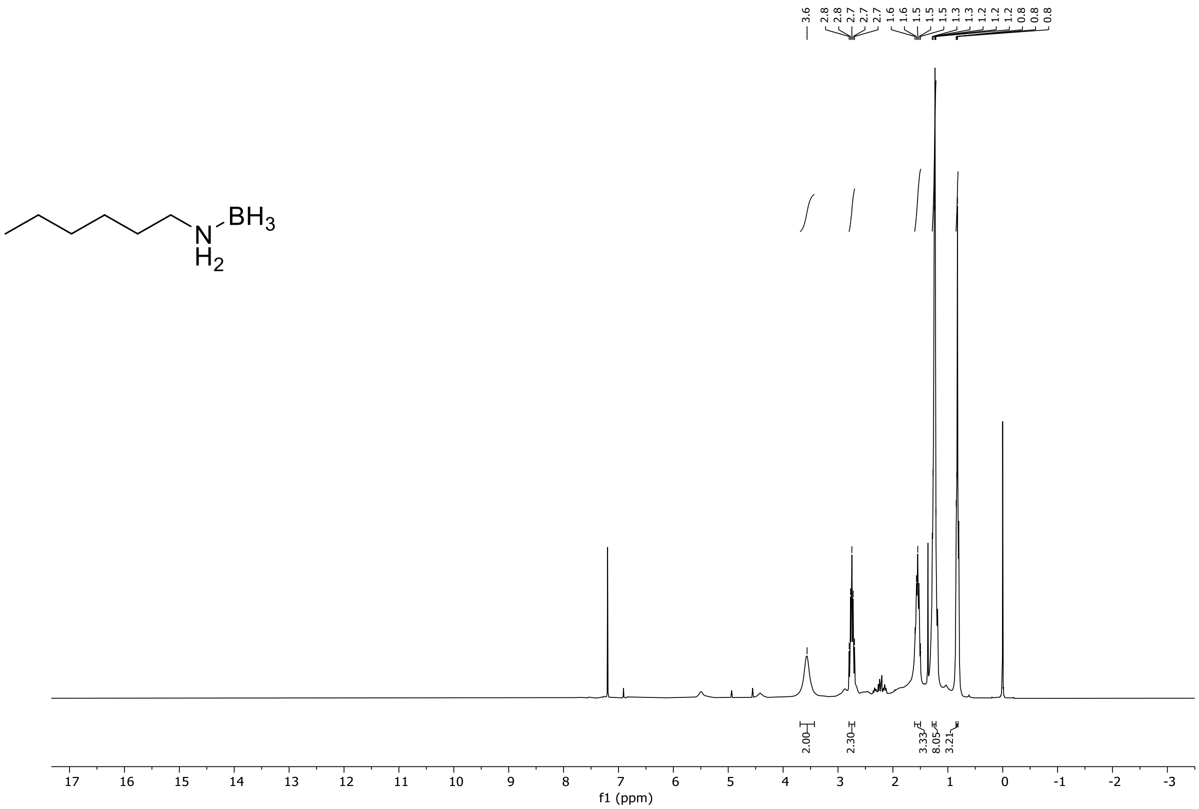

^{1}H NMR (300 MHz, $CDCl_3$) borane-hexylamine (**2e-BH$_3$**)

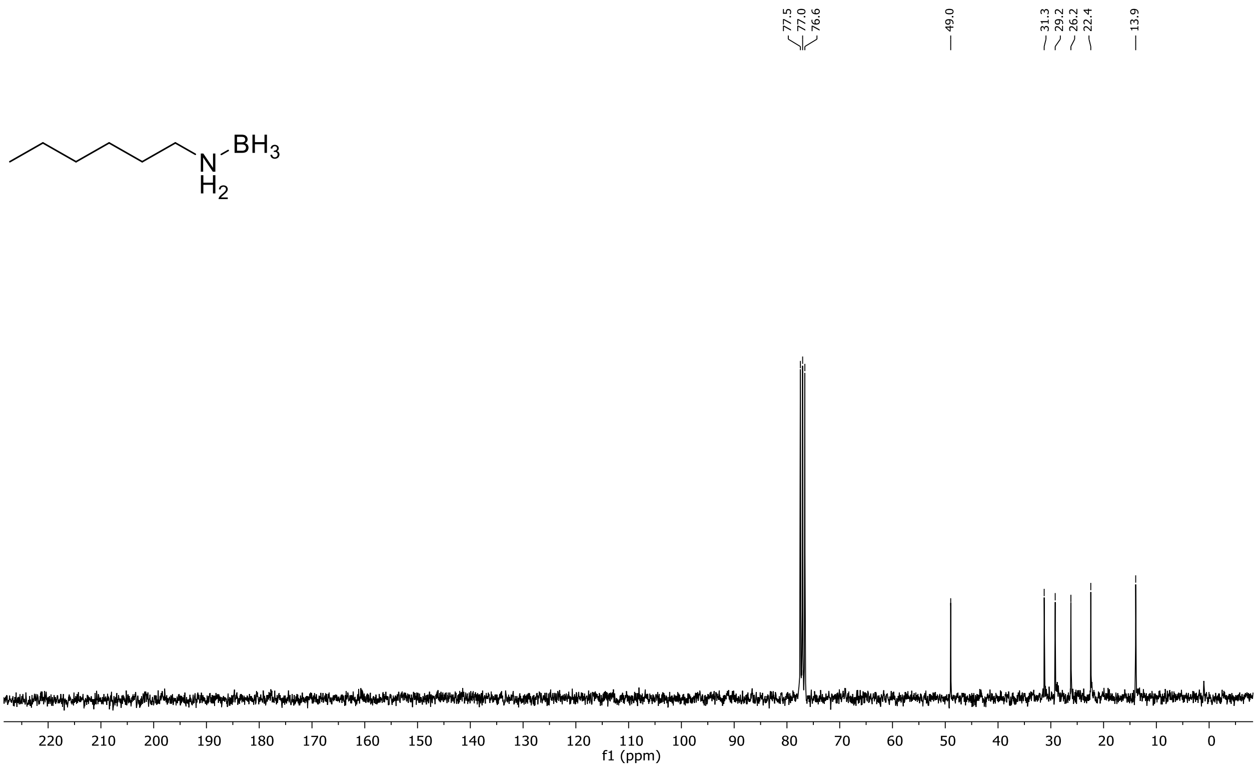

^{13}C {H} NMR (75 MHz, $CDCl_3$) borane-hexylamine (**2e-BH$_3$**)

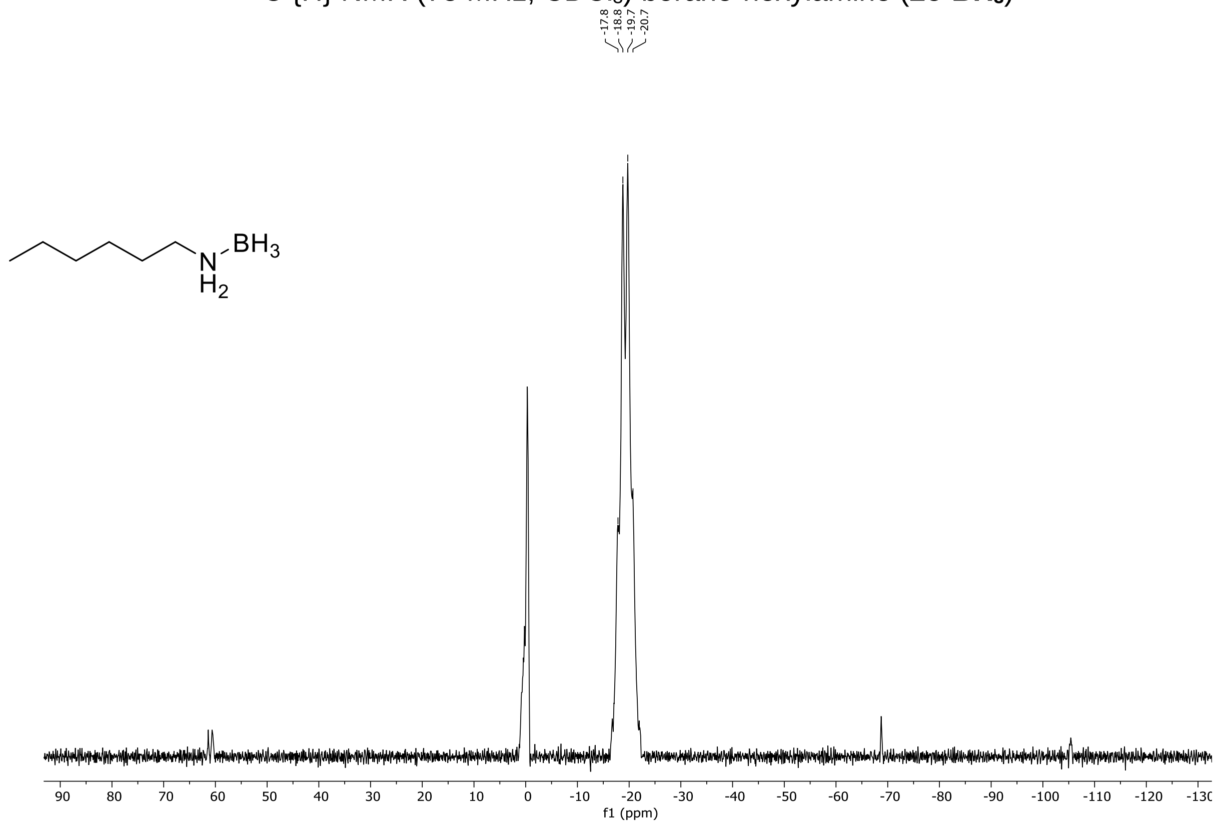

^{11}B NMR (96 MHz, $CDCl_3$) borane-hexylamine (**2e-BH$_3$**)

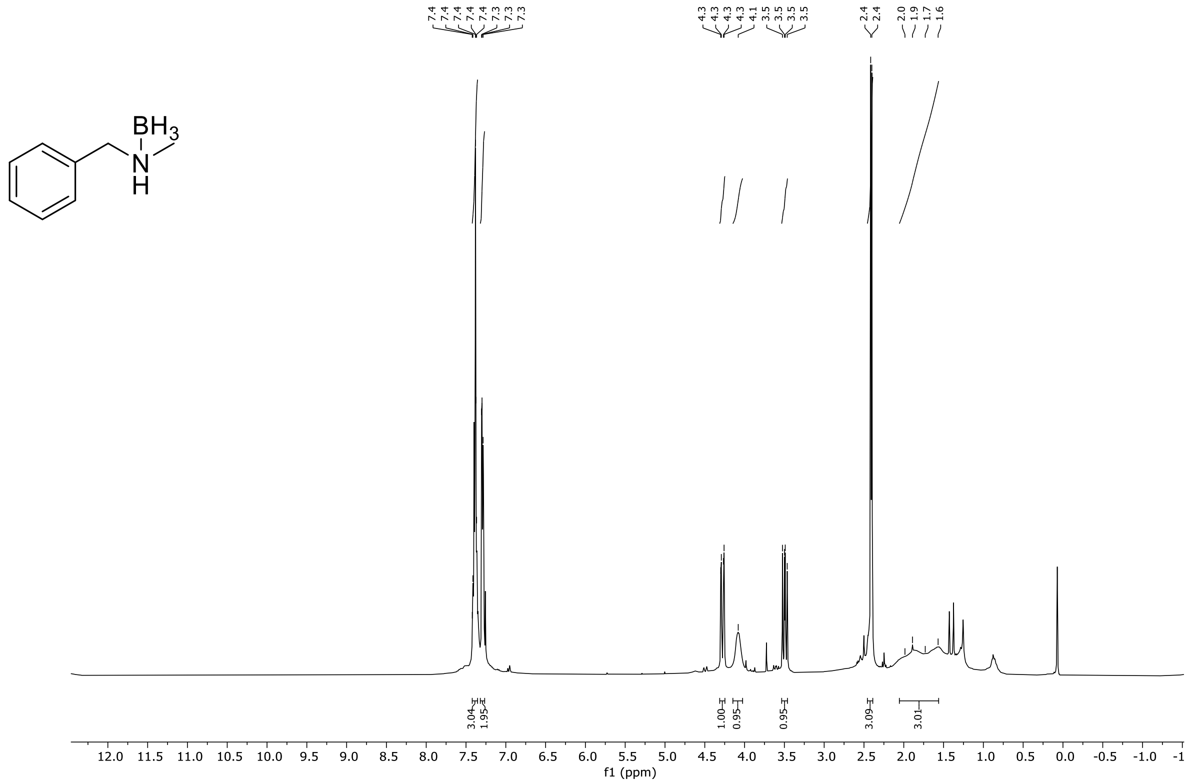

^{1}H NMR (400 MHz, $CDCl_3$) borane-*N*-methyl-1-phenylmethanamine (**2f-BH$_3$**)

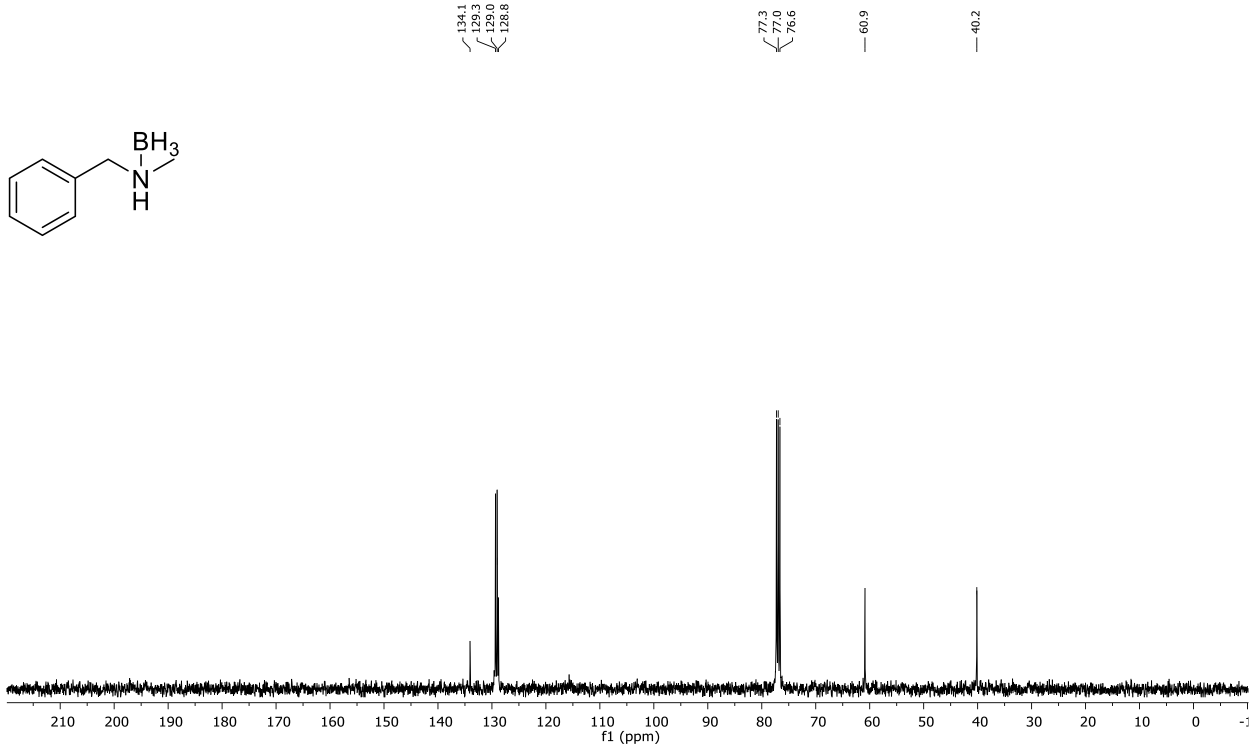

^{13}C {H} NMR (101 MHz, $CDCl_3$) borane-*N*-methyl-1-phenylmethanamine (**2f-BH$_3$**)

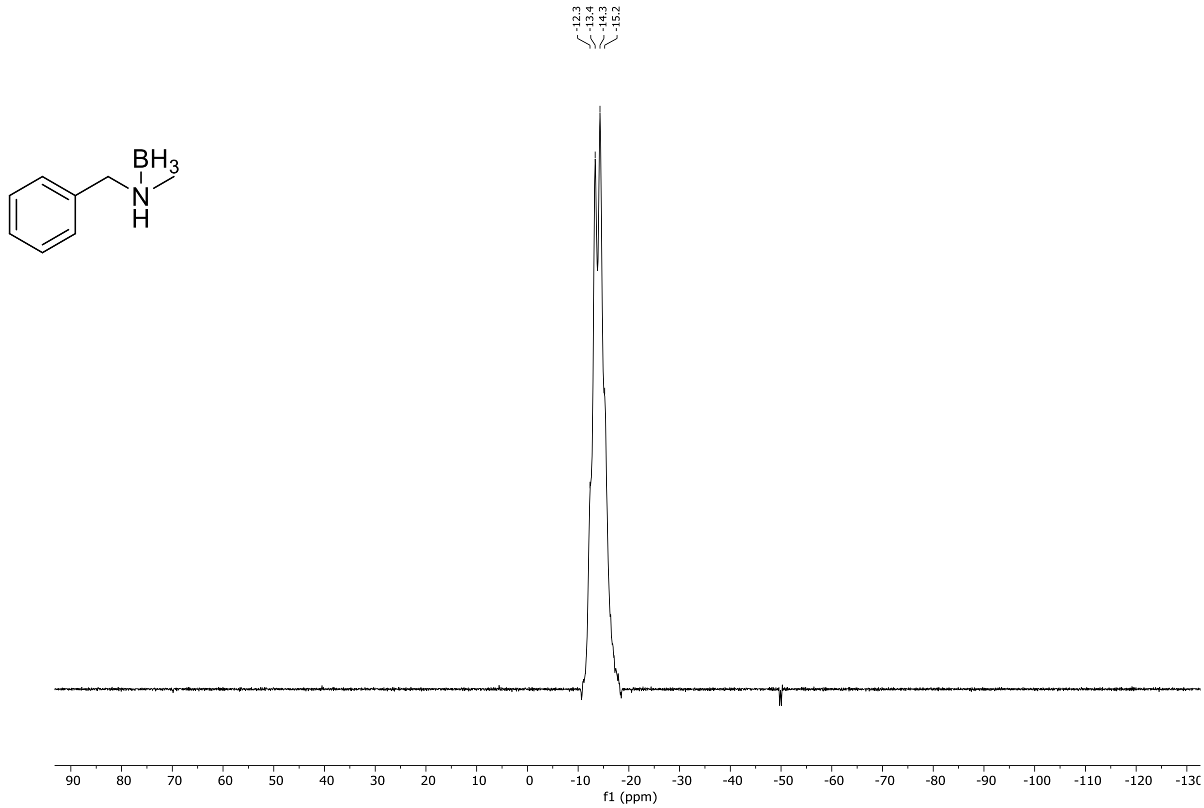

^{11}B NMR (96 MHz, $CDCl_3$) borane-*N*-methyl-1-phenylmethanamine (**2f-BH₃**)

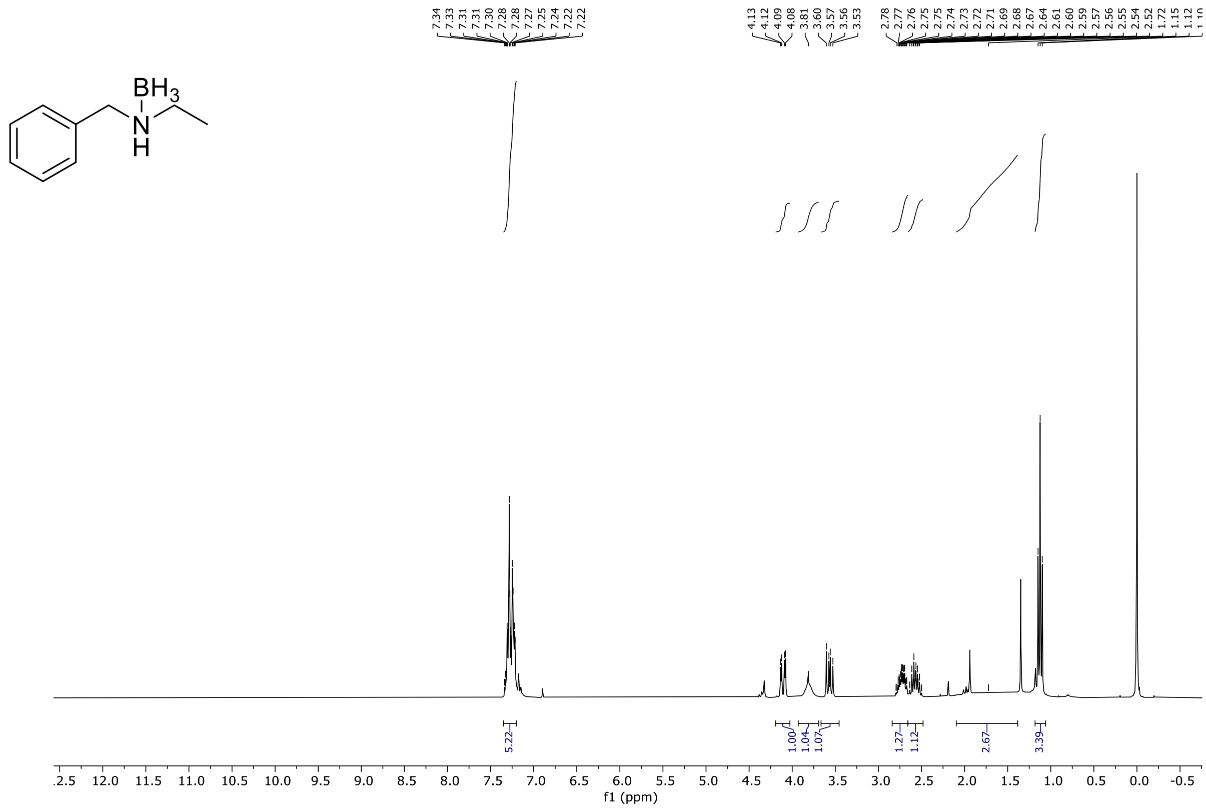

^{1}H NMR (300 MHz, $CDCl_3$) borane-*N*-benzylethanamine (**2g-BH₃**)

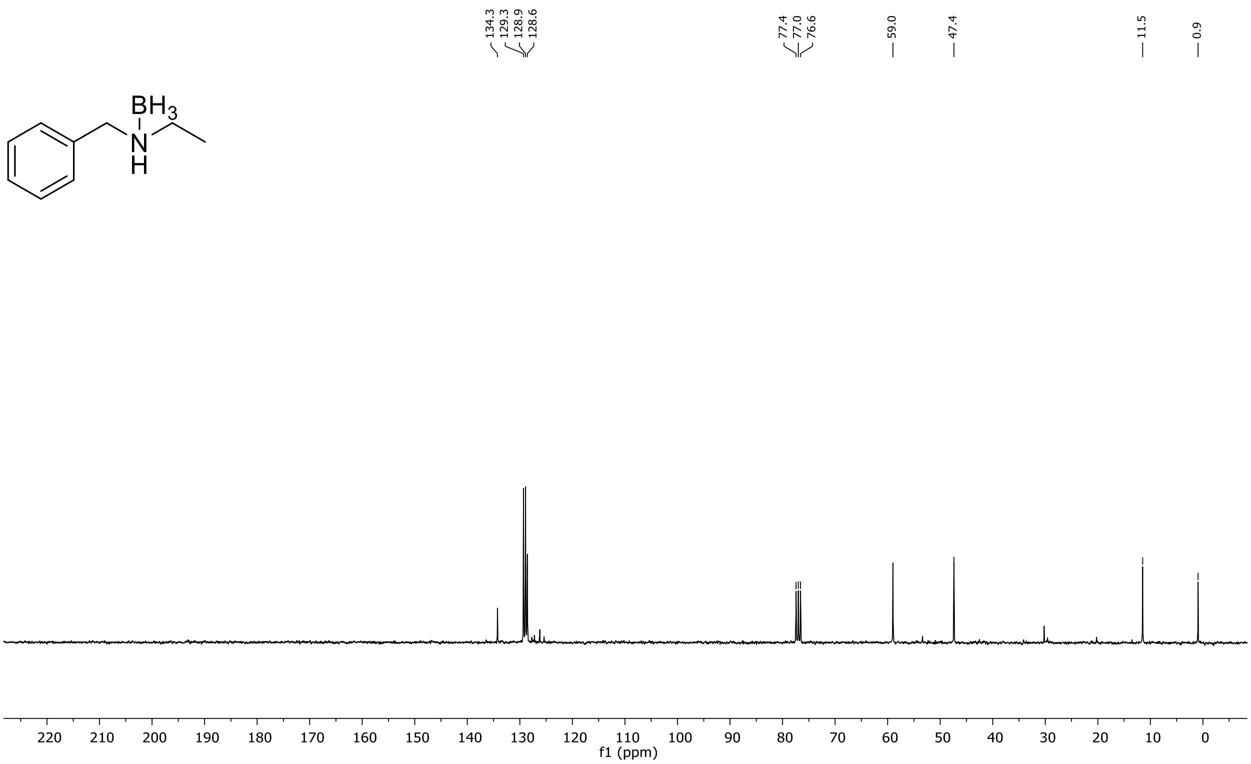

^{13}C {H} NMR (75 MHz, $CDCl_3$) borane-*N*-benzylethanamine (**2g-BH$_3$**)

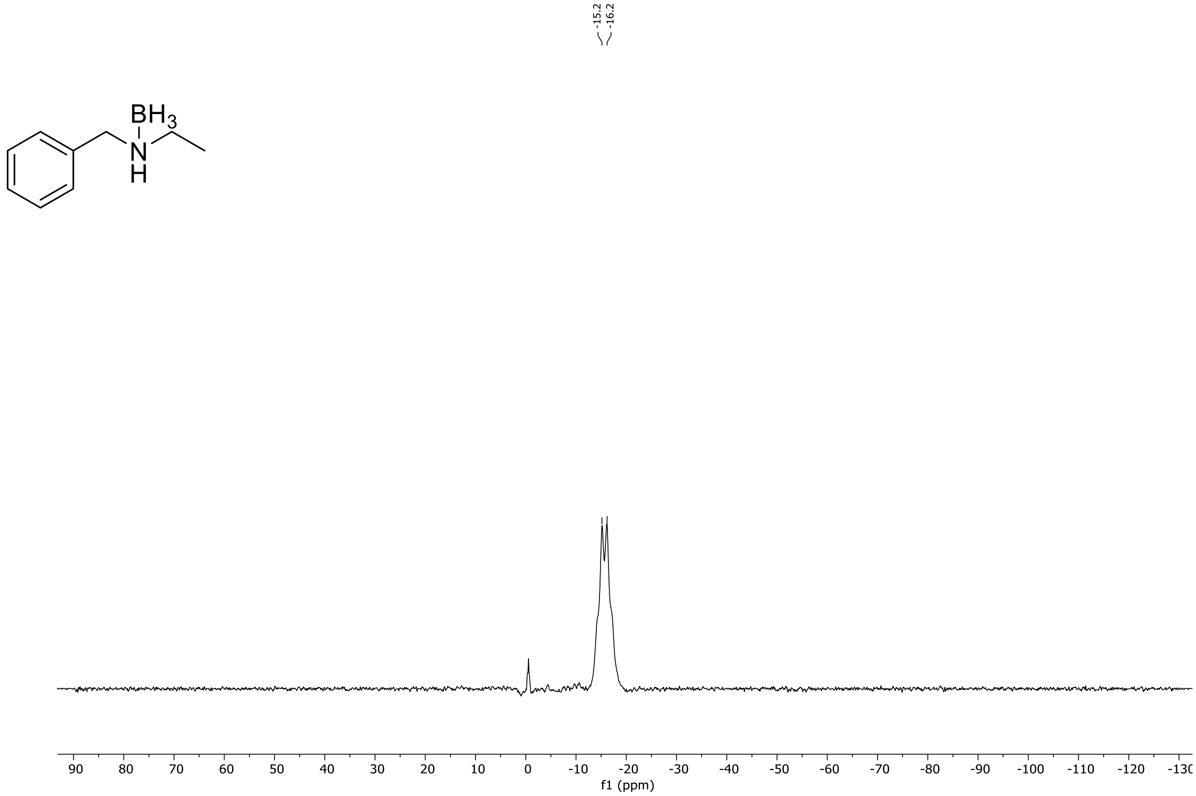

^{11}B NMR (96 MHz, $CDCl_3$) borane-*N*-benzylethanamine (**2g-BH$_3$**)

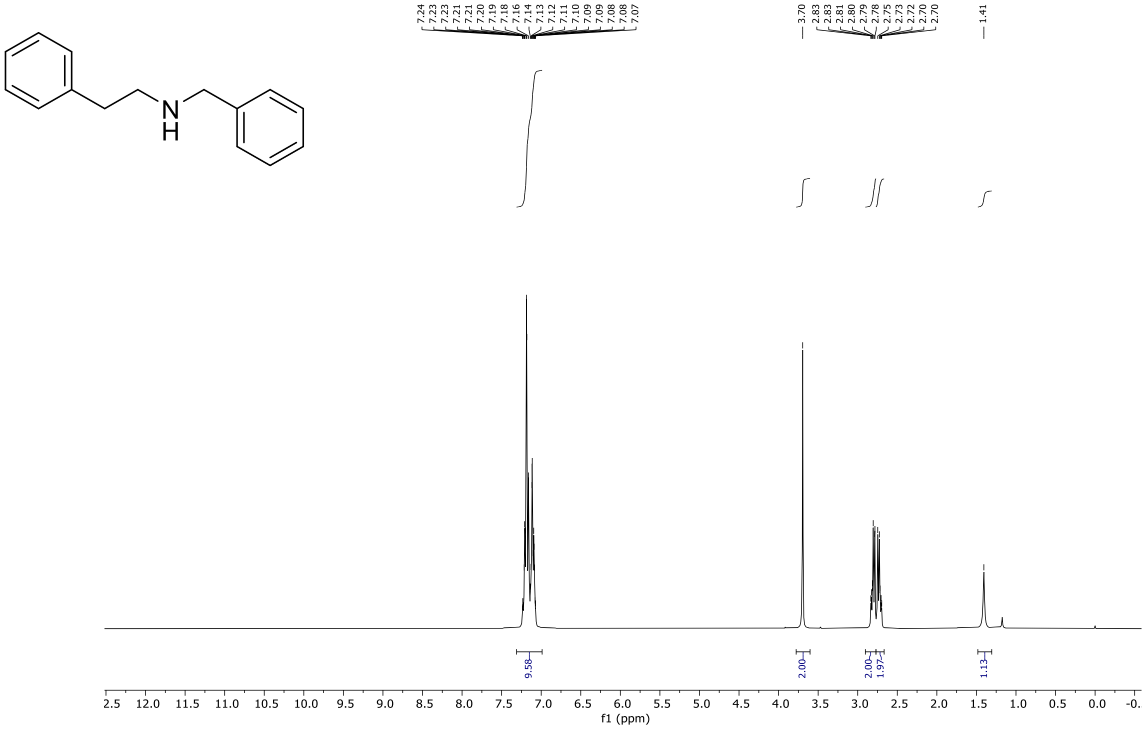

^{1}H NMR (300 MHz, $CDCl_3$) *N*-benzyl-2-phenylethan-1-amine (**2h**)

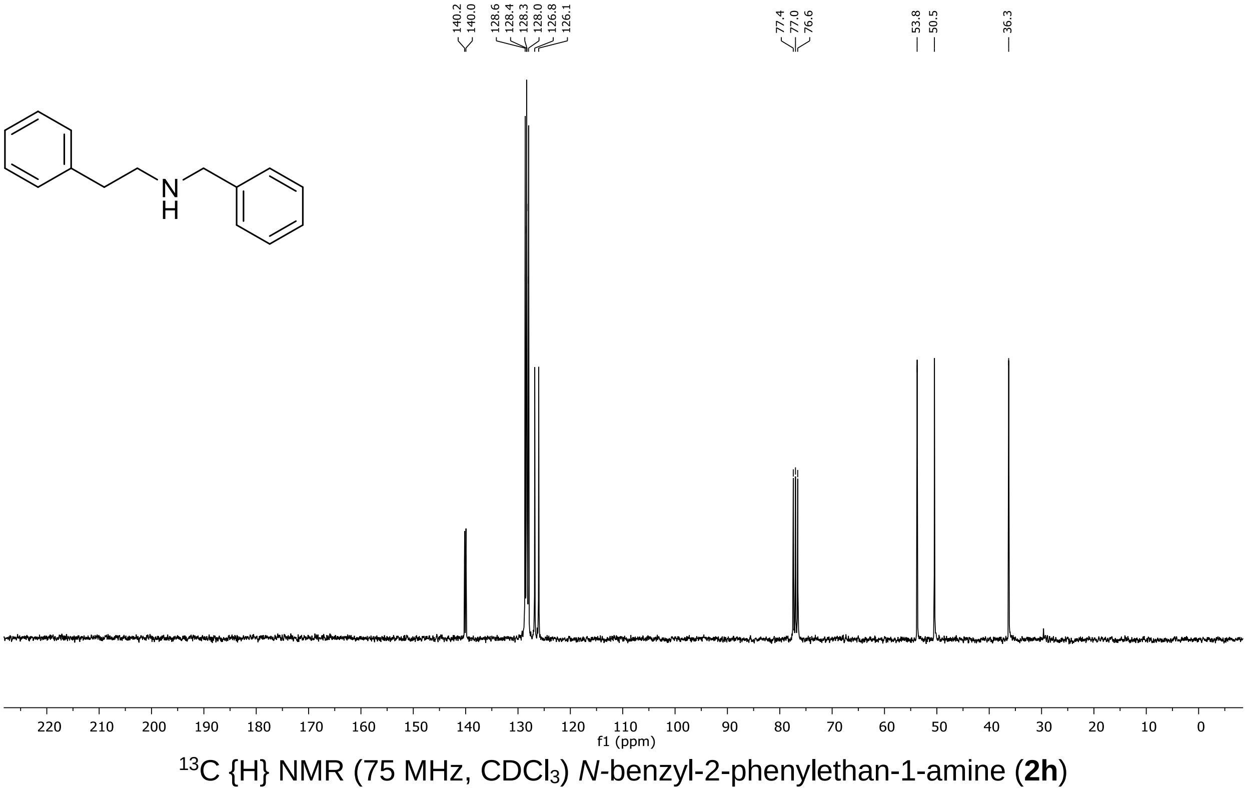

^{13}C {H} NMR (75 MHz, $CDCl_3$) *N*-benzyl-2-phenylethan-1-amine (**2h**)

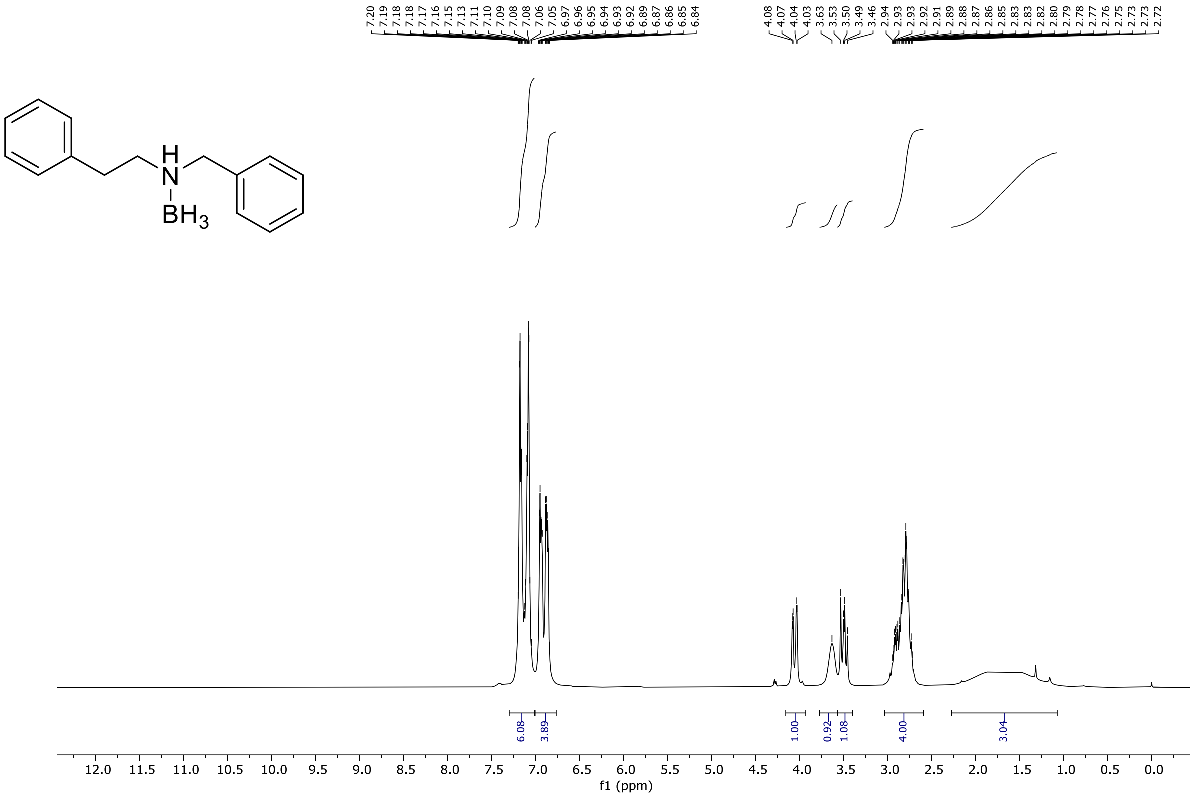

^{1}H NMR (300 MHz, CDCl$_3$) borane-*N*-benzyl-2-phenylethan-1-amine (**2h-BH$_3$**)

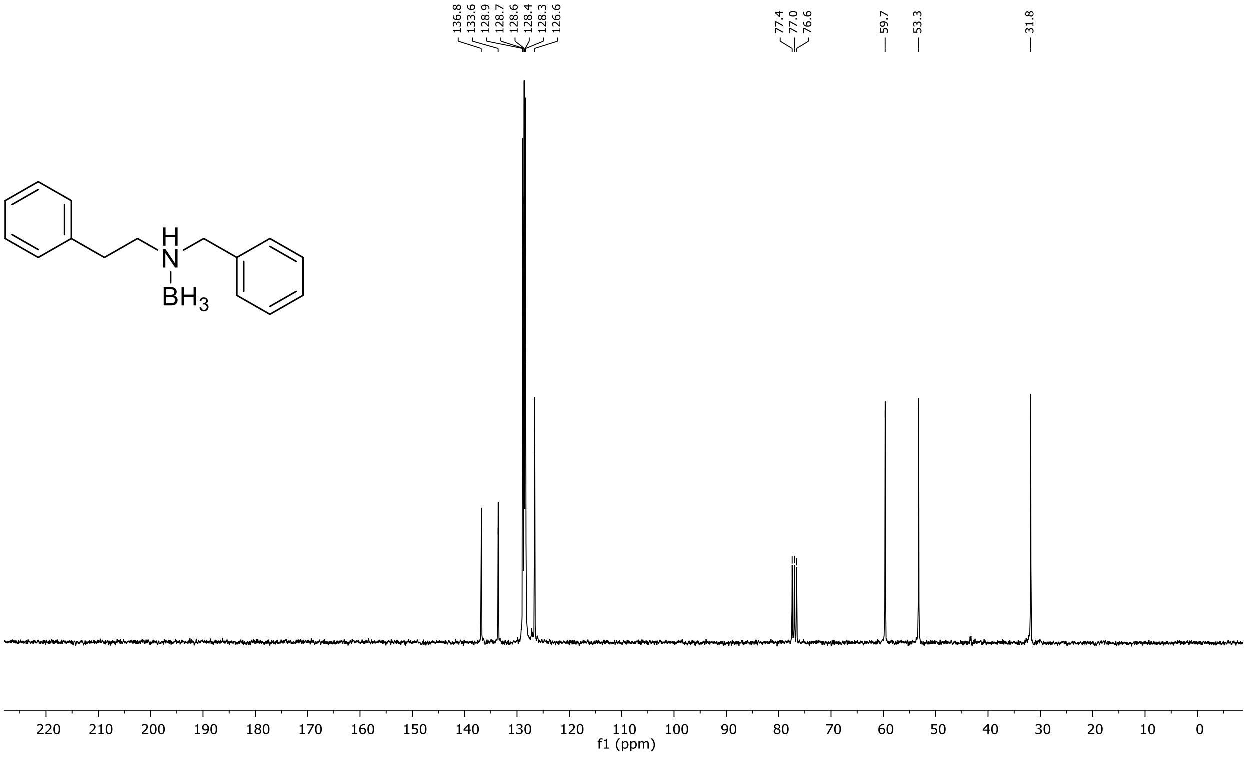

^{13}C {H} NMR (75 MHz, CDCl$_3$) borane-*N*-benzyl-2-phenylethan-1-amine (**2h-BH$_3$**)

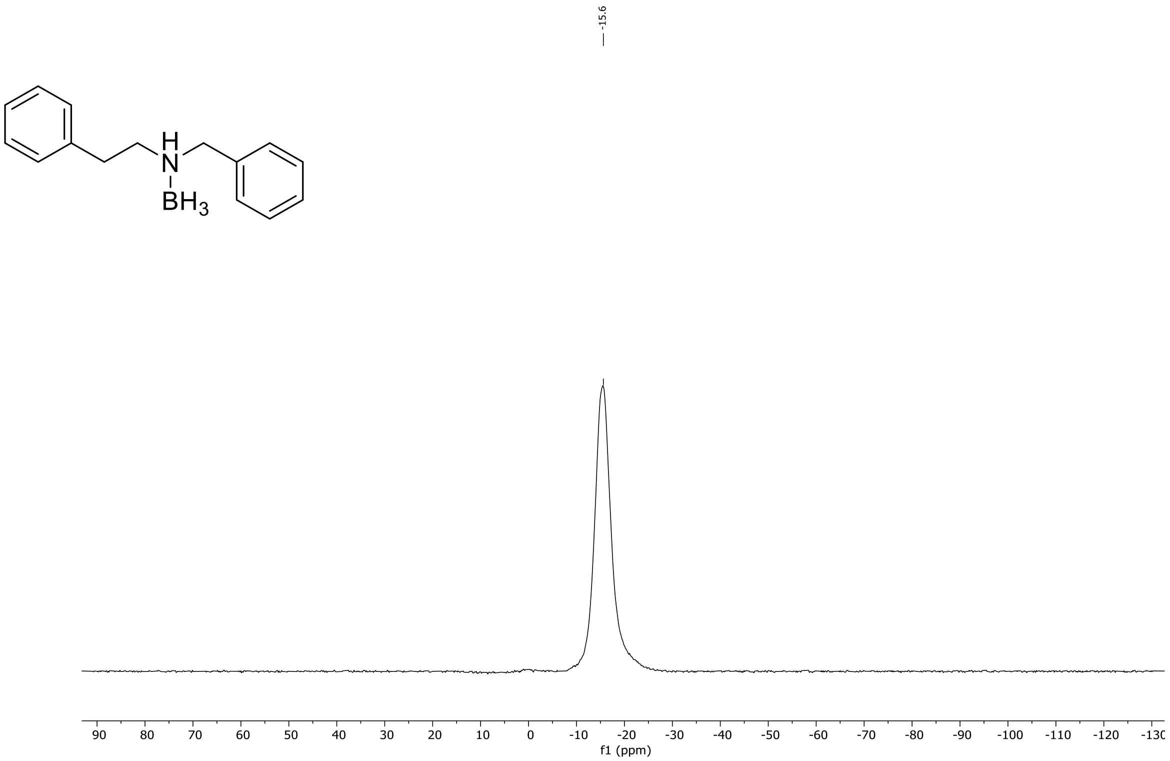

^{11}B NMR (96 MHz, $CDCl_3$) borane-*N*-benzyl-2-phenylethan-1-amine (**2h-BH$_3$**)

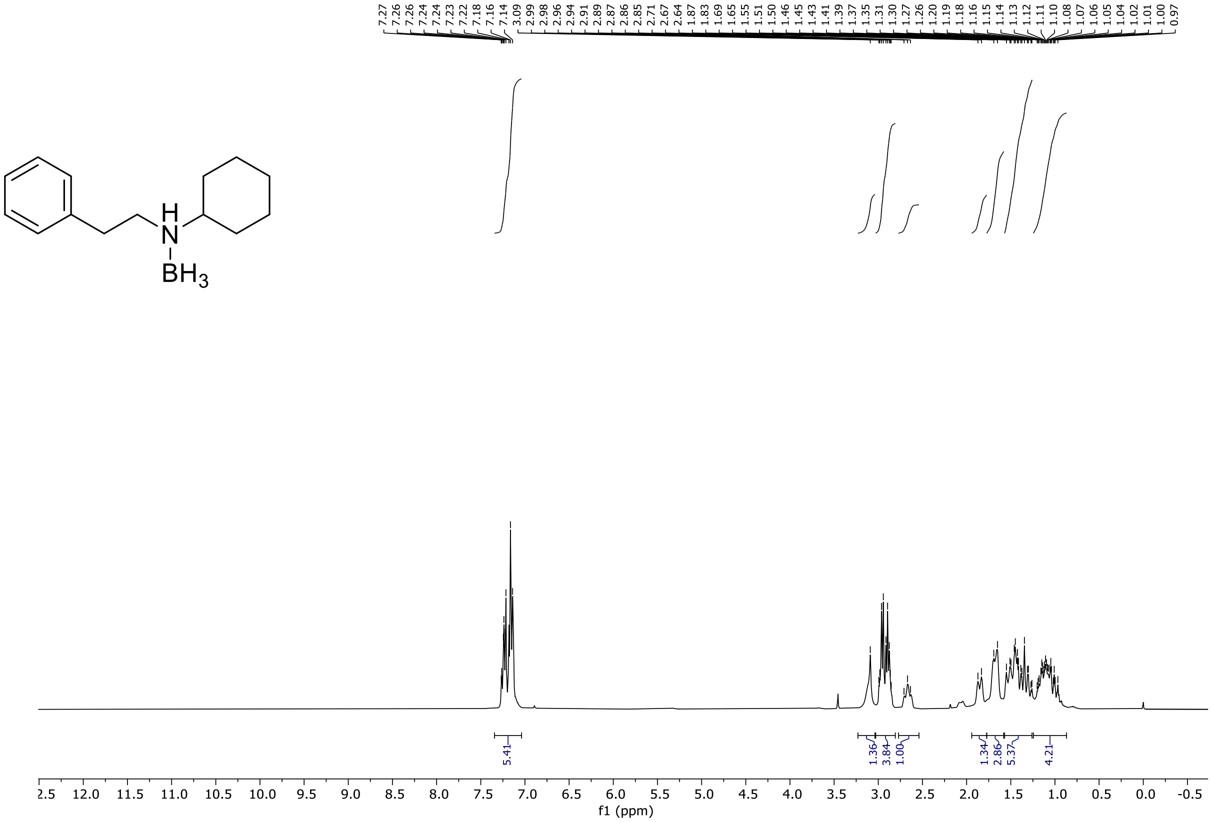

^{1}H NMR (300 MHz, $CDCl_3$) borane-*N*-phenethylcyclohexanamine (**2i-BH$_3$**)

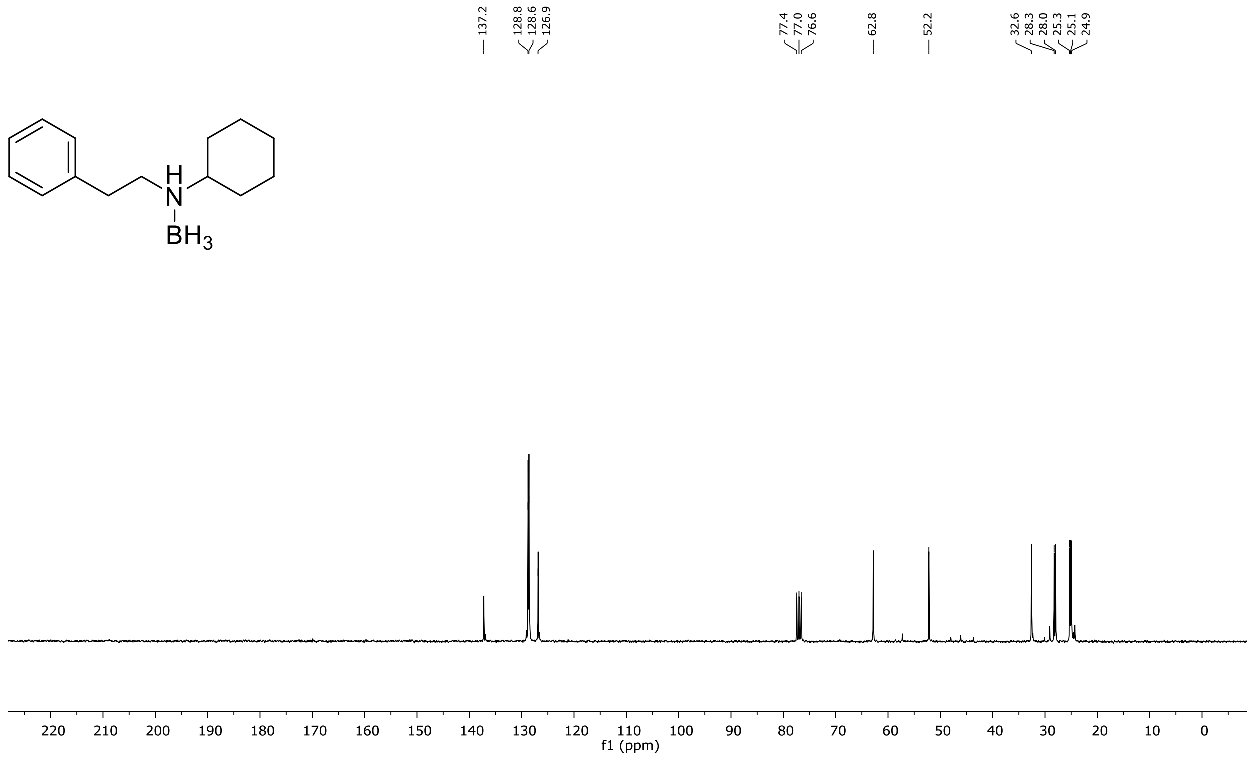

^{13}C {H} NMR (75 MHz, $CDCl_3$) borane-*N*-phenethylcyclohexanamine (**2i-BH$_3$**)

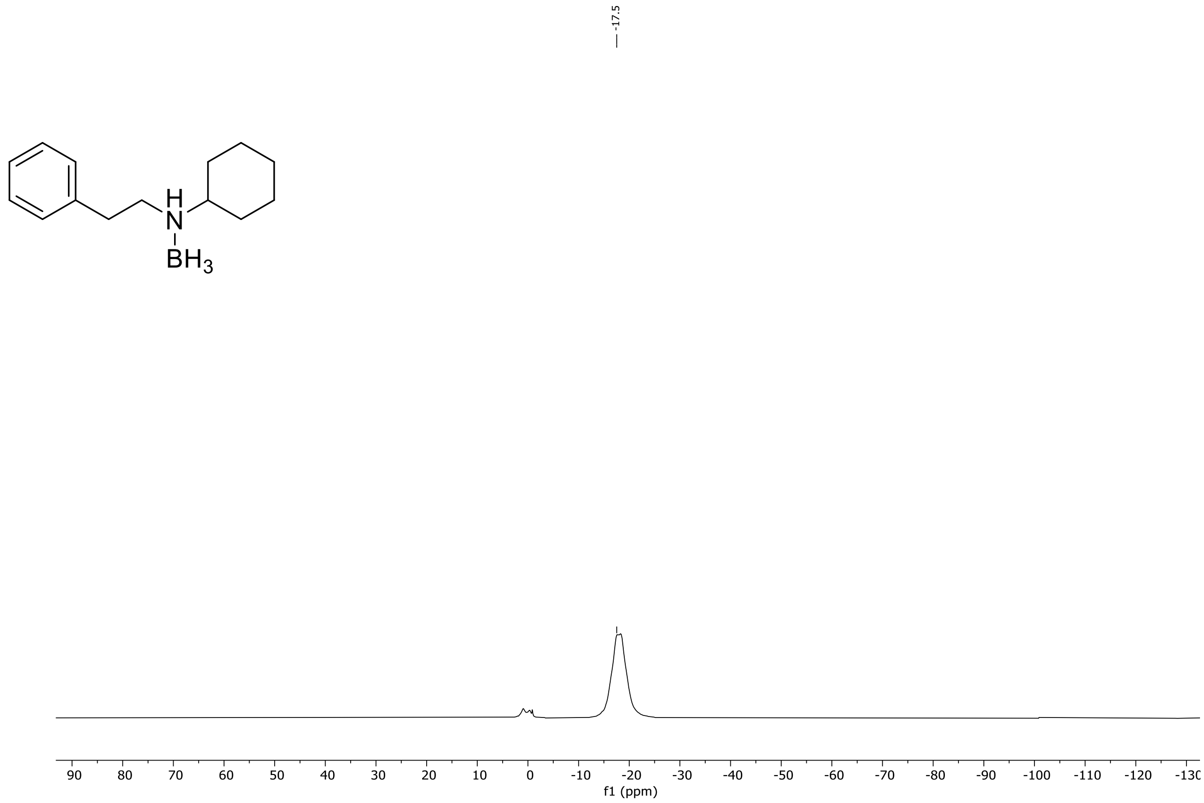

^{11}B NMR (96 MHz, $CDCl_3$) borane-*N*-phenethylcyclohexanamine (**2i-BH$_3$**)

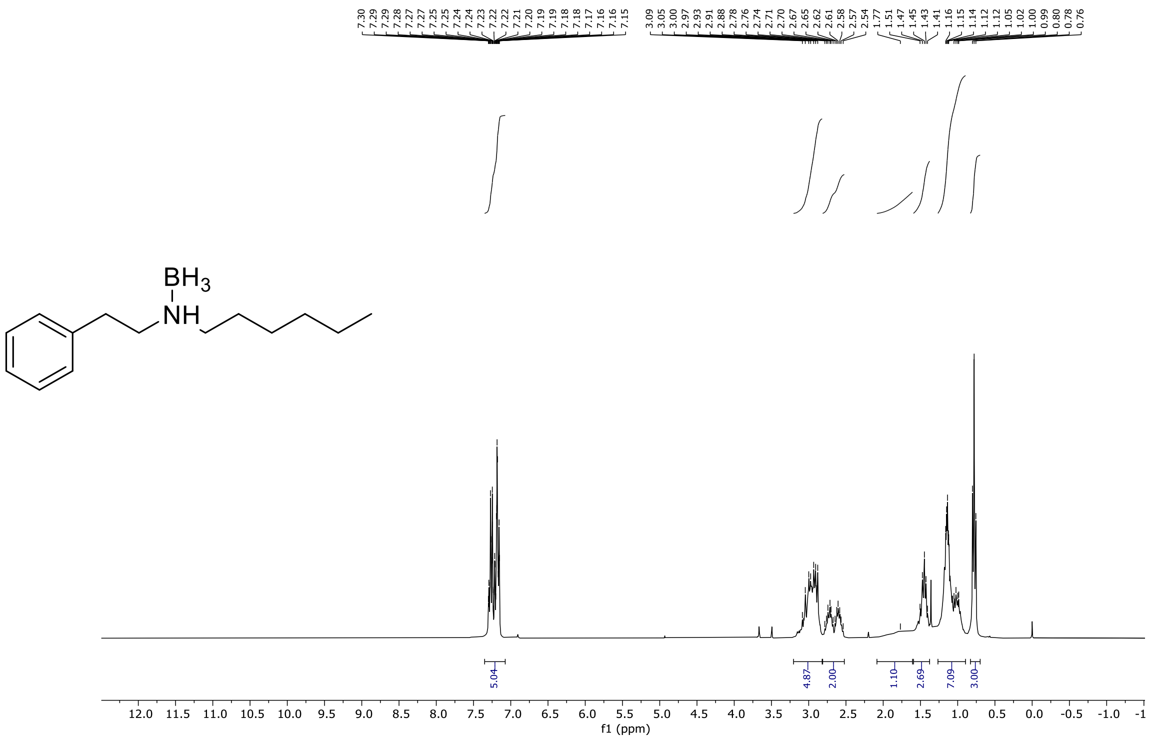

^{1}H NMR (300 MHz, $CDCl_3$) borane-*N*-phenethylhexan-1-amine (**2j-BH$_3$**)

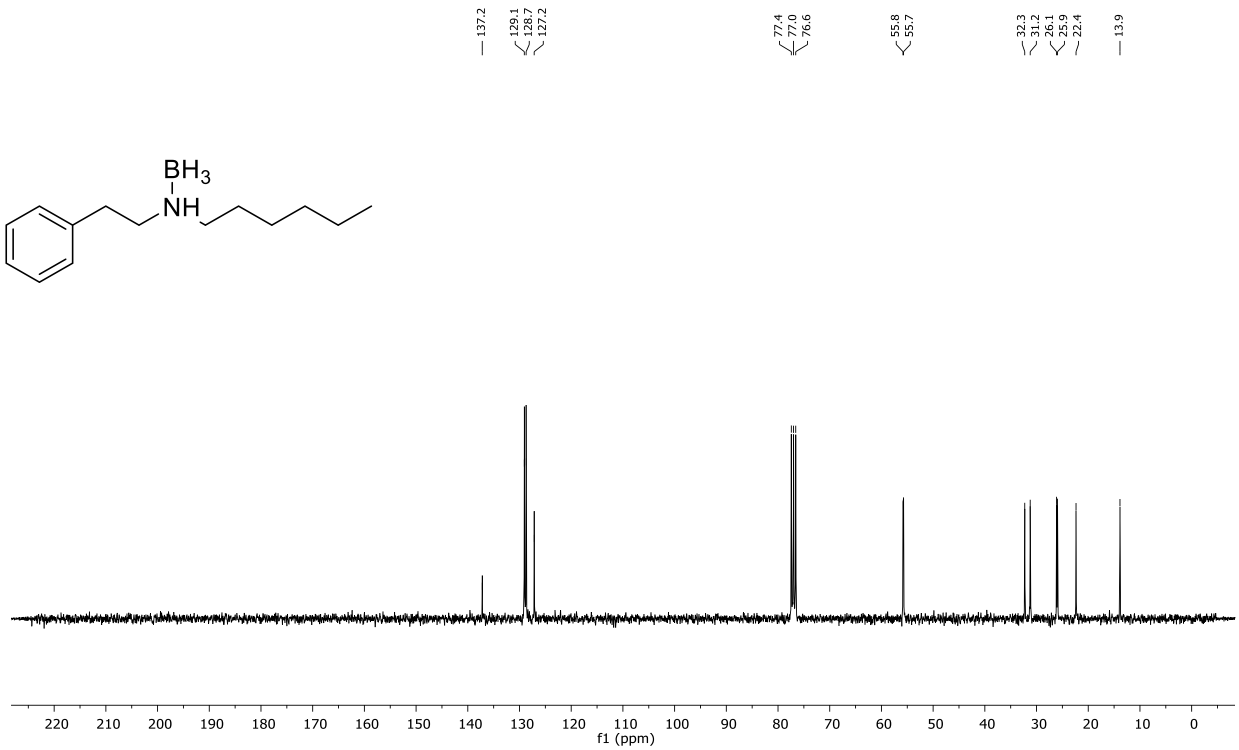

^{13}C {H} NMR (75 MHz, $CDCl_3$) borane-*N*-phenethylhexan-1-amine (**2j-BH$_3$**)

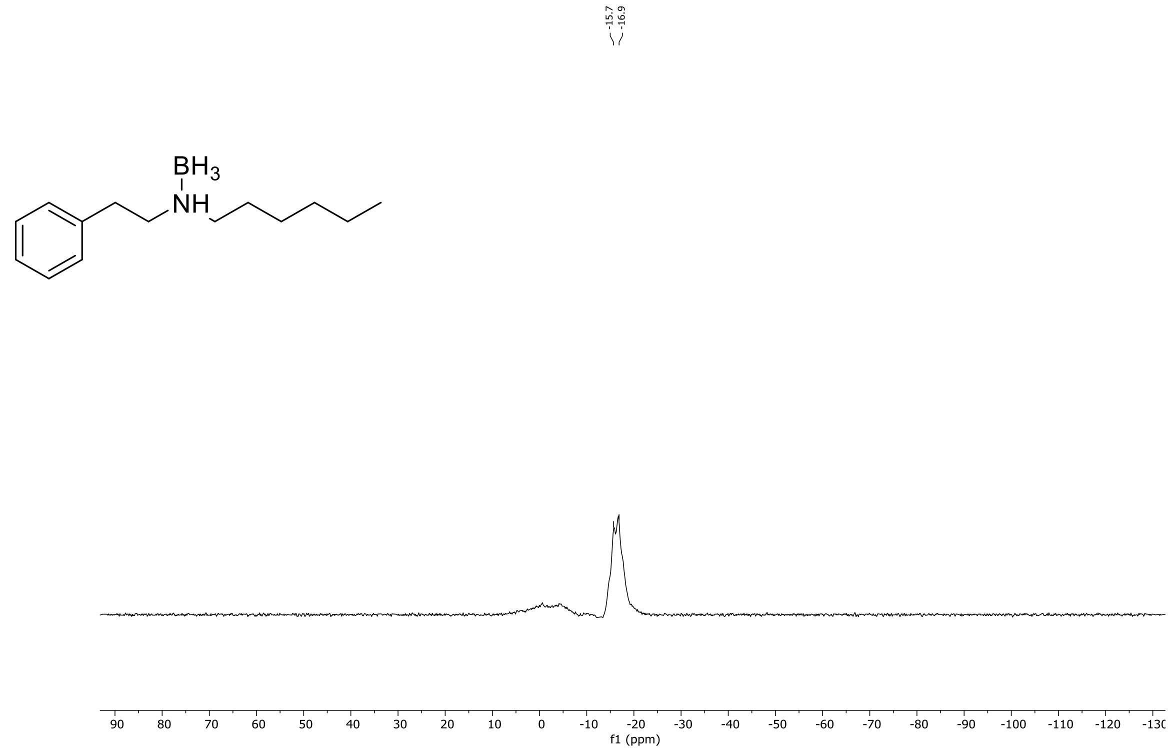

^{11}B NMR (96 MHz, $CDCl_3$) borane-*N*-phenethylhexan-1-amine (**2j-BH$_3$**)

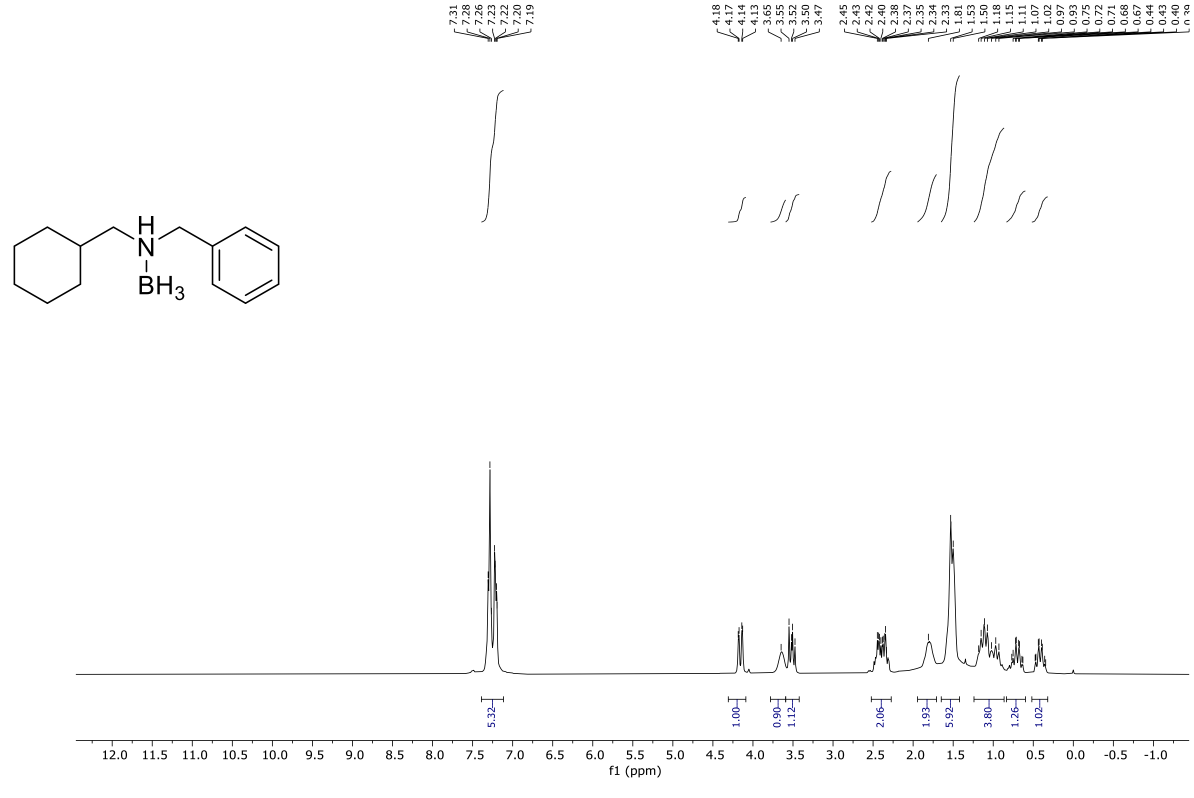

^{1}H NMR (300 MHz, $CDCl_3$) borane-*N*-benzyl-1-cyclohexylmethanamine (**2k-BH$_3$**)

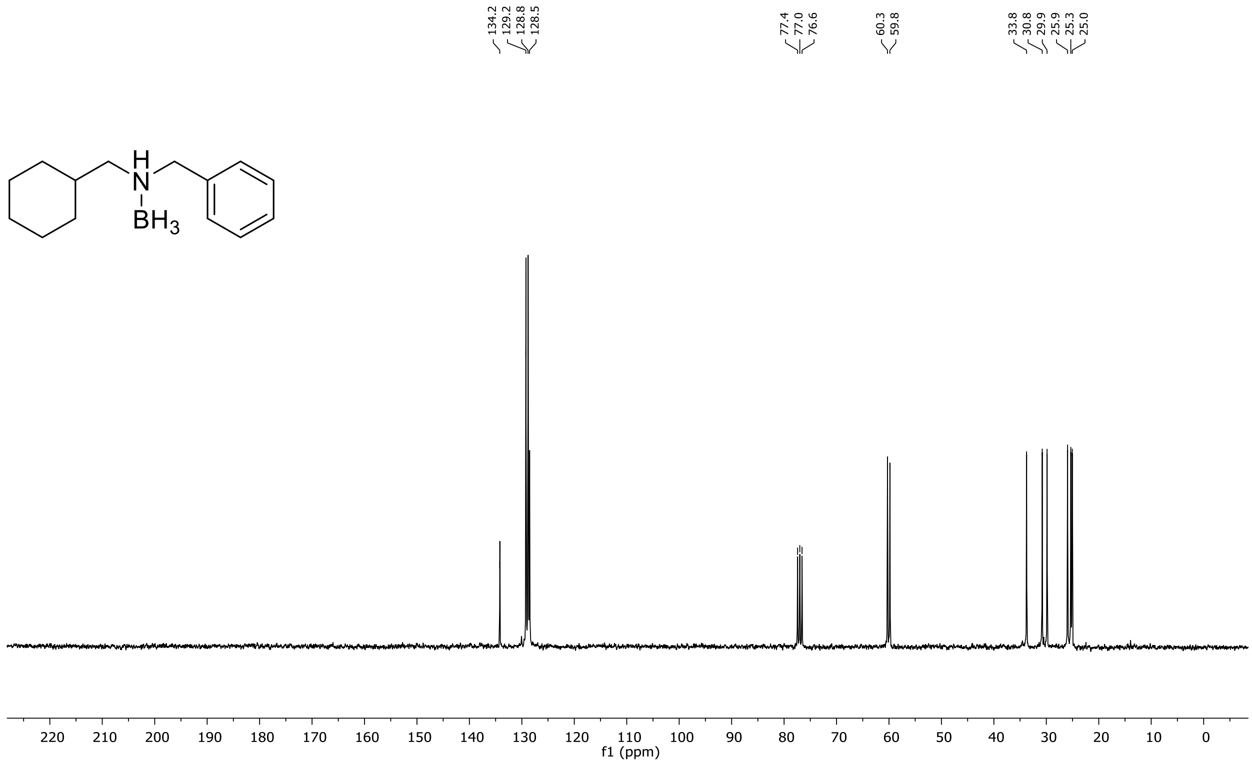

^{13}C {H} NMR (75 MHz, $CDCl_3$) borane-*N*-benzyl-1-cyclohexylmethanamine (**2k-BH$_3$**)

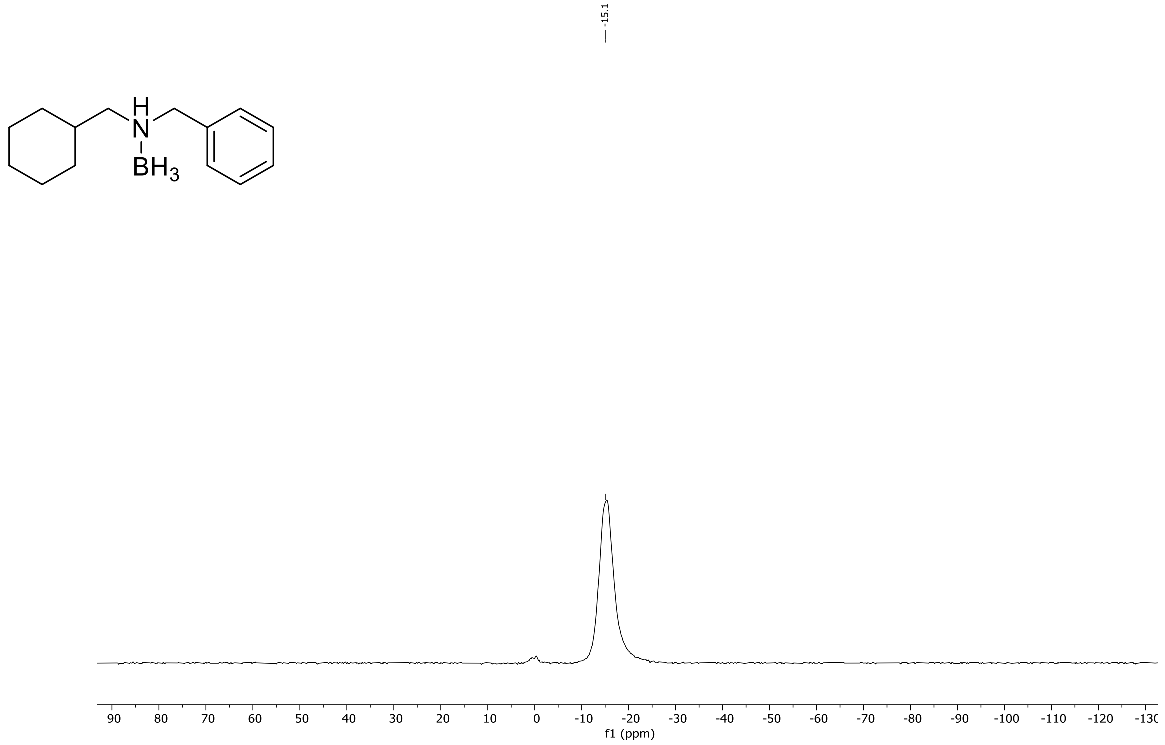

^{11}B NMR (96 MHz, $CDCl_3$) borane-*N*-benzyl-1-cyclohexylmethanamine (**2k-BH$_3$**)

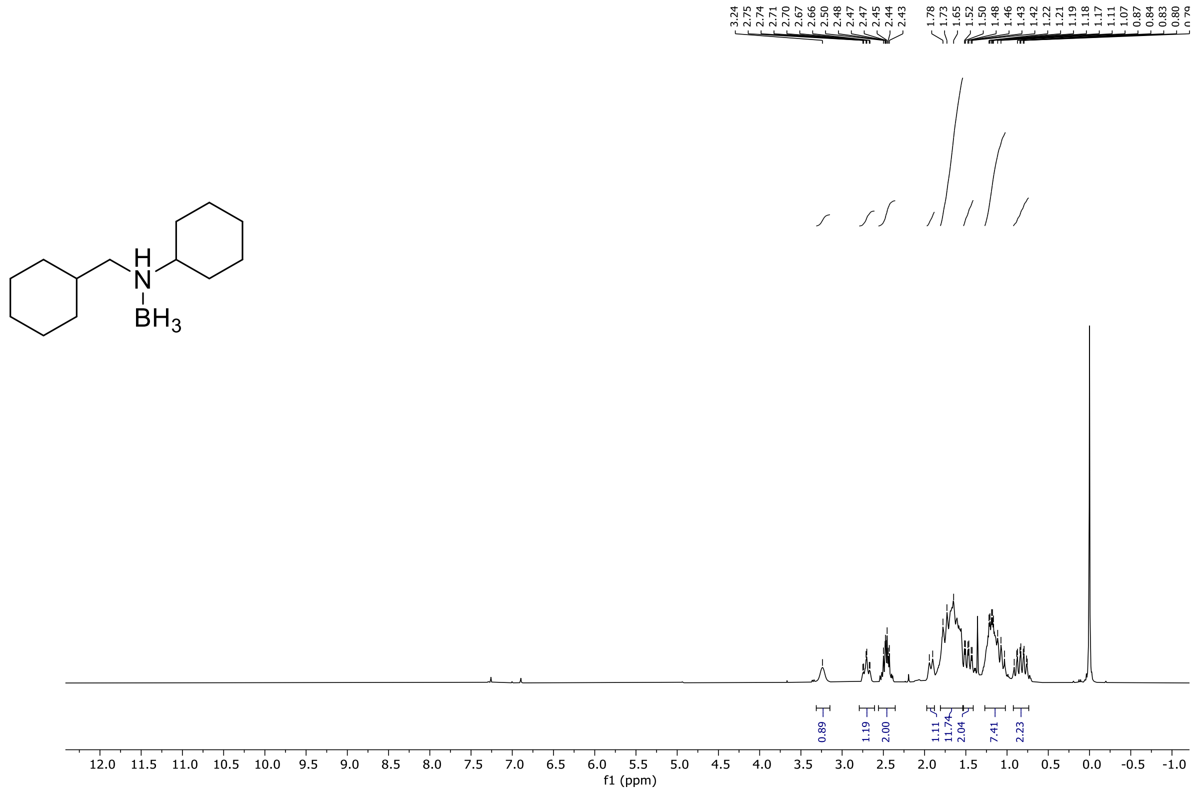

^{1}H NMR (300 MHz, $CDCl_3$) borane-*N*-(cyclohexylmethyl)cyclohexanamine (**2l-BH$_3$**)

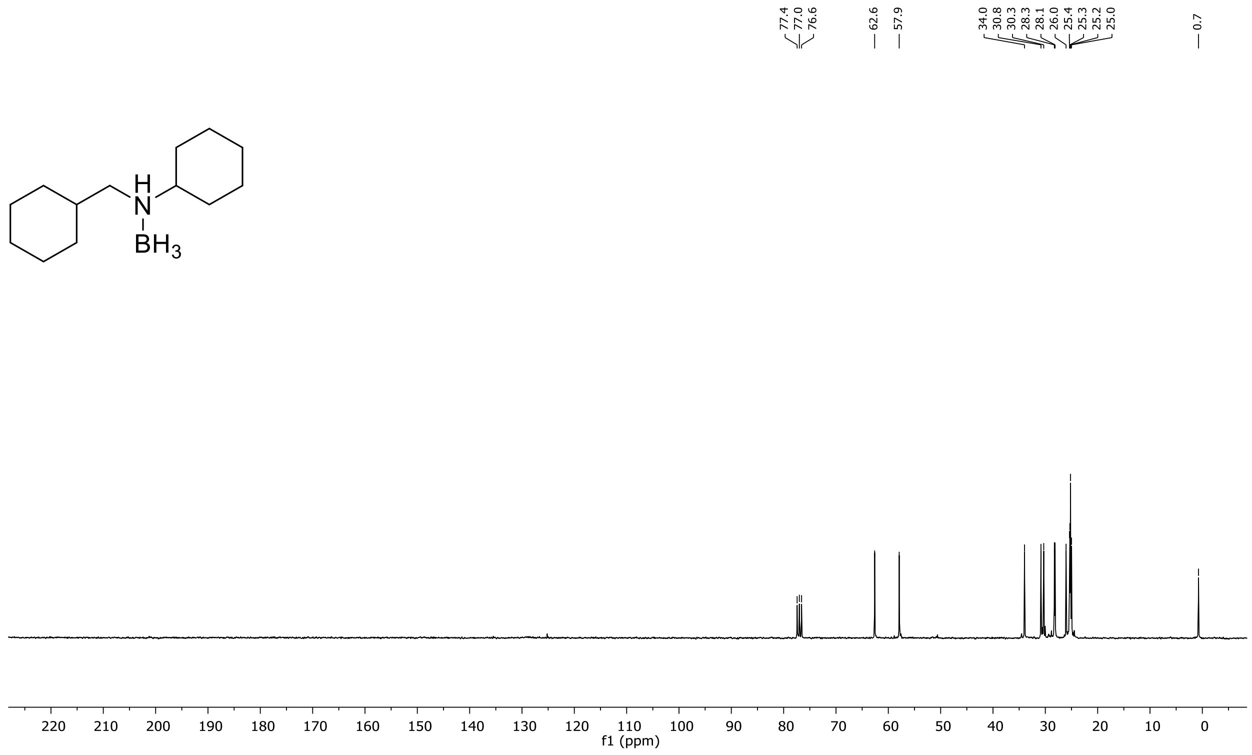

^{13}C {H} NMR (75 MHz, $CDCl_3$) borane-*N*-(cyclohexylmethyl)cyclohexanamine (**2l-BH$_3$**)

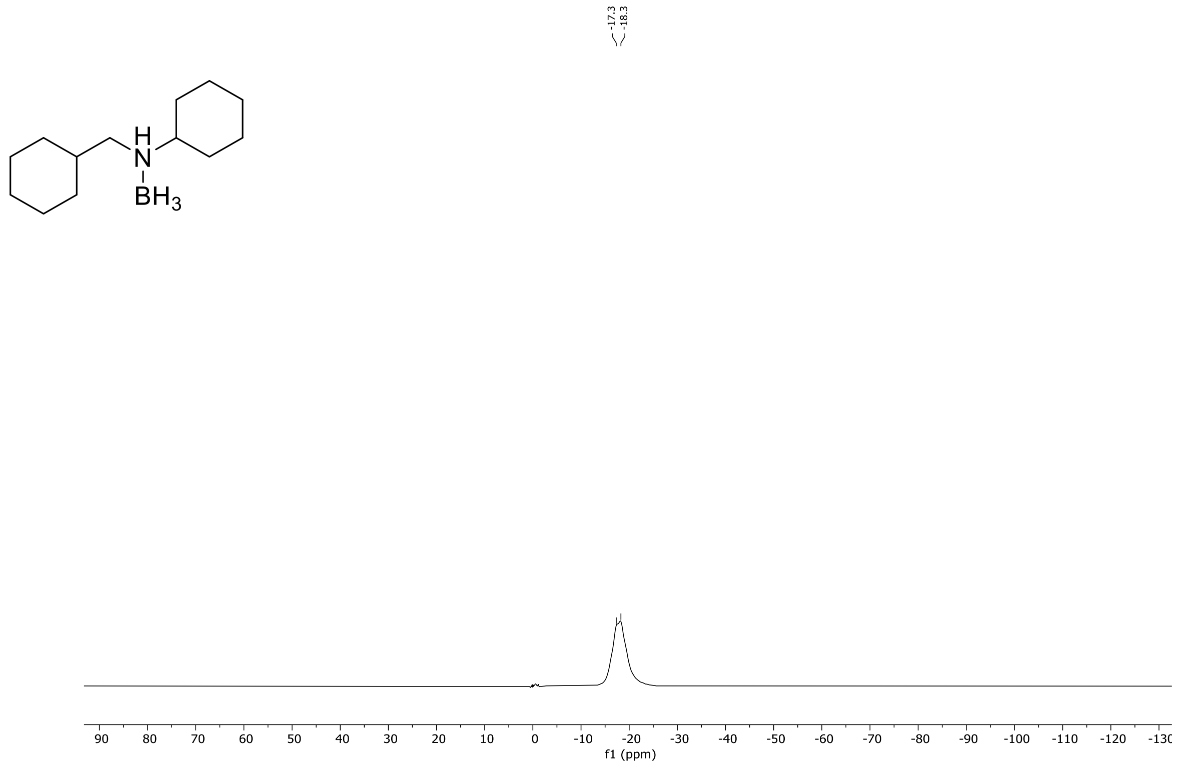

^{11}B NMR (96 MHz, $CDCl_3$) borane-*N*-(cyclohexylmethyl)cyclohexanamine (**2l-BH$_3$**)

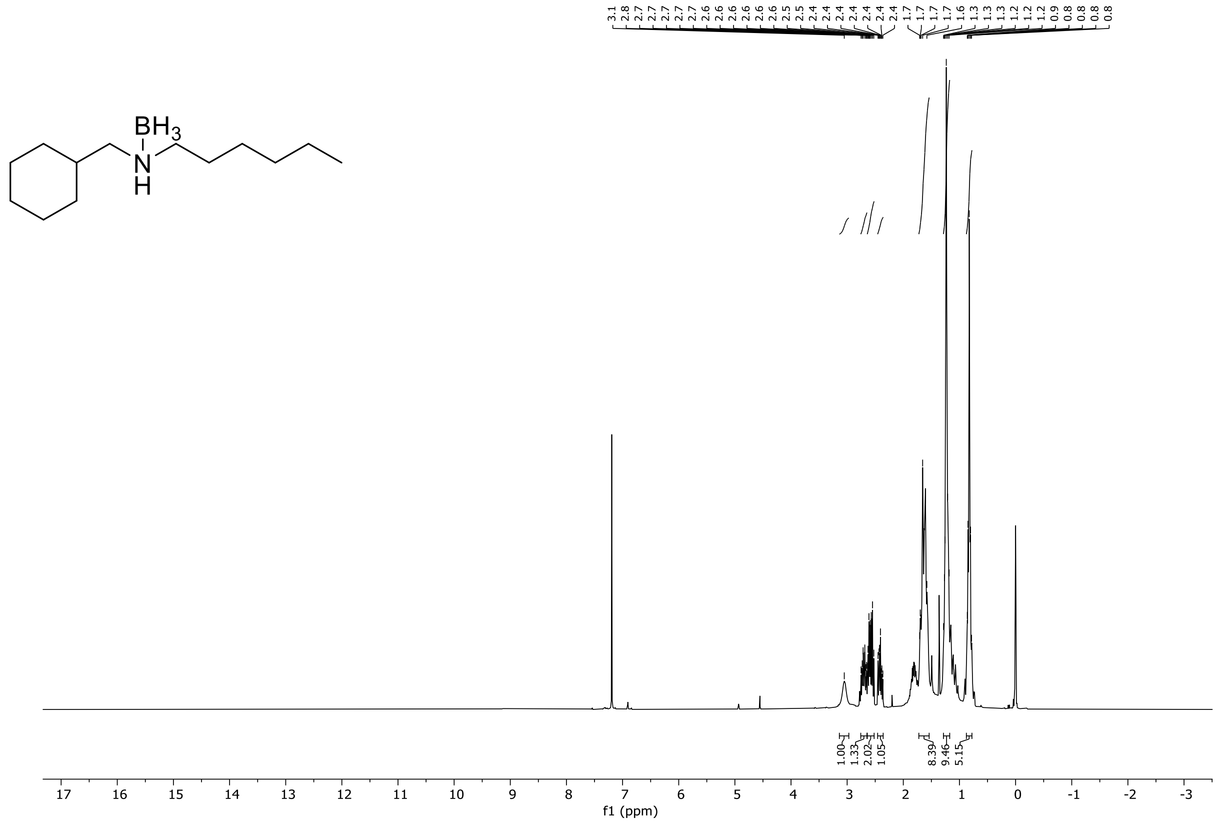

^{1}H NMR (300 MHz, $CDCl_3$) borane-*N*-(cyclohexylmethyl)hexan-1-amine (**2m-BH$_3$**)

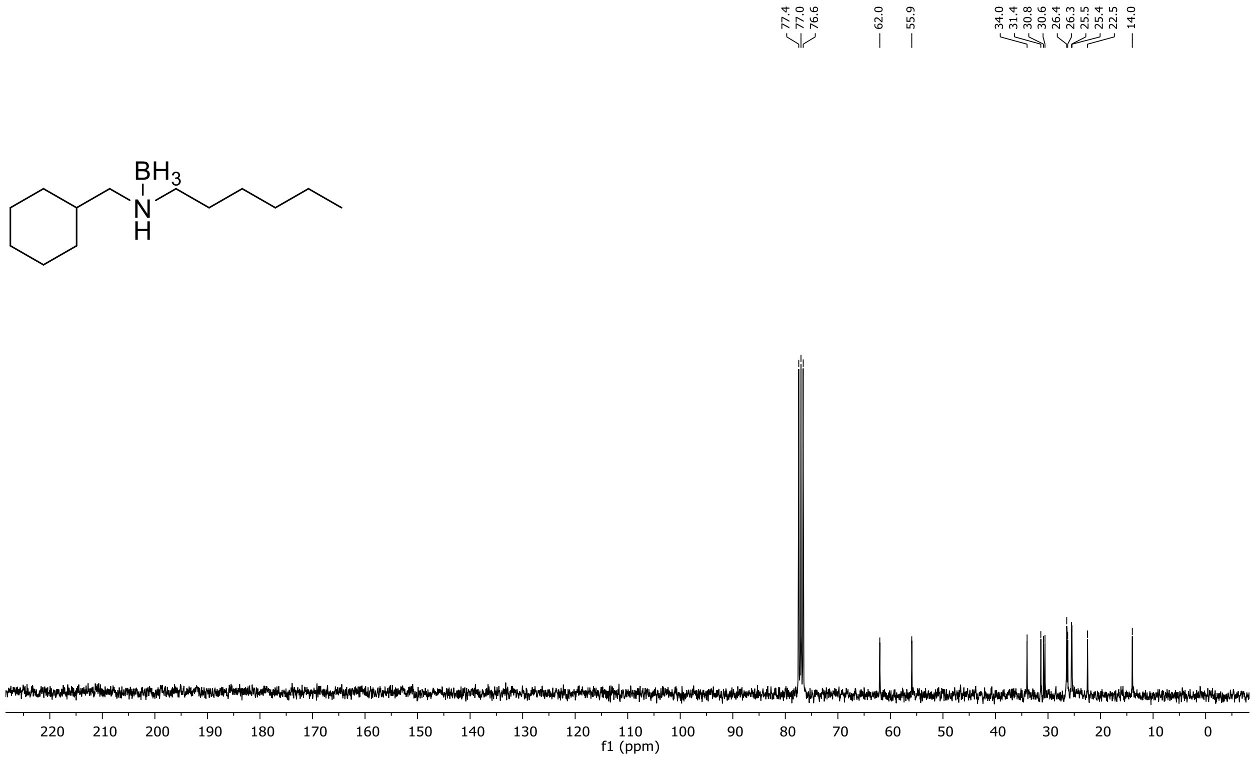

^{13}C {H} NMR (75 MHz, $CDCl_3$) borane-*N*-(cyclohexylmethyl)hexan-1-amine (**2m-BH$_3$**)

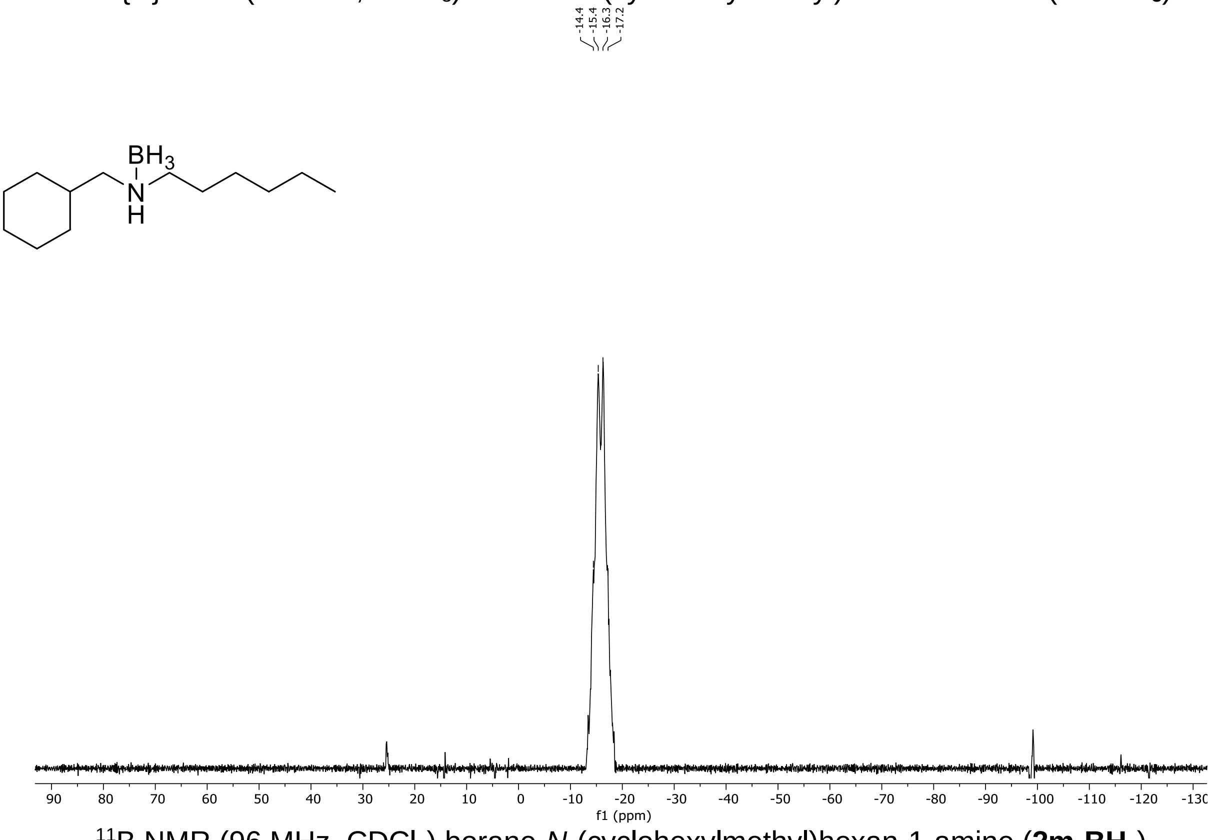

^{11}B NMR (96 MHz, $CDCl_3$) borane-*N*-(cyclohexylmethyl)hexan-1-amine (**2m-BH$_3$**)

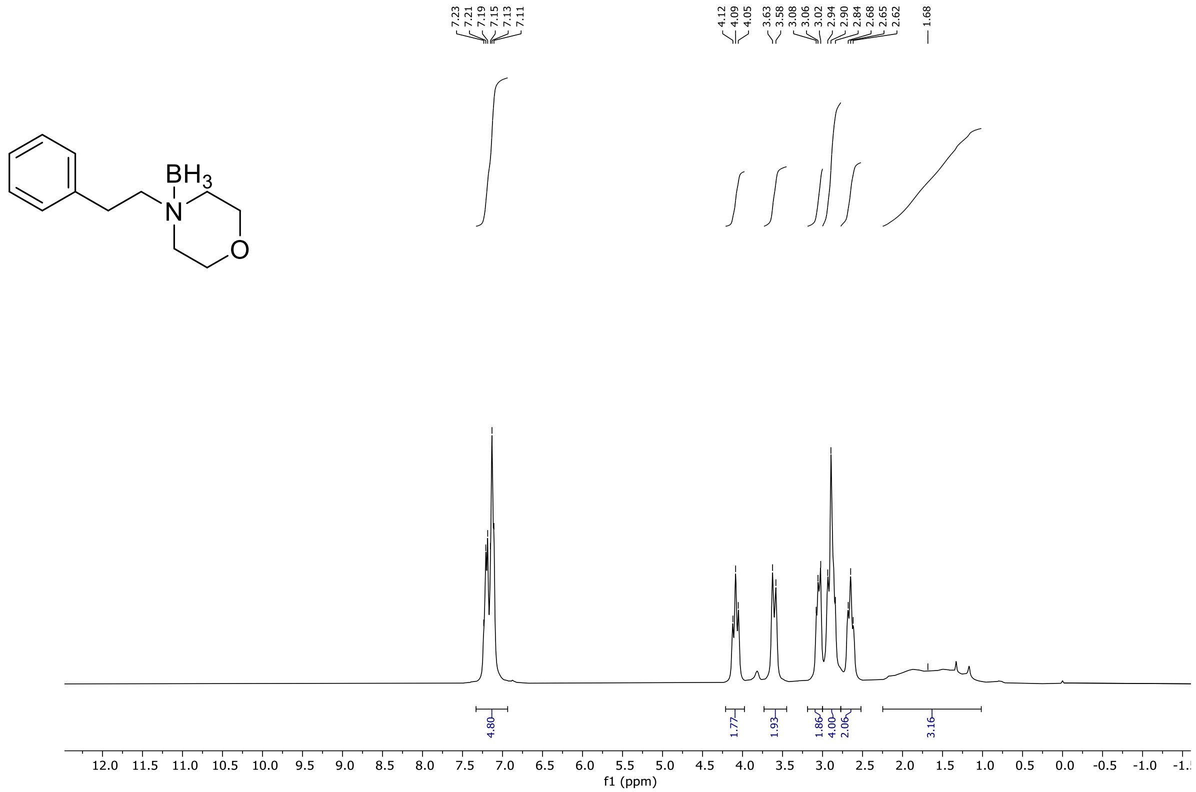

^{1}H NMR (300 MHz, $CDCl_3$) borane-4-phenethylmorpholine (**2n-BH$_3$**)

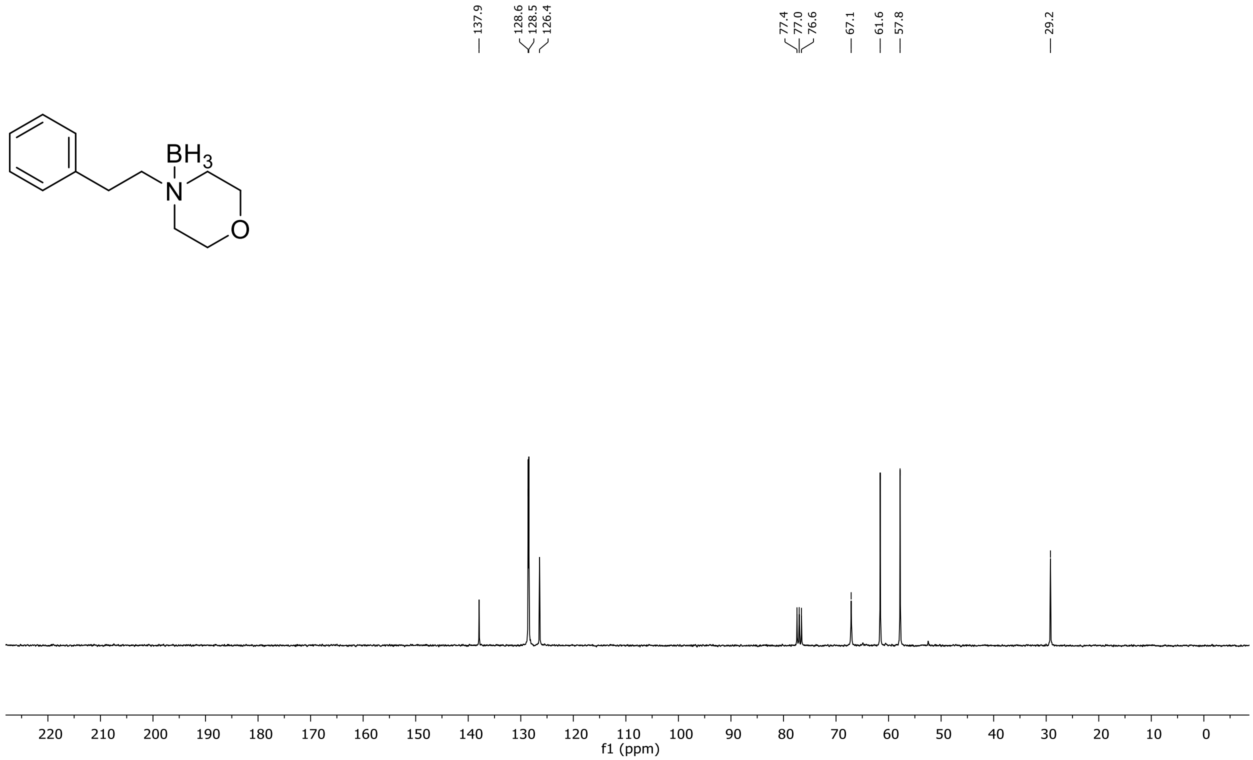

^{13}C {H} NMR (75 MHz, $CDCl_3$) borane-4-phenethylmorpholine (**2n-BH$_3$**)

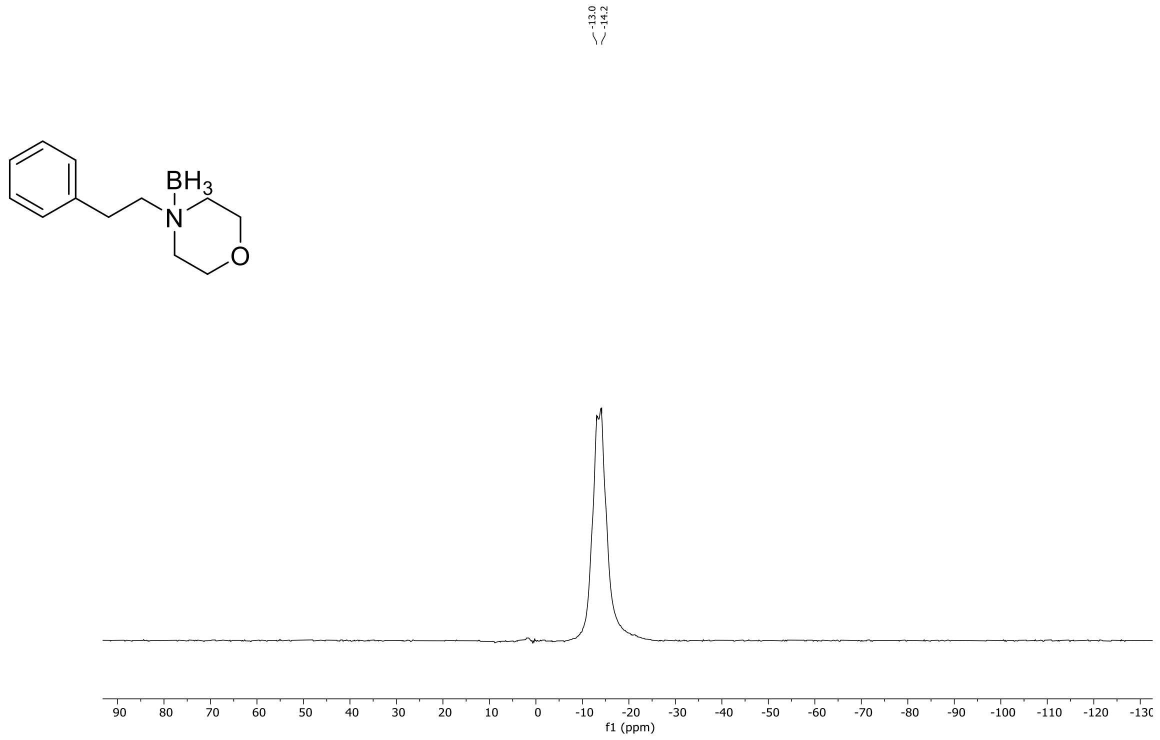

^{11}B NMR (96 MHz, $CDCl_3$) borane-4-phenethylmorpholine (**2n-BH$_3$**)

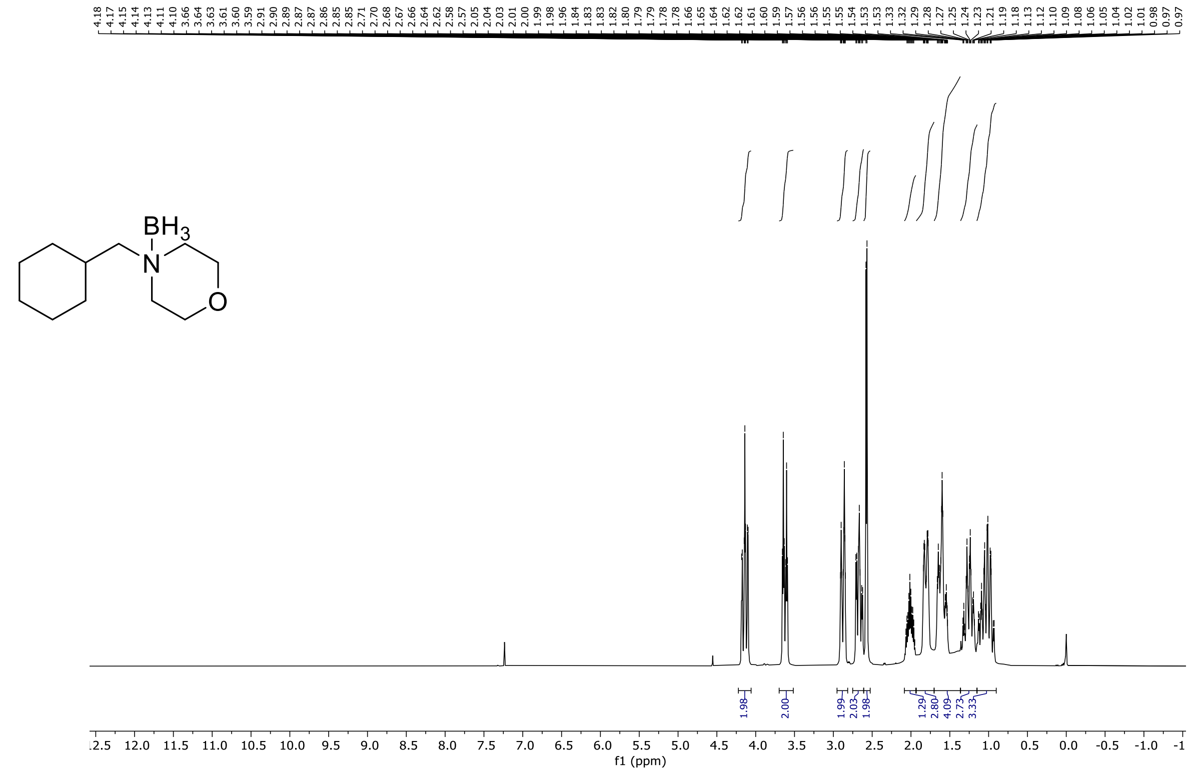

^{1}H NMR (300 MHz, $CDCl_3$) borane-4-(cyclohexylmethyl)morpholine (**2o-BH$_3$**)

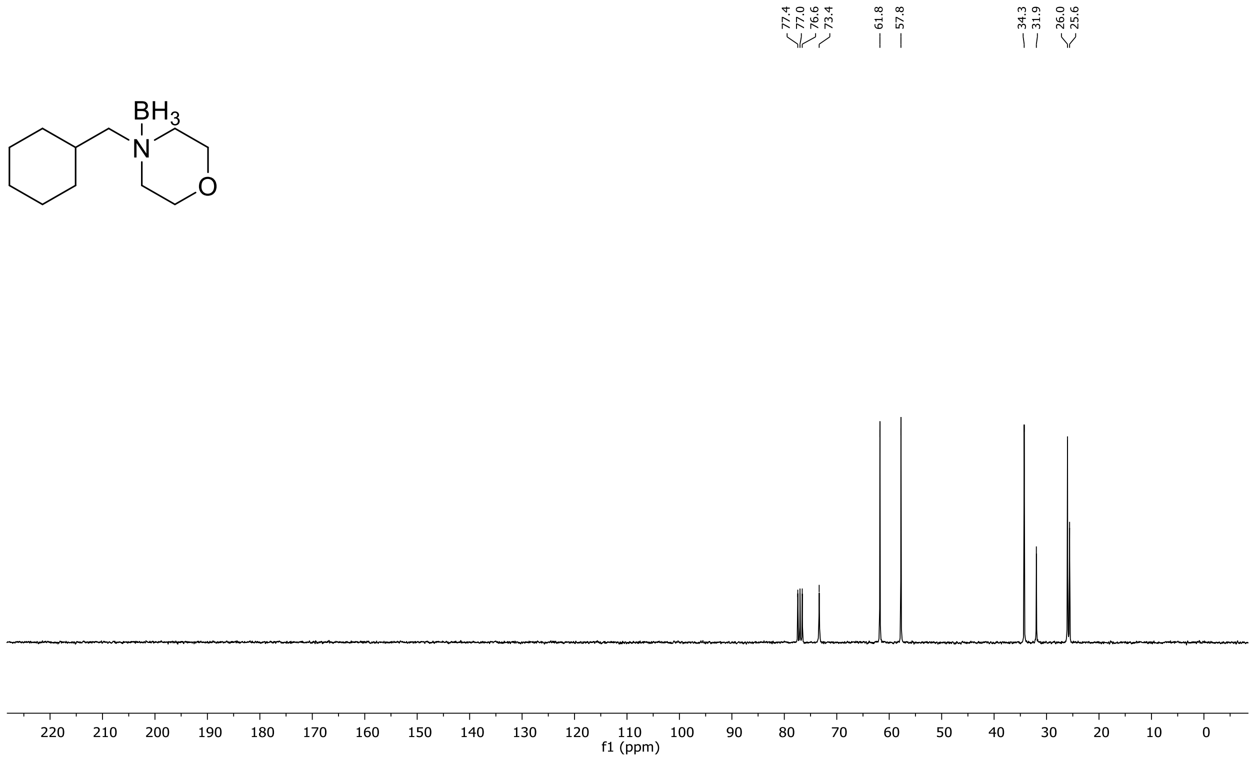

^{13}C {H} NMR (75 MHz, $CDCl_3$) borane-4-(cyclohexylmethyl)morpholine (**2o-BH₃**)

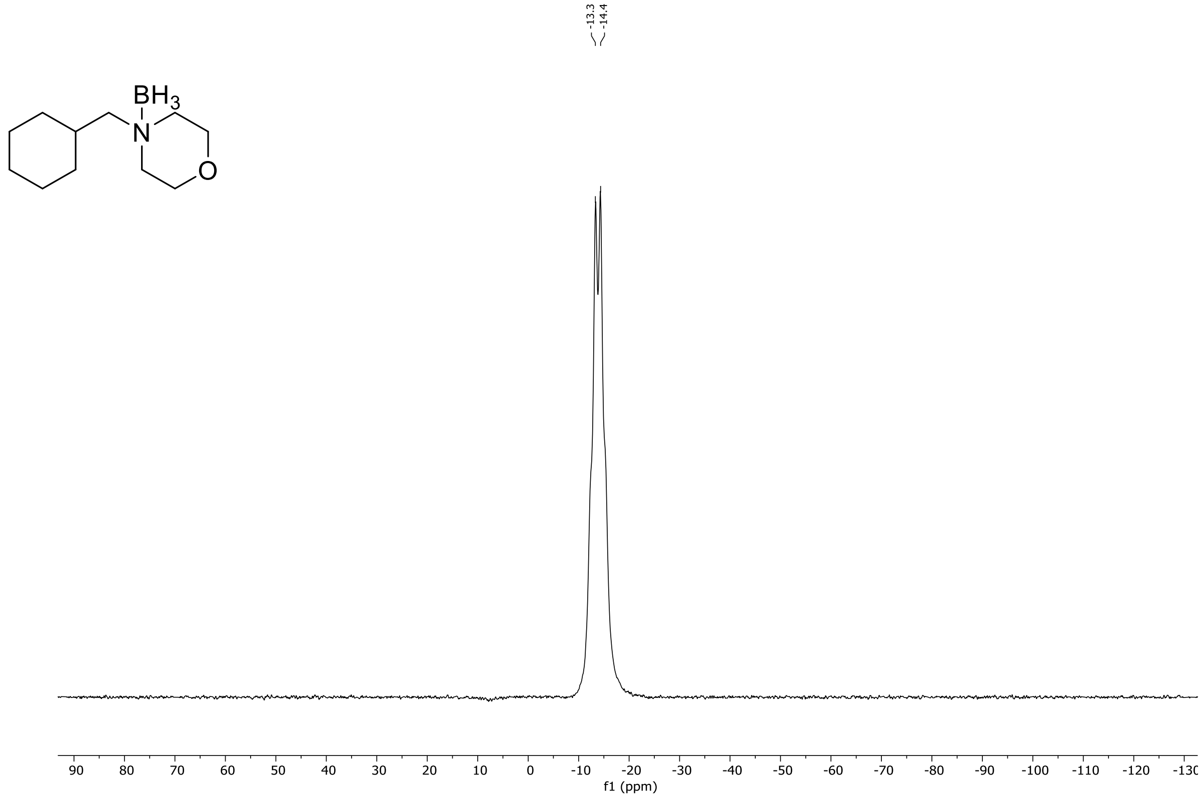

^{11}B NMR (96 MHz, $CDCl_3$) borane-4-(cyclohexylmethyl)morpholine (**2o-BH₃)**

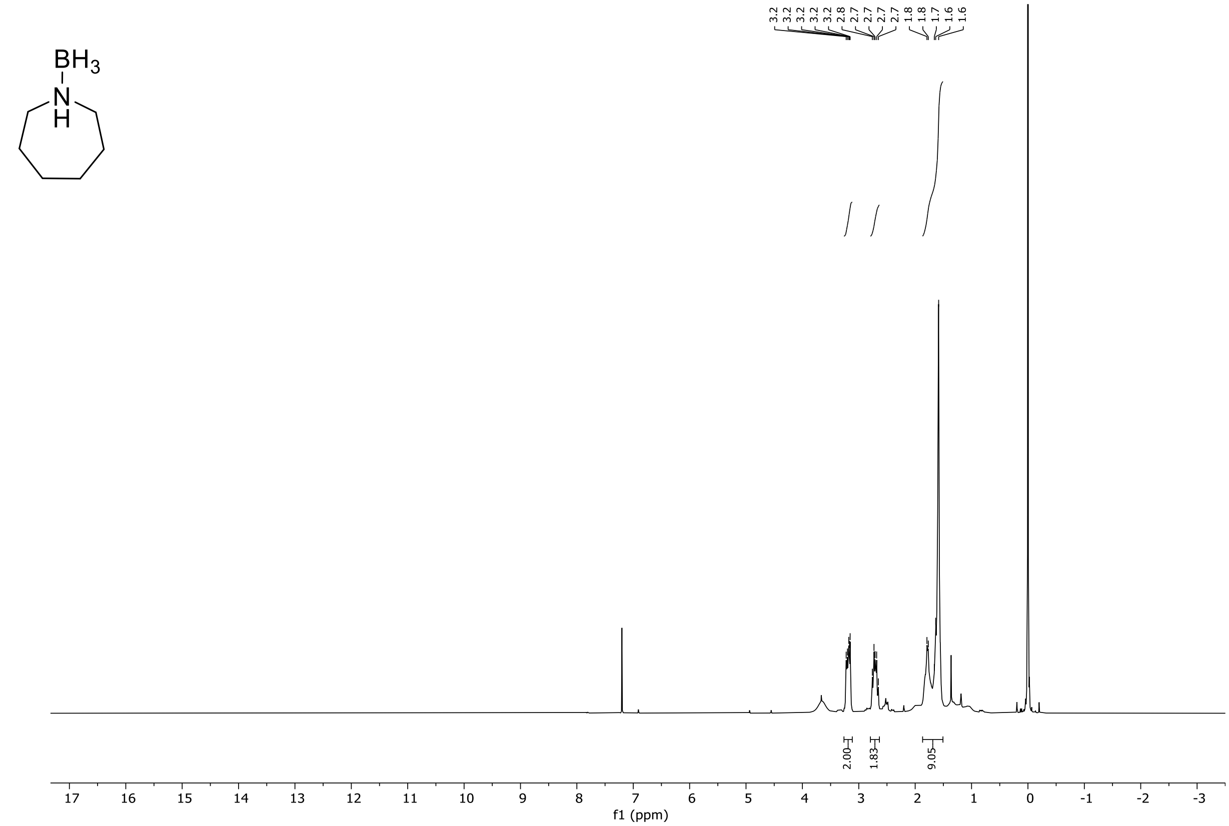

^{1}H NMR (300 MHz, $CDCl_3$) borane-azepane (**2p-BH$_3$**)

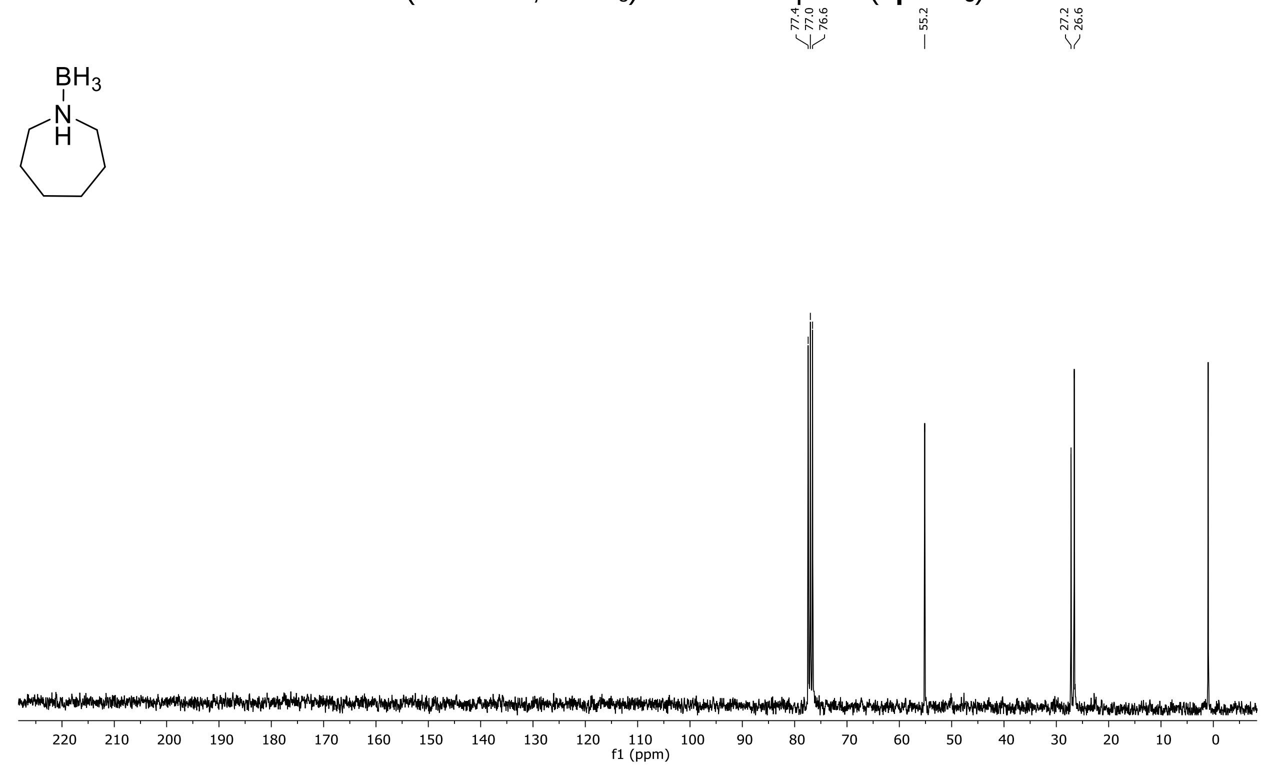

^{13}C {H} NMR (75 MHz, $CDCl_3$) borane-azepane (**2p-BH$_3$**)

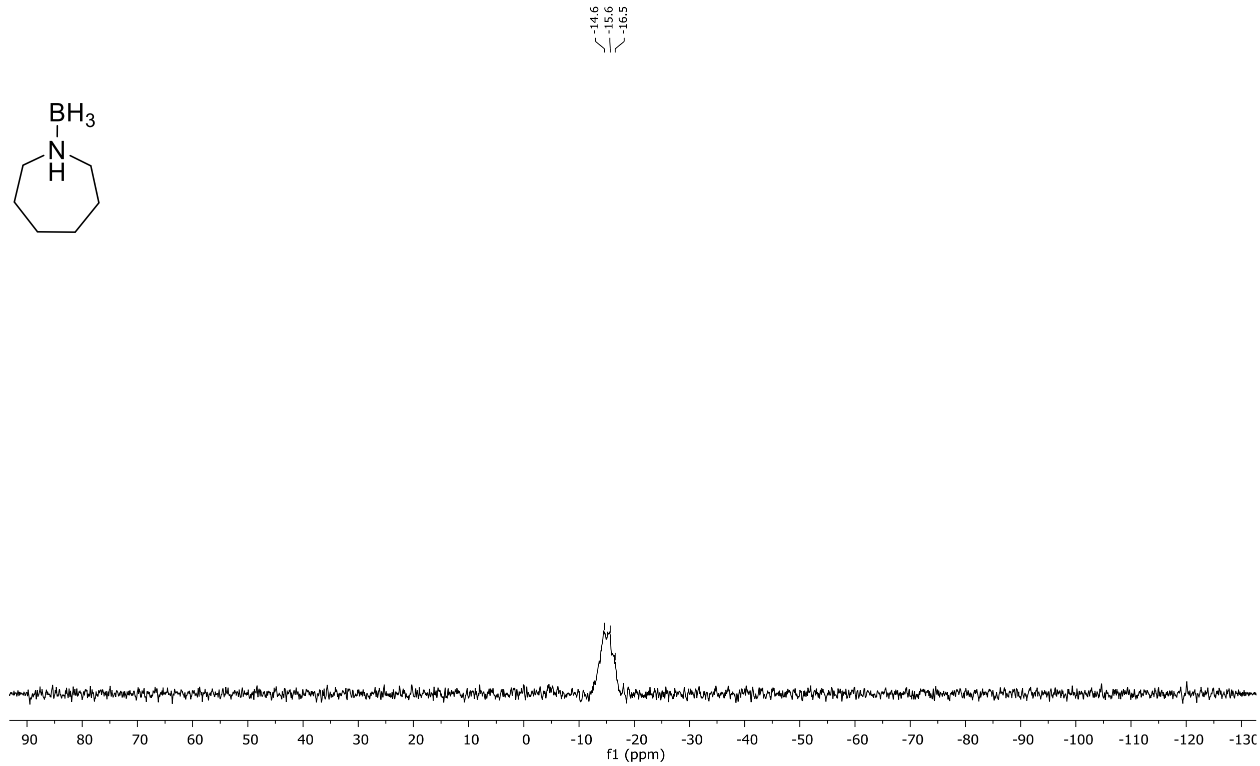

^{11}B NMR (96 MHz, $CDCl_3$) borane-azepane (**2p-BH$_3$**)

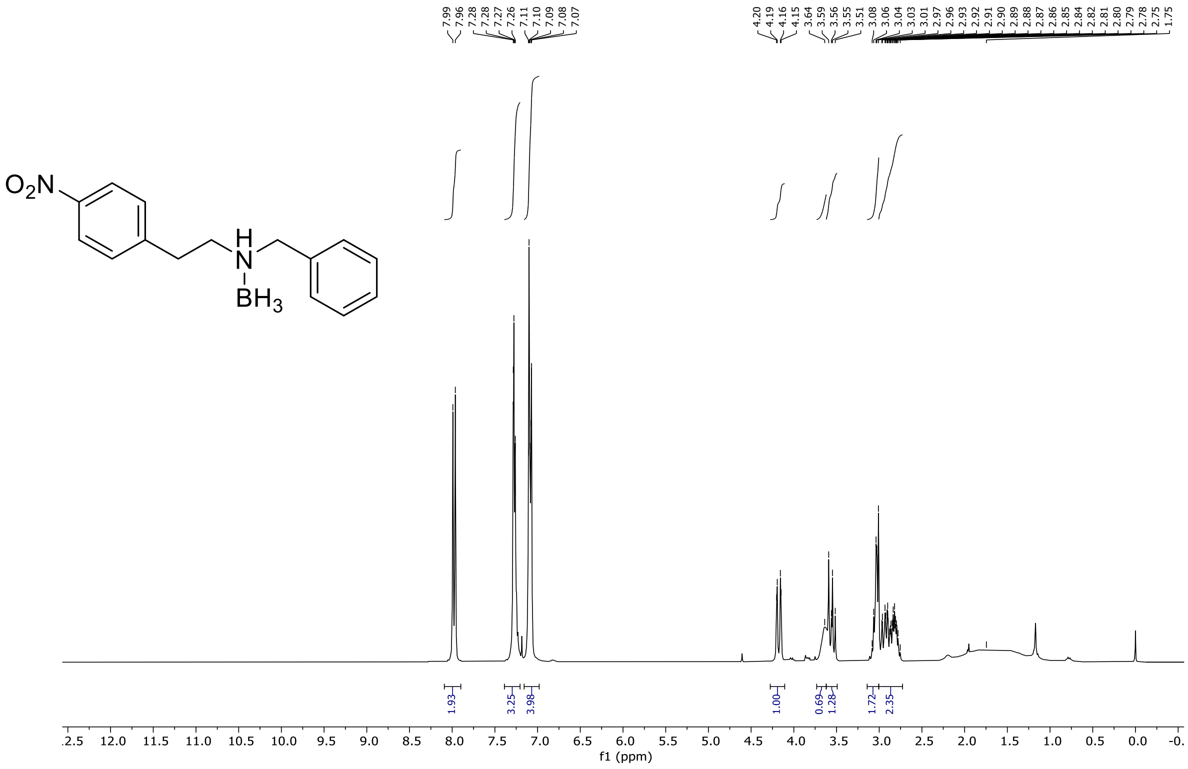

^{1}H NMR (300 MHz, $CDCl_3$) borane-*N*-benzyl-2-(4-nitrophenyl)ethan-1-amine (**2q-BH$_3$**)

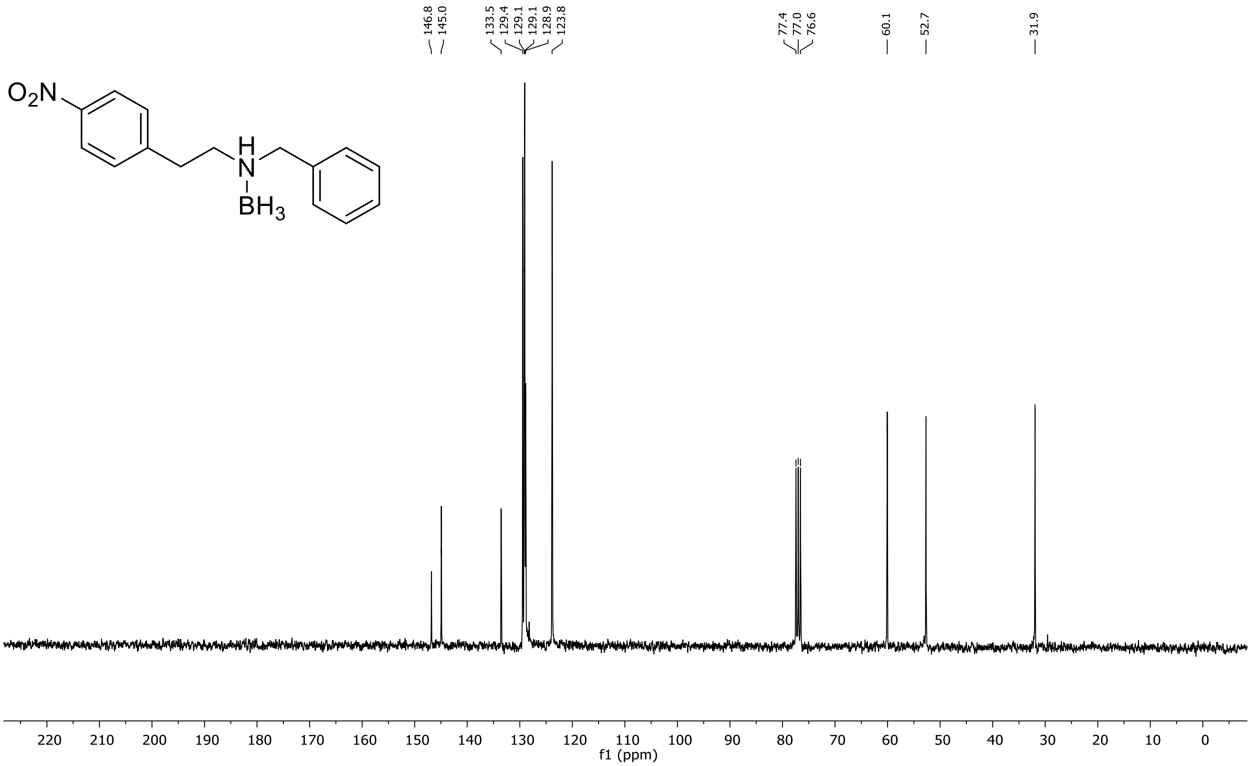

^{13}C {H} NMR (75 MHz, $CDCl_3$) borane-*N*-benzyl-2-(4-nitrophenyl)ethan-1-amine (**2q-BH$_3$**)

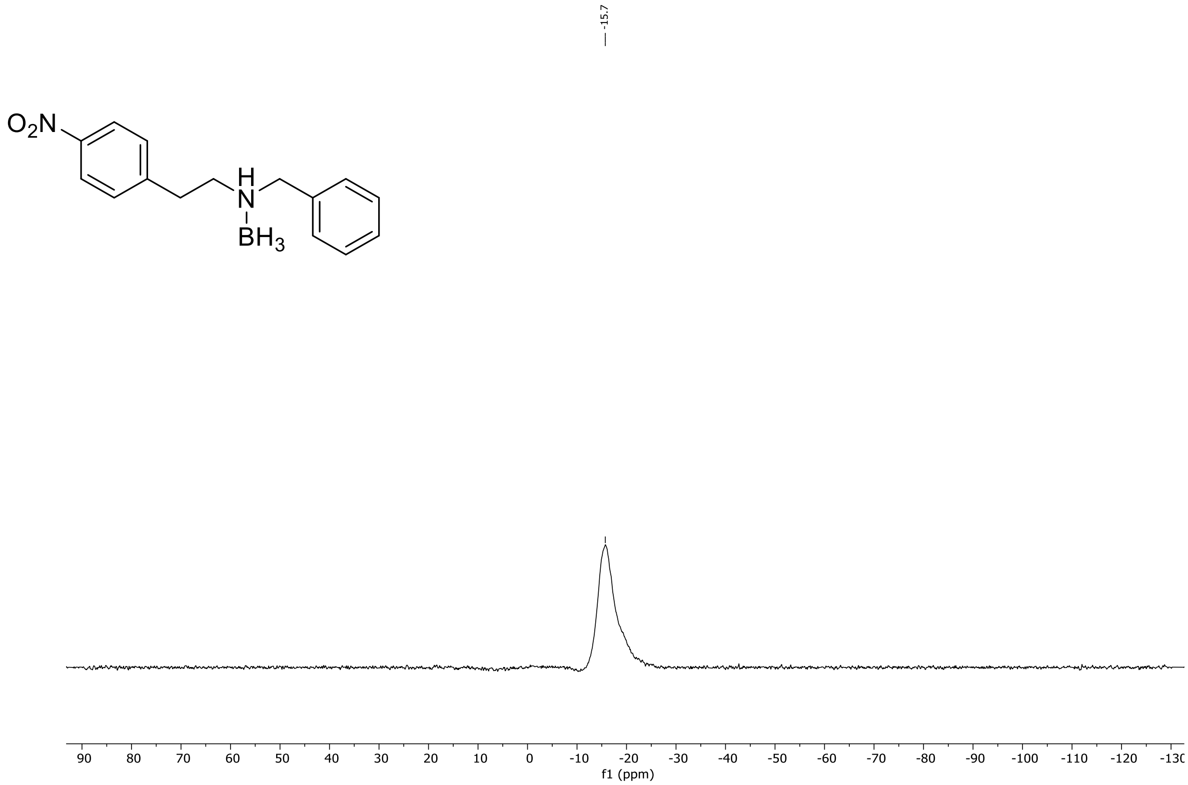

^{11}B NMR (96 MHz, $CDCl_3$) borane-*N*-benzyl-2-(4-nitrophenyl)ethan-1-amine (**2q-BH$_3$**)

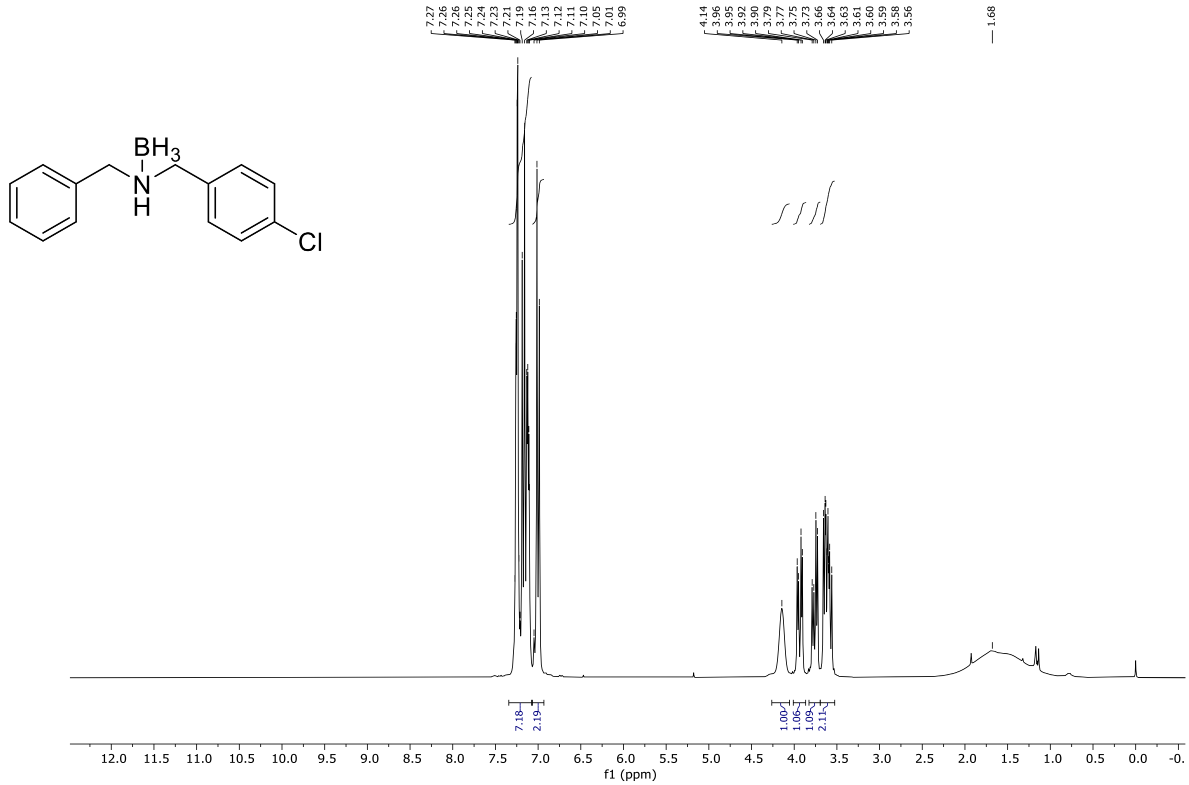

^{1}H NMR (300 MHz, $CDCl_3$) borane-*N*-benzyl-1-(4-chlorophenyl)methanamine (**2r-BH$_3$**)

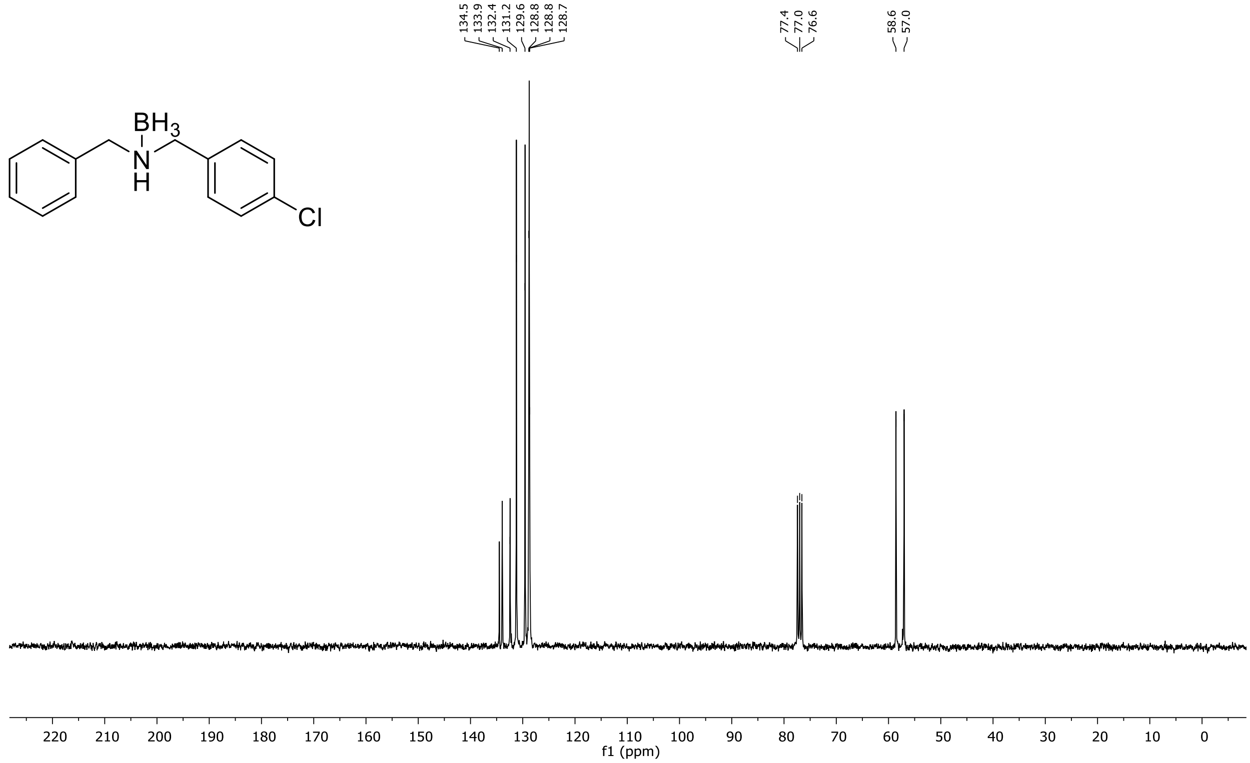

^{13}C {H} NMR (75 MHz, $CDCl_3$) borane-*N*-benzyl-1-(4-chlorophenyl)methanamine (**2r-BH$_3$**)

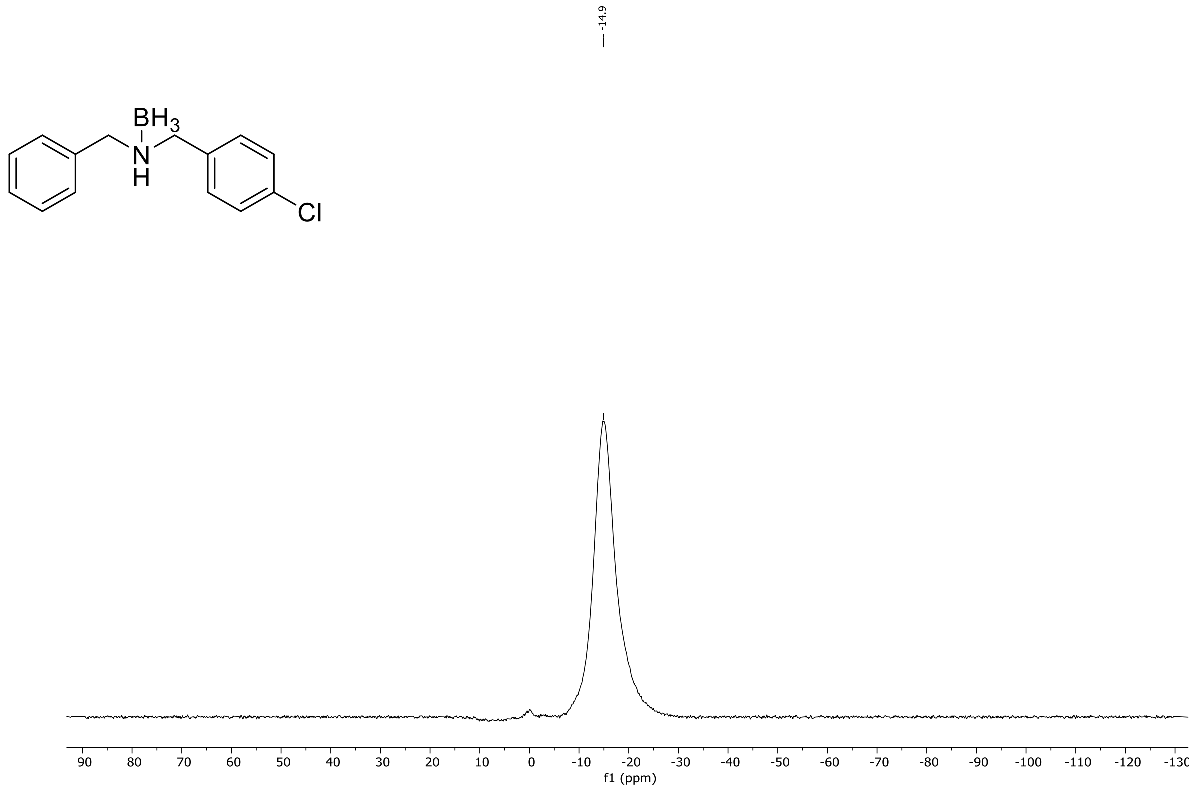

^{11}B NMR (96 MHz, $CDCl_3$) borane-*N*-benzyl-1-(4-chlorophenyl)methanamine (**2r-BH$_3$**)

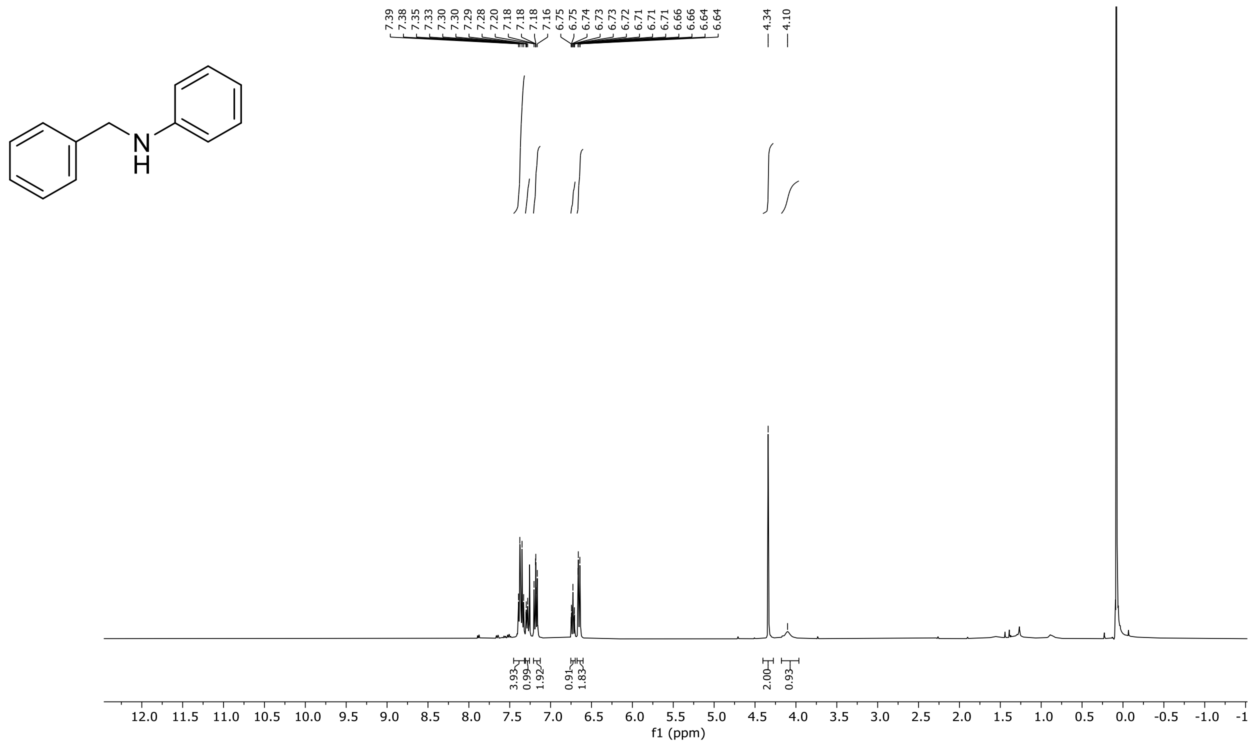

^{1}H NMR (400 MHz, $CDCl_3$) *N*-benzylaniline (**2s**)

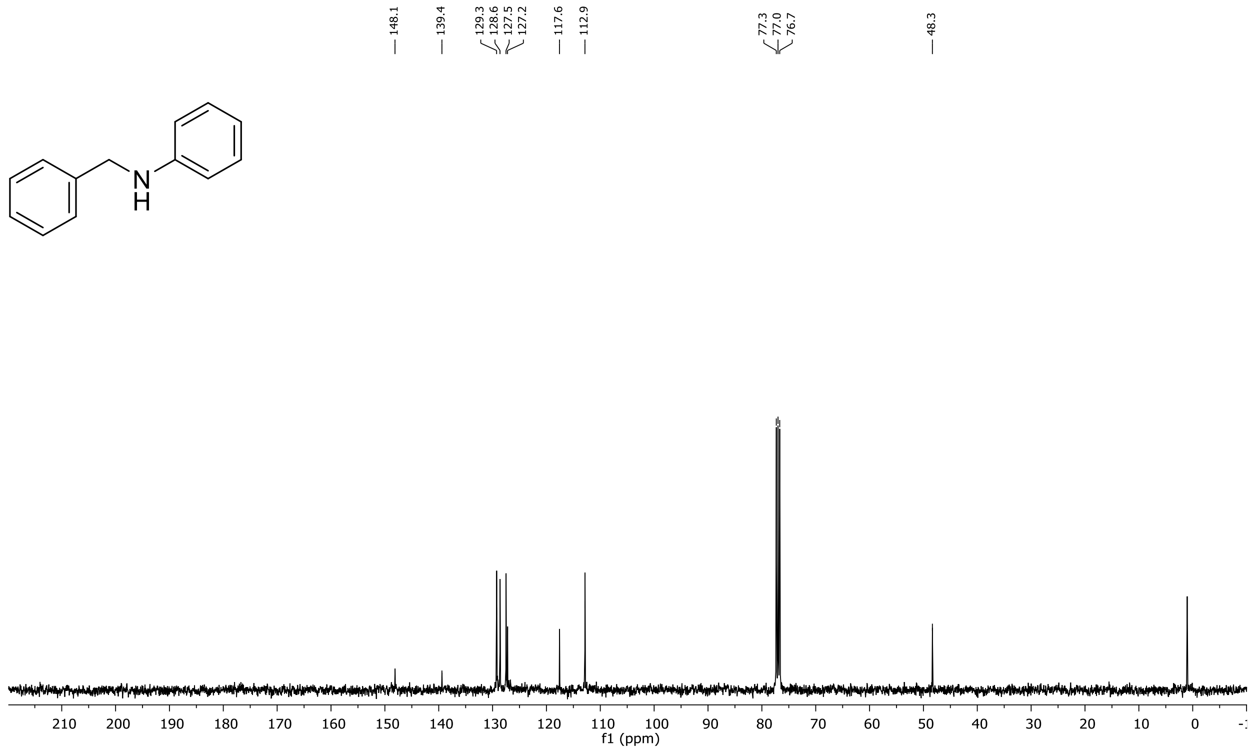

^{13}C {H} NMR (101 MHz, $CDCl_3$) *N*-benzylaniline (**2s**)

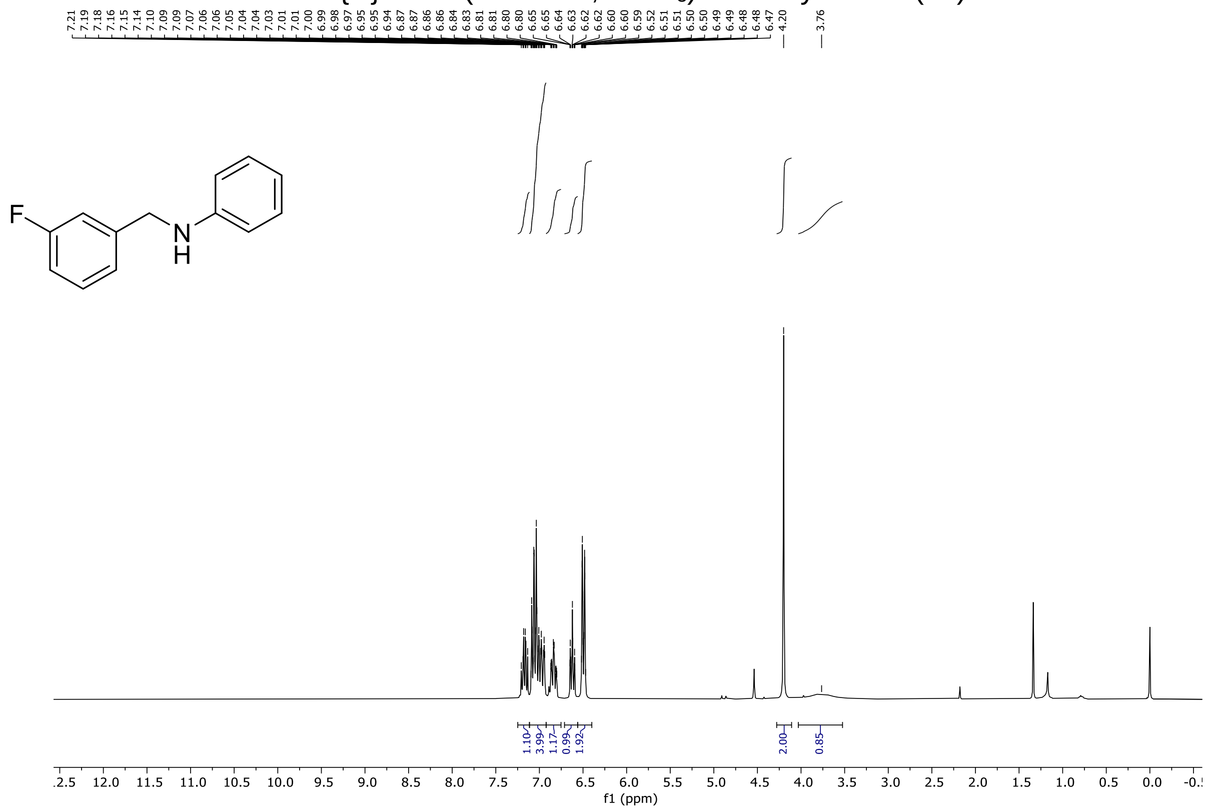

^{1}H NMR (300 MHz, $CDCl_3$) *N*-(3-fluorobenzyl)aniline (**2t**)

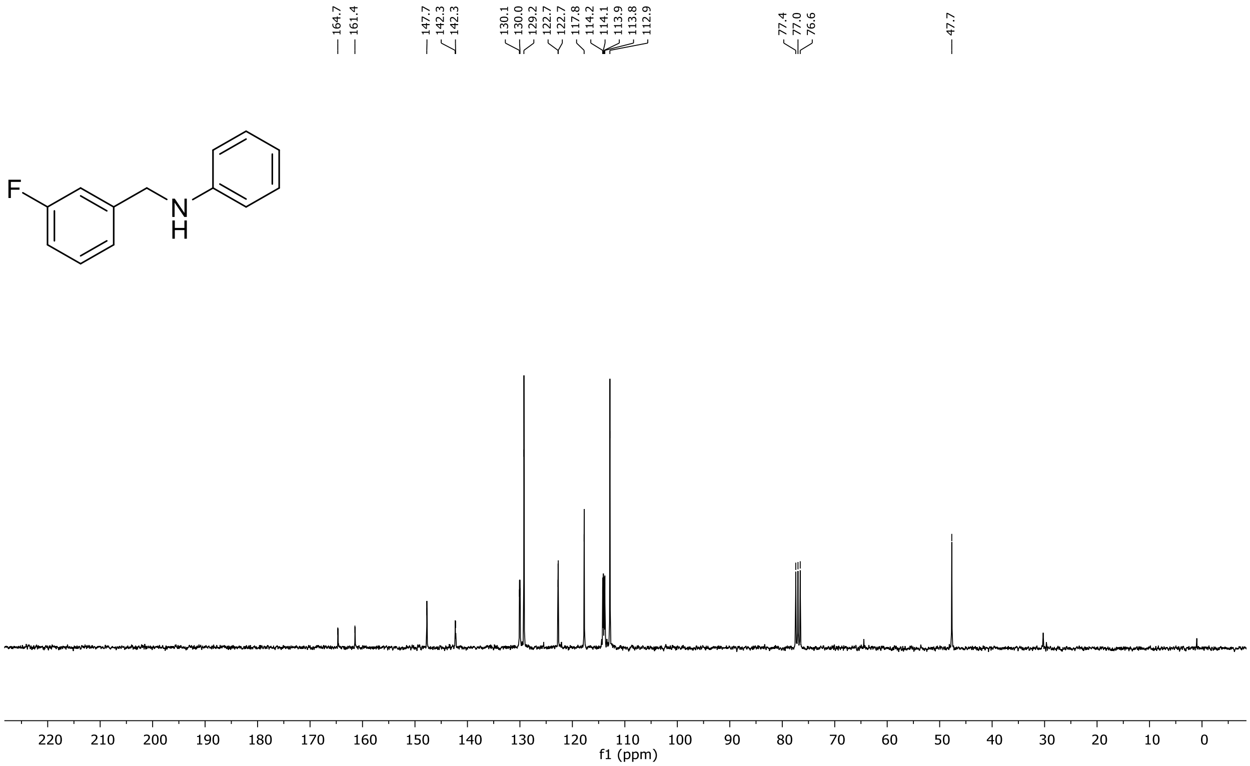

^{13}C {H} NMR (75 MHz, $CDCl_3$) *N*-(3-fluorobenzyl)aniline (**2t**)

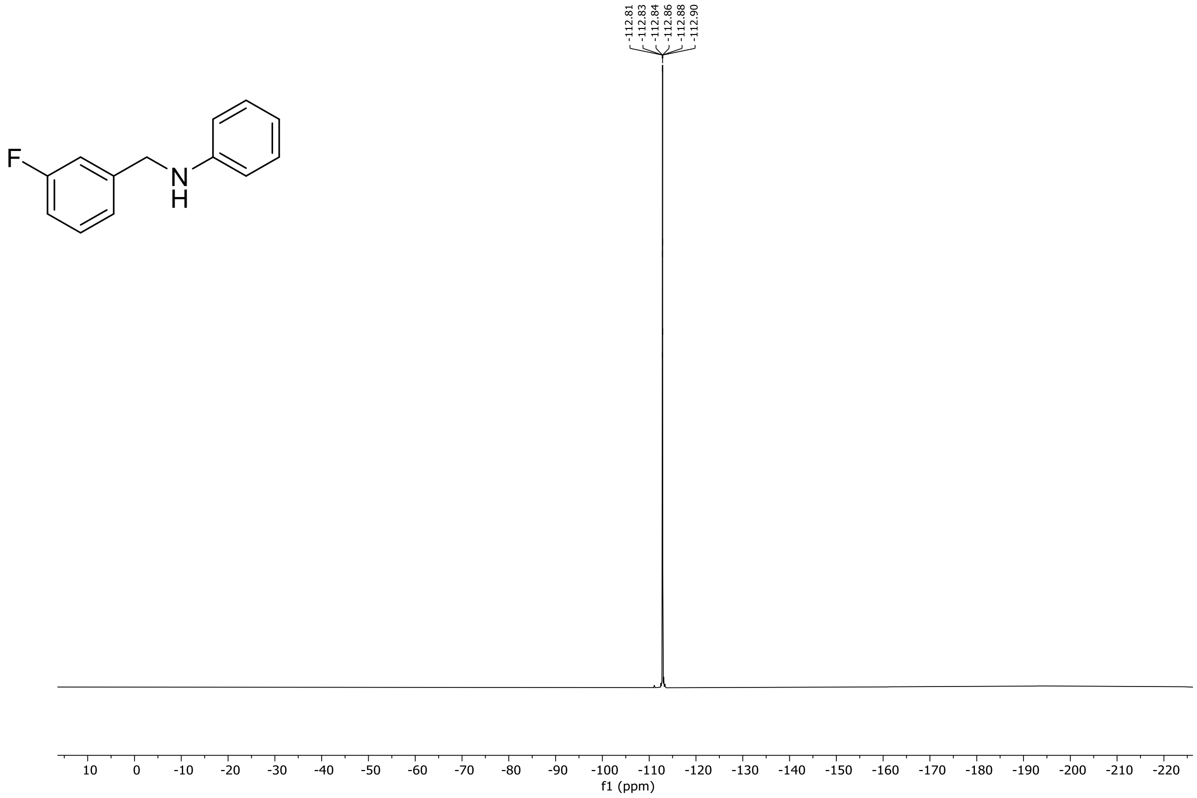

^{19}F NMR (282 MHz, $CDCl_3$) *N*-(3-fluorobenzyl)aniline (**2t**)

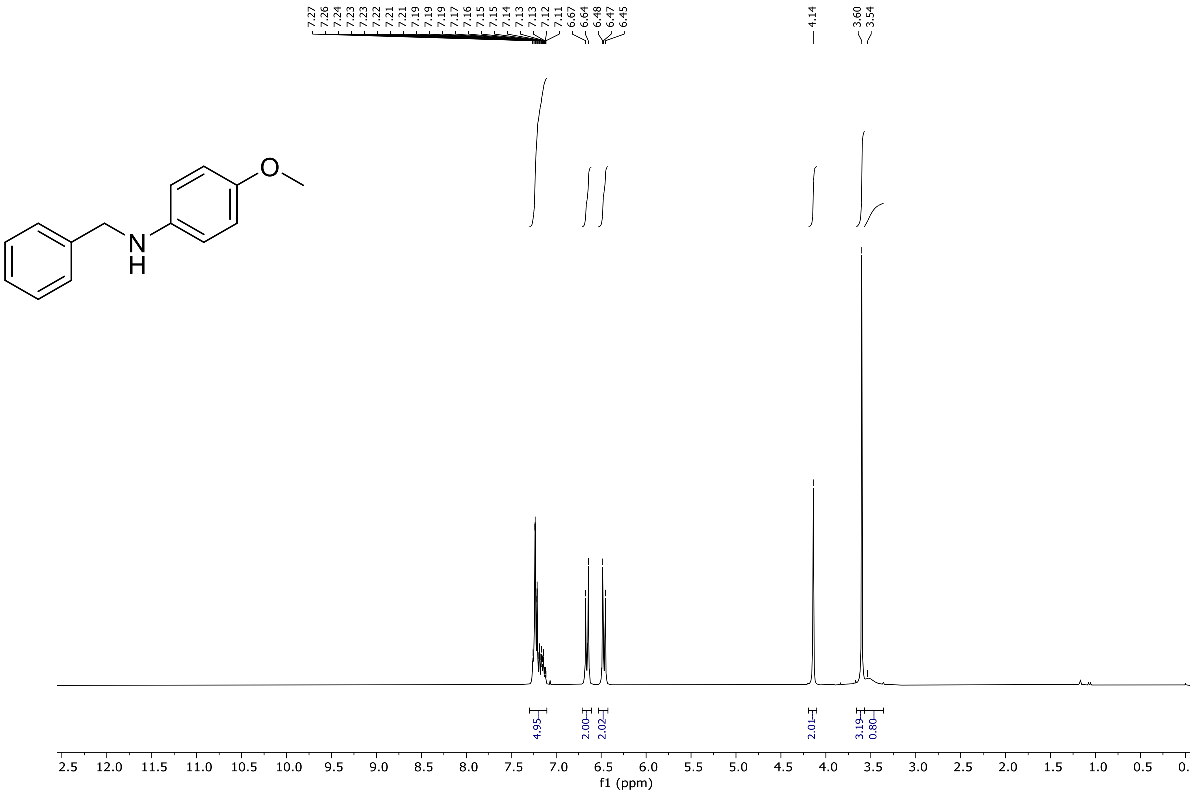

^{1}H NMR (300 MHz, $CDCl_3$) *N*-benzyl-4-methoxyaniline (**2u**)

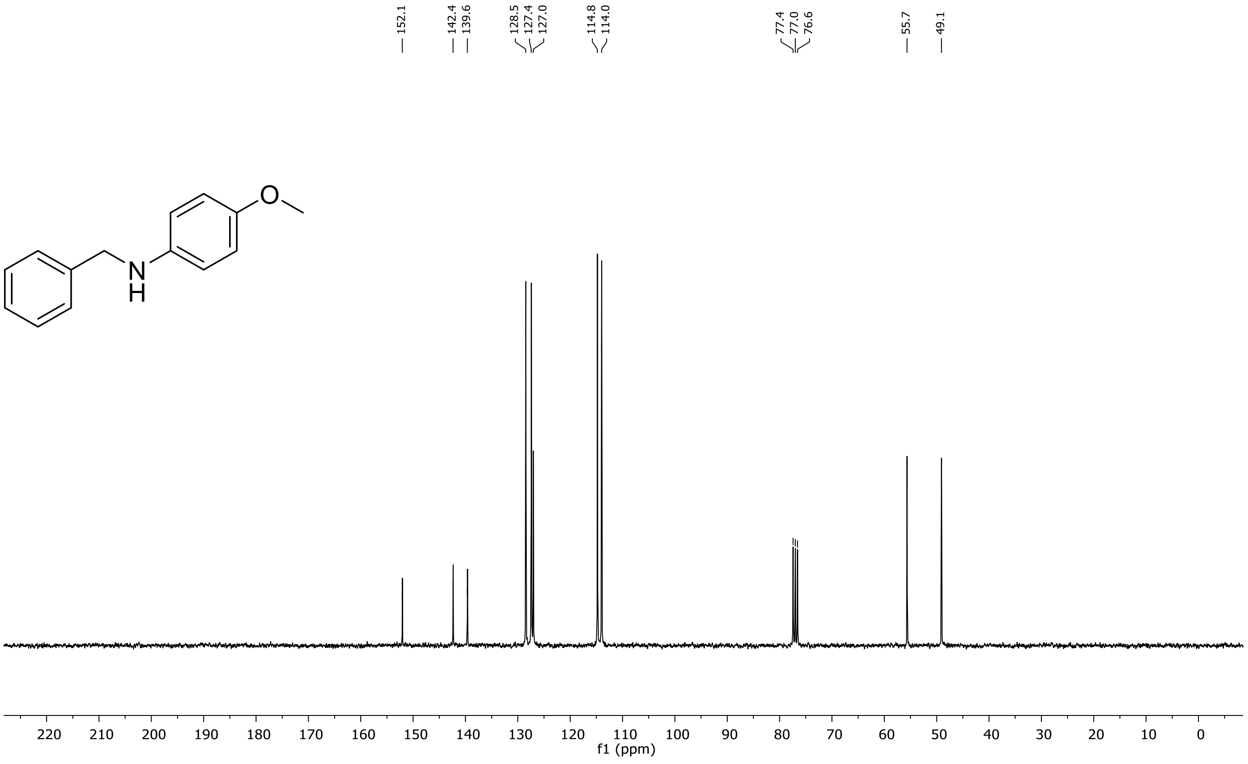

^{13}C {H} NMR (75 MHz, $CDCl_3$) *N*-benzyl-4-methoxyaniline (**2u**)

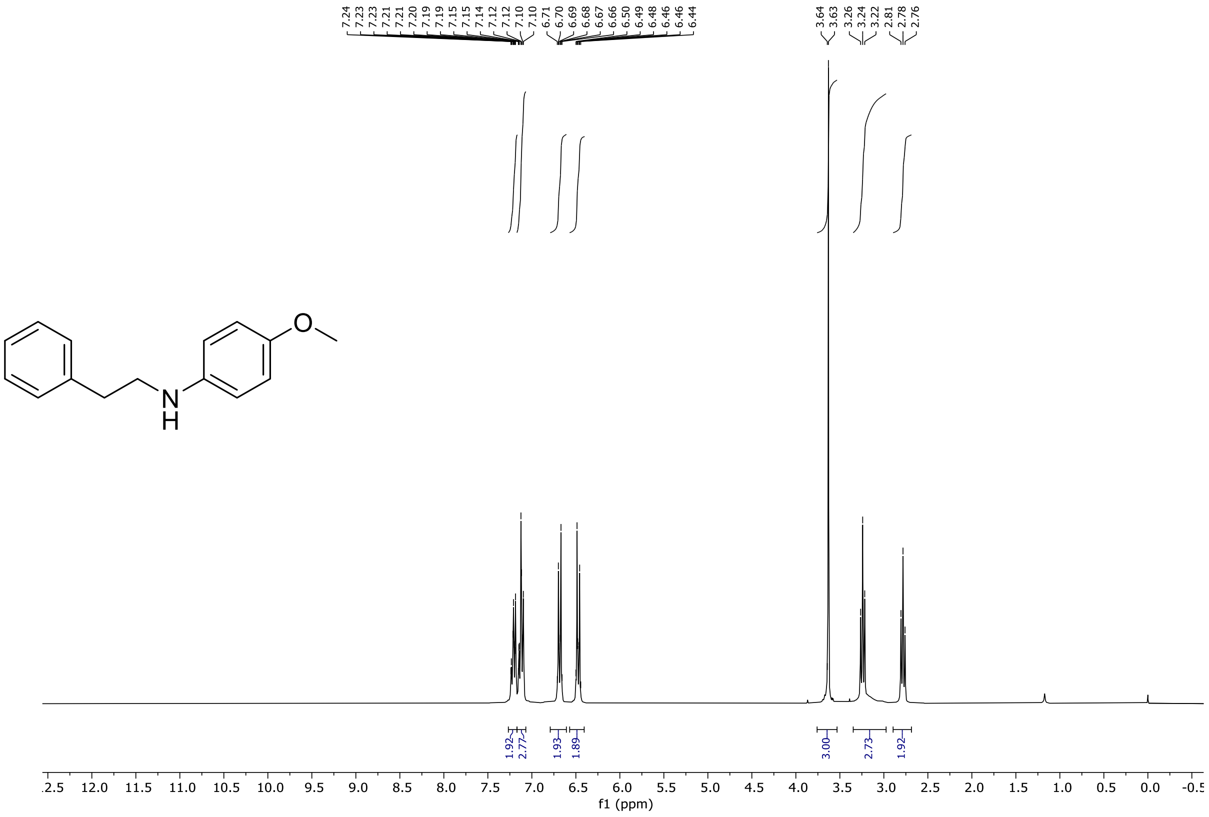

^{1}H NMR (300 MHz, $CDCl_3$) 4-methoxy-*N*-phenethylaniline (**2v**)

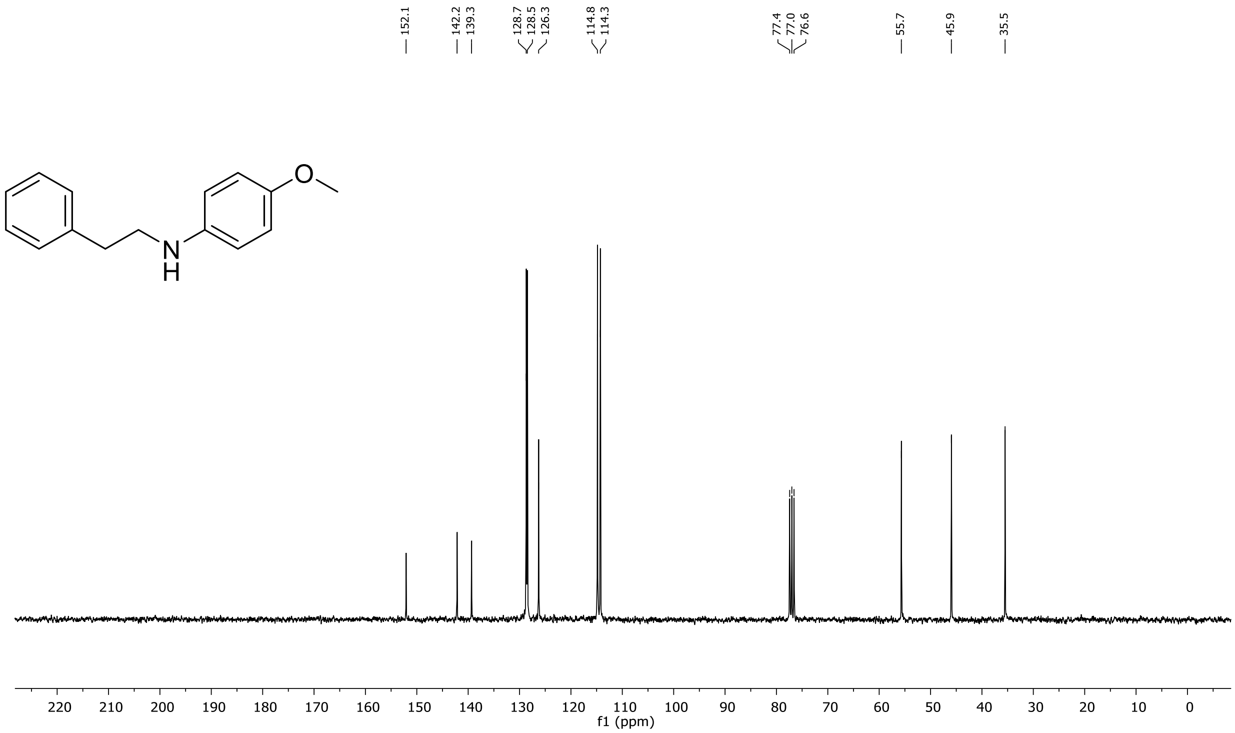

^{13}C {H} NMR (75 MHz, $CDCl_3$) 4-methoxy-*N*-phenethylaniline (**2v**)

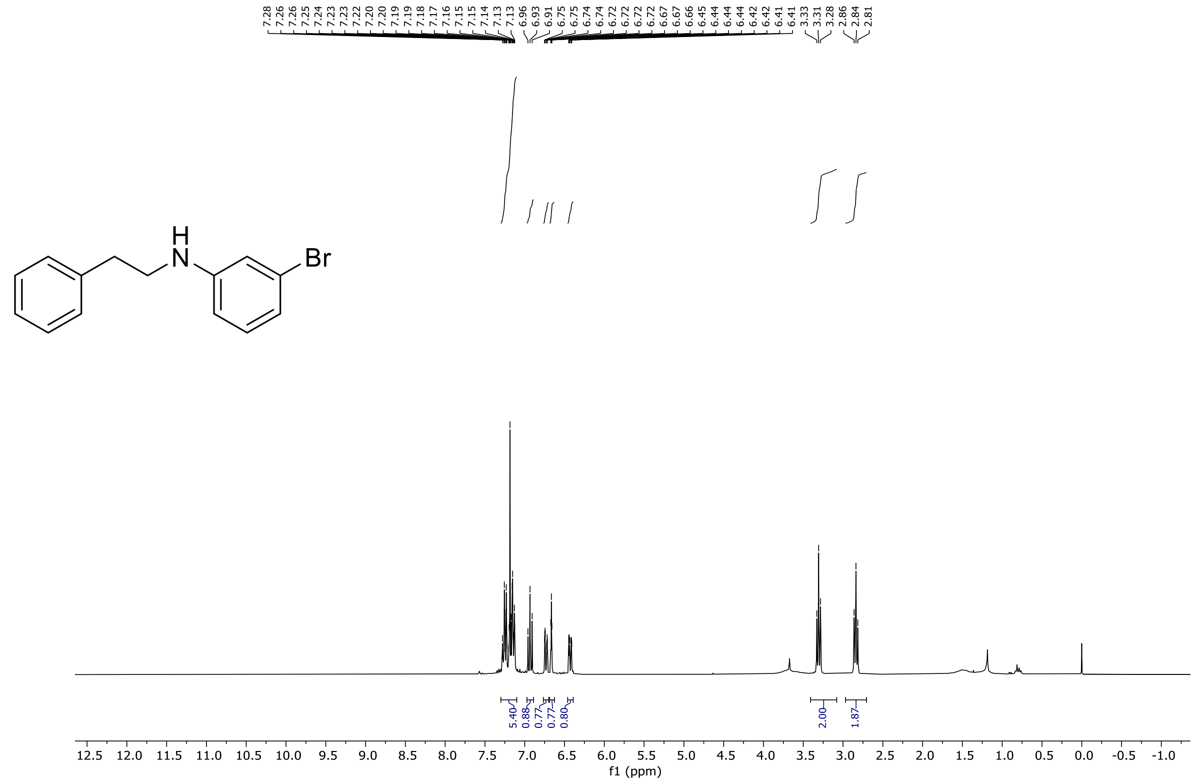

^{1}H NMR (300 MHz, $CDCl_3$) 3-bromo-*N*-phenethylaniline (**2w**)

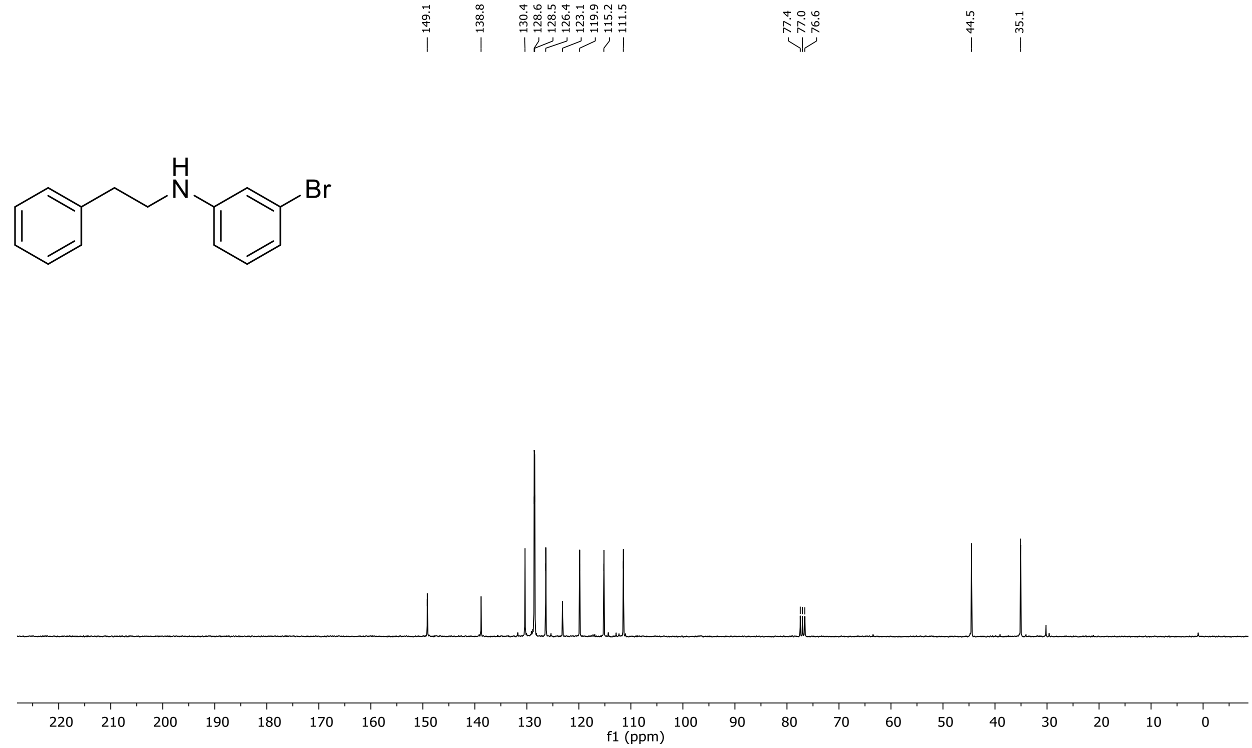

^{13}C {H} NMR (75 MHz, $CDCl_3$) 3-bromo-*N*-phenethylaniline (**2w**)

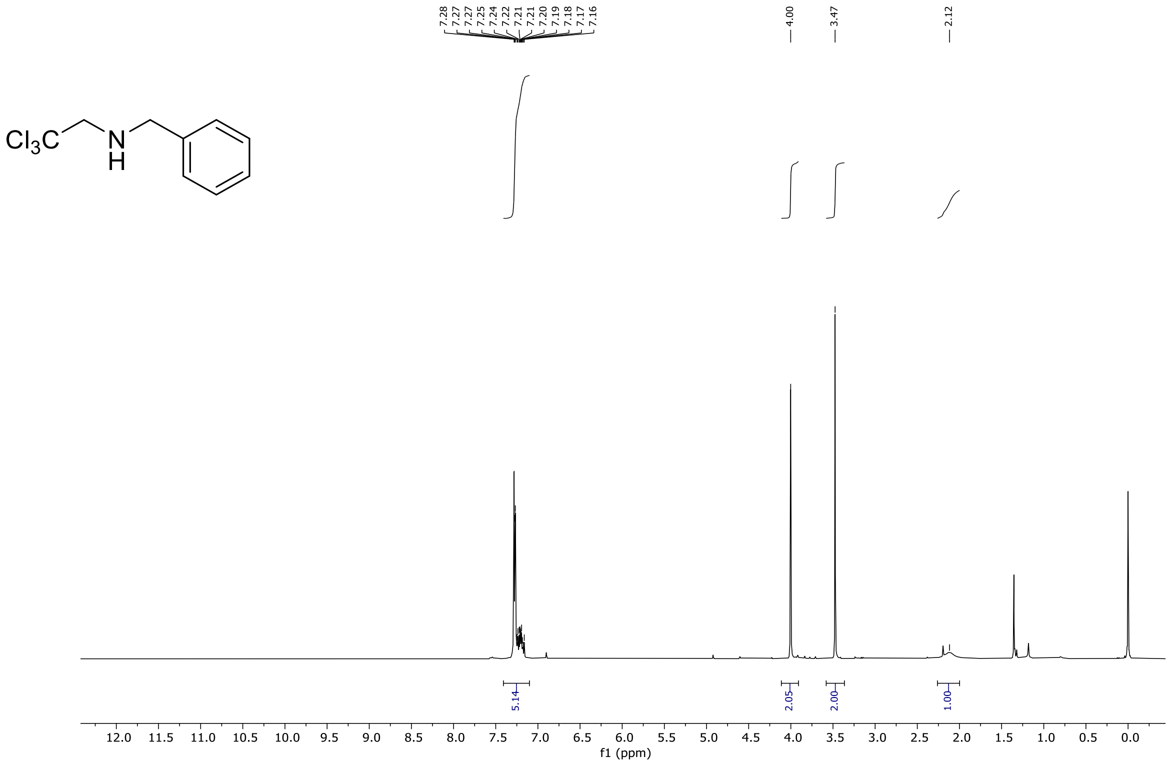

^{1}H NMR (300 MHz, $CDCl_3$) *N*-benzyl-2,2,2-trichloroethan-1-amine (**2x**)

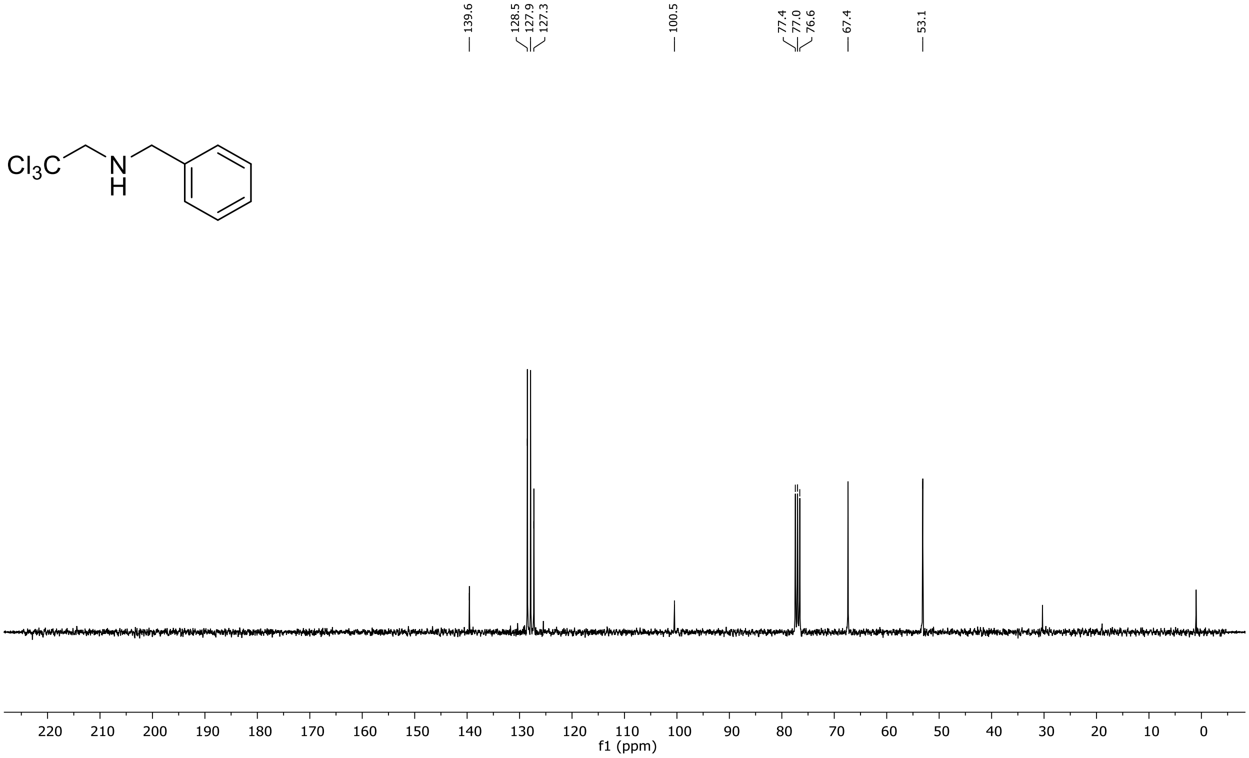

^{13}C {H} NMR (75 MHz, $CDCl_3$) *N*-benzyl-2,2,2-trichloroethan-1-amine (**2x**)

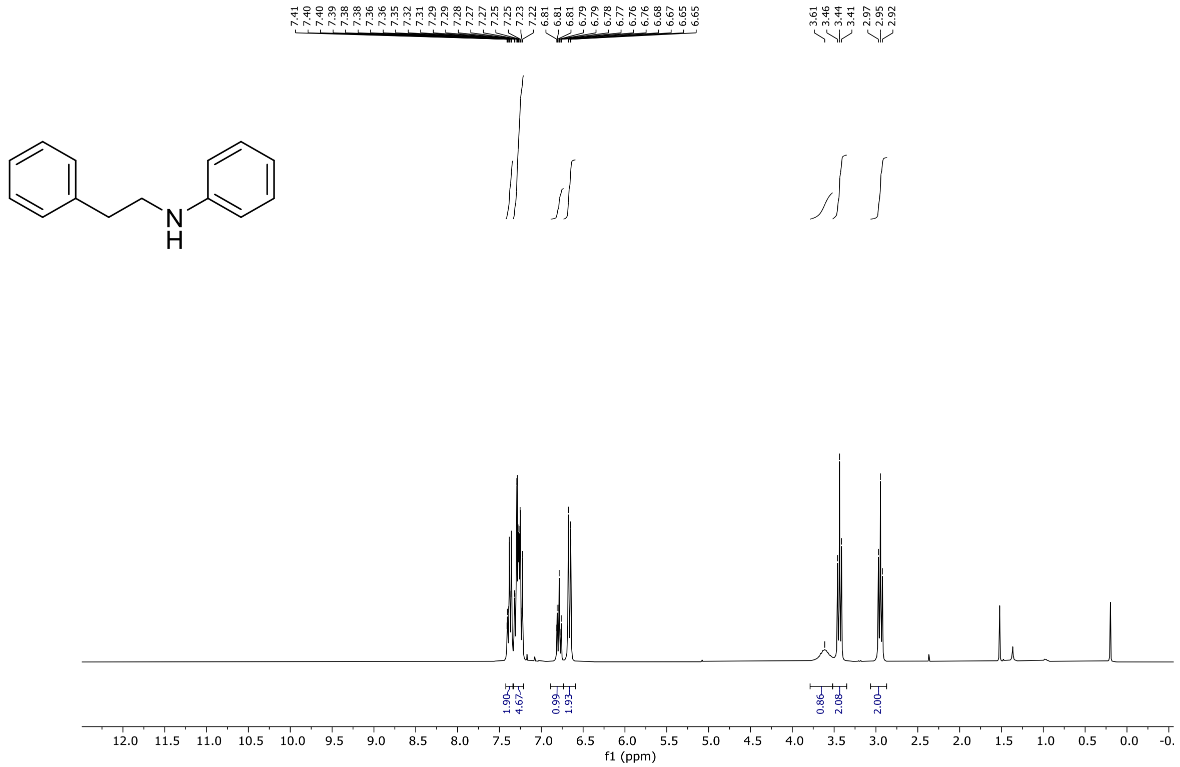

^{1}H NMR (300 MHz, $CDCl_3$) *N*-phenethylaniline (**3z**)

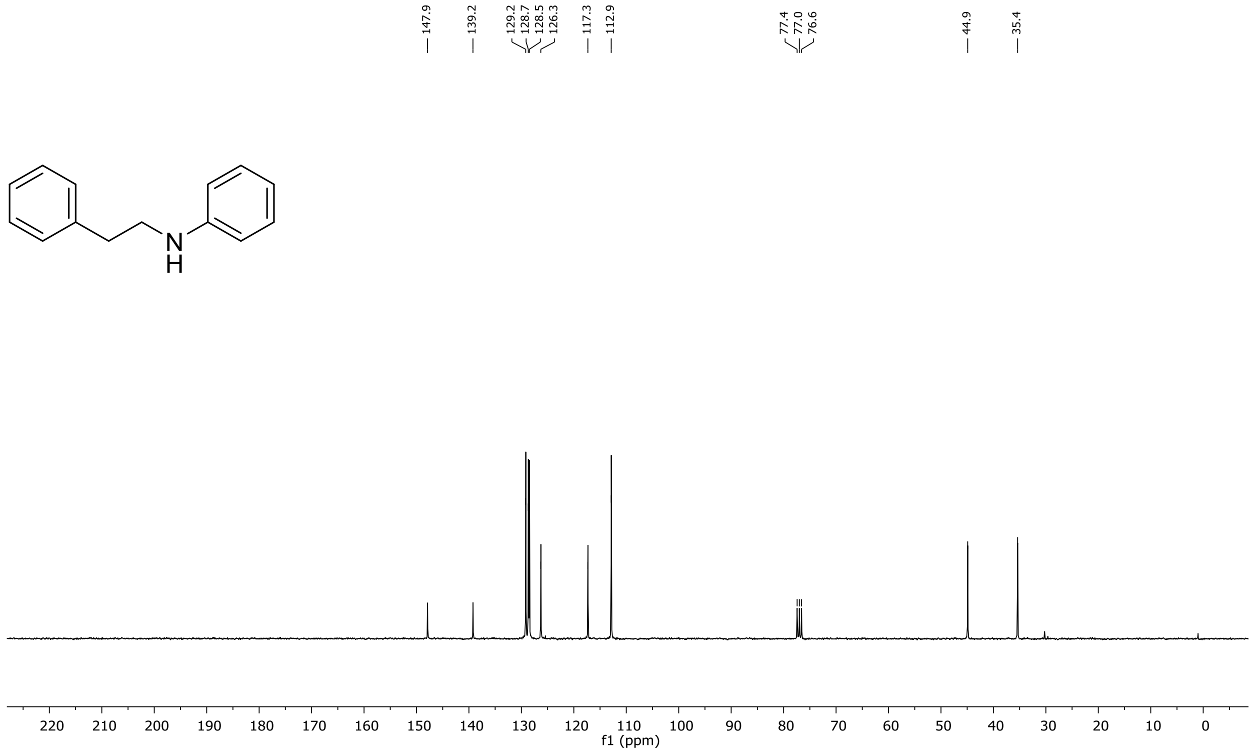

^{13}C {H} NMR (75 MHz, $CDCl_3$) *N*-phenethylaniline (**3z**)

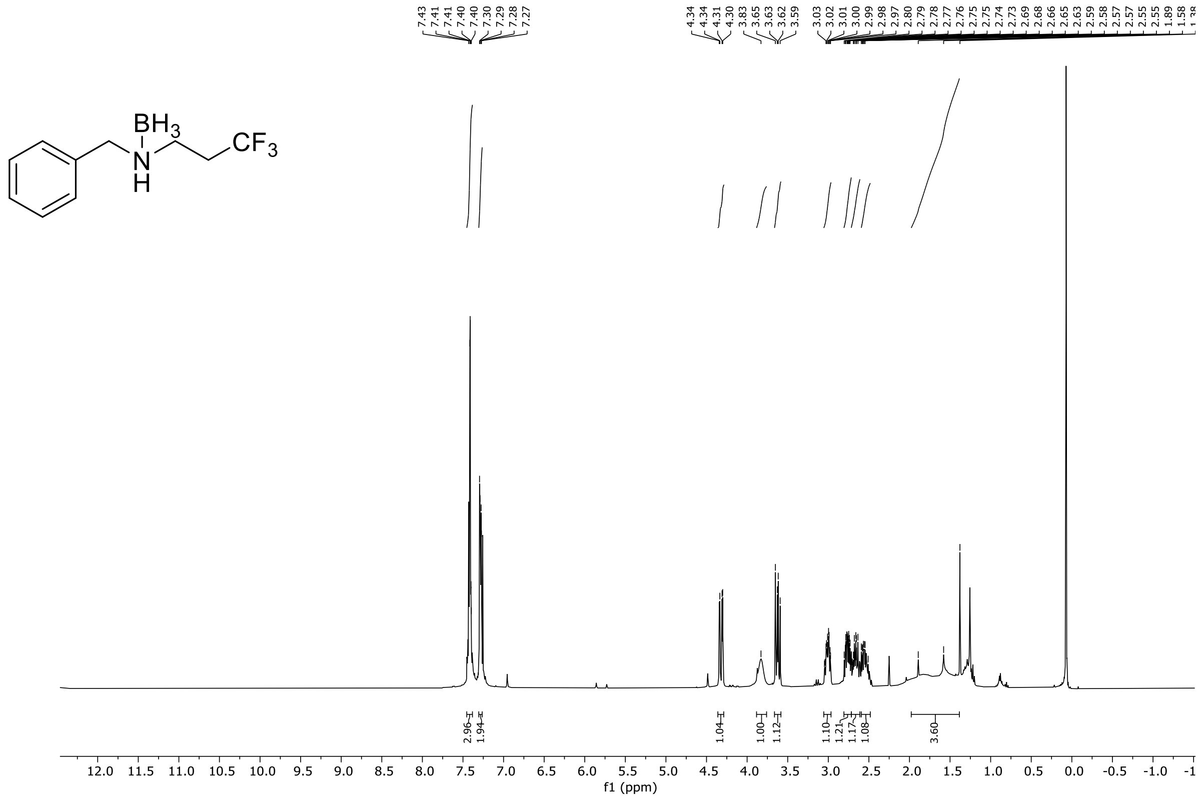

^{1}H NMR (400 MHz, $CDCl_3$) borane-benzenemethanamine, N-(3,3,3-trifluoropropyl) (**3aa**-BH_3)

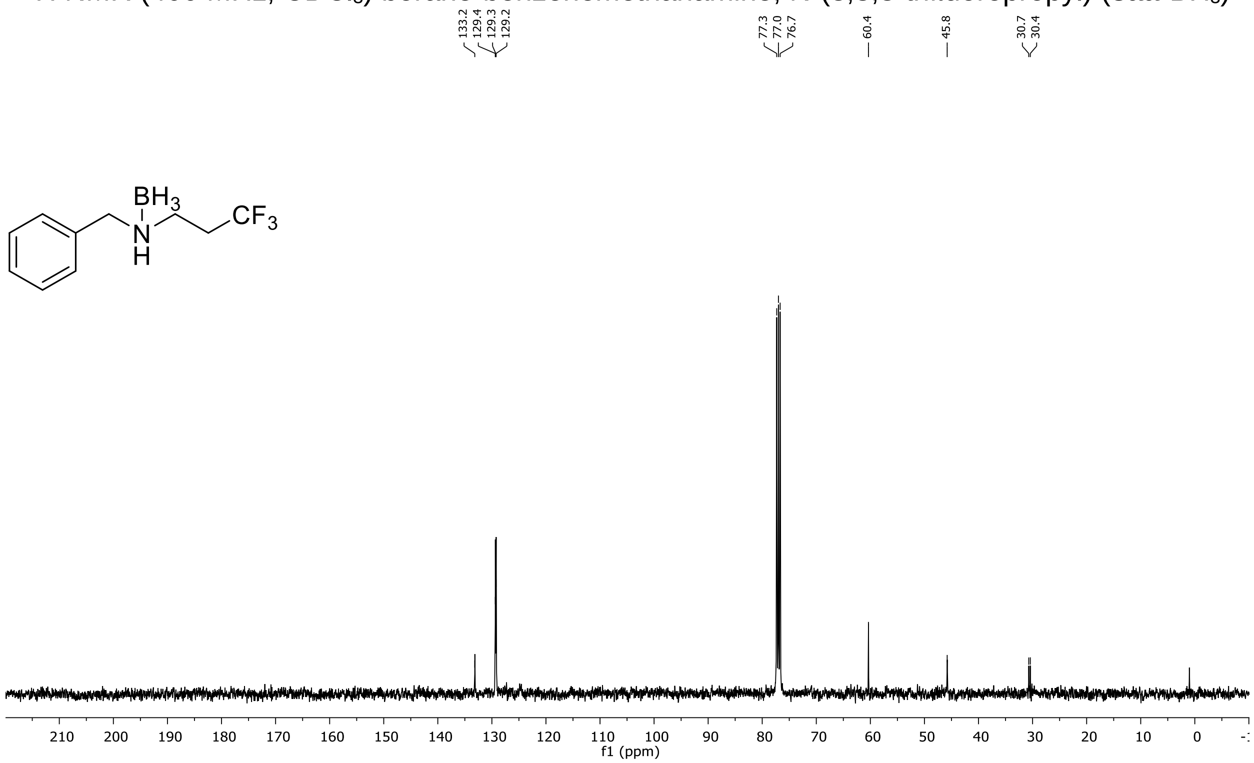

^{13}C {H} NMR (101 MHz, $CDCl_3$) borane-benzenemethanamine, N-(3,3,3-trifluoropropyl) (**3aa**-BH_3)

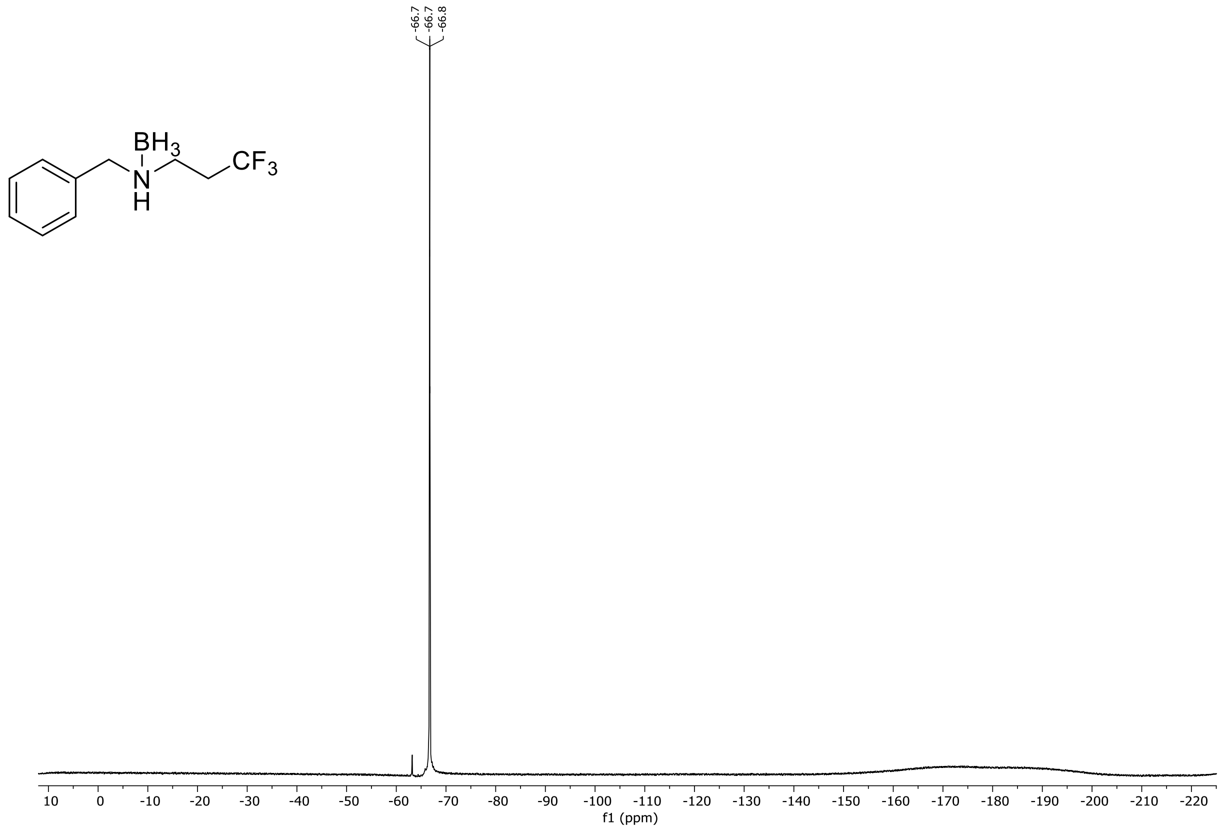

^{19}F NMR (376 MHz, $CDCl_3$) borane-benzenemethanamine, N-(3,3,3-trifluoropropyl) (**3aa**-BH_3)

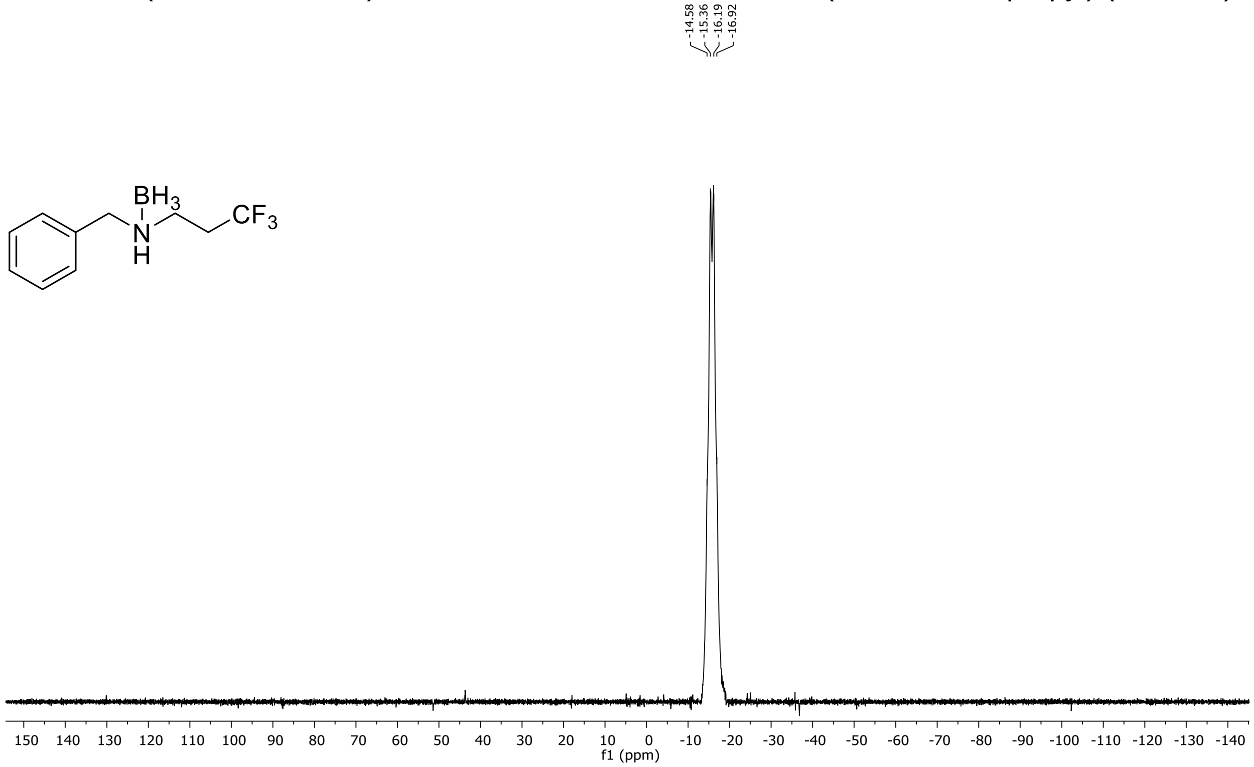

^{11}B NMR (96 MHz, $CDCl_3$) borane-benzenemethanamine, *N*-(3,3,3-trifluoropropyl) (**3aa**-BH_3)

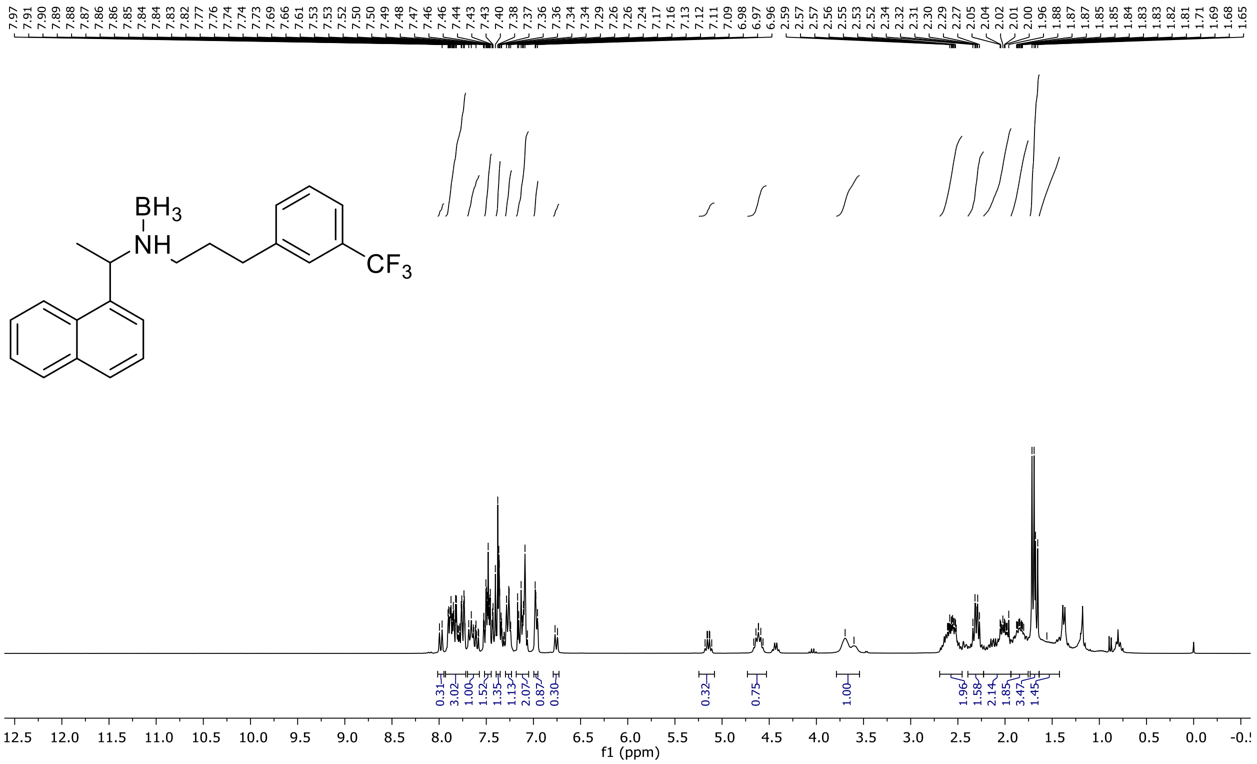

^{1}H NMR (400 MHz, $CDCl_3$) *N*-(1-(naphthalen-1-yl)ethyl)-3-(3-(trifluoromethyl)phenyl)propan-1-amine borane (**4a-BH_3** - *mixture*)

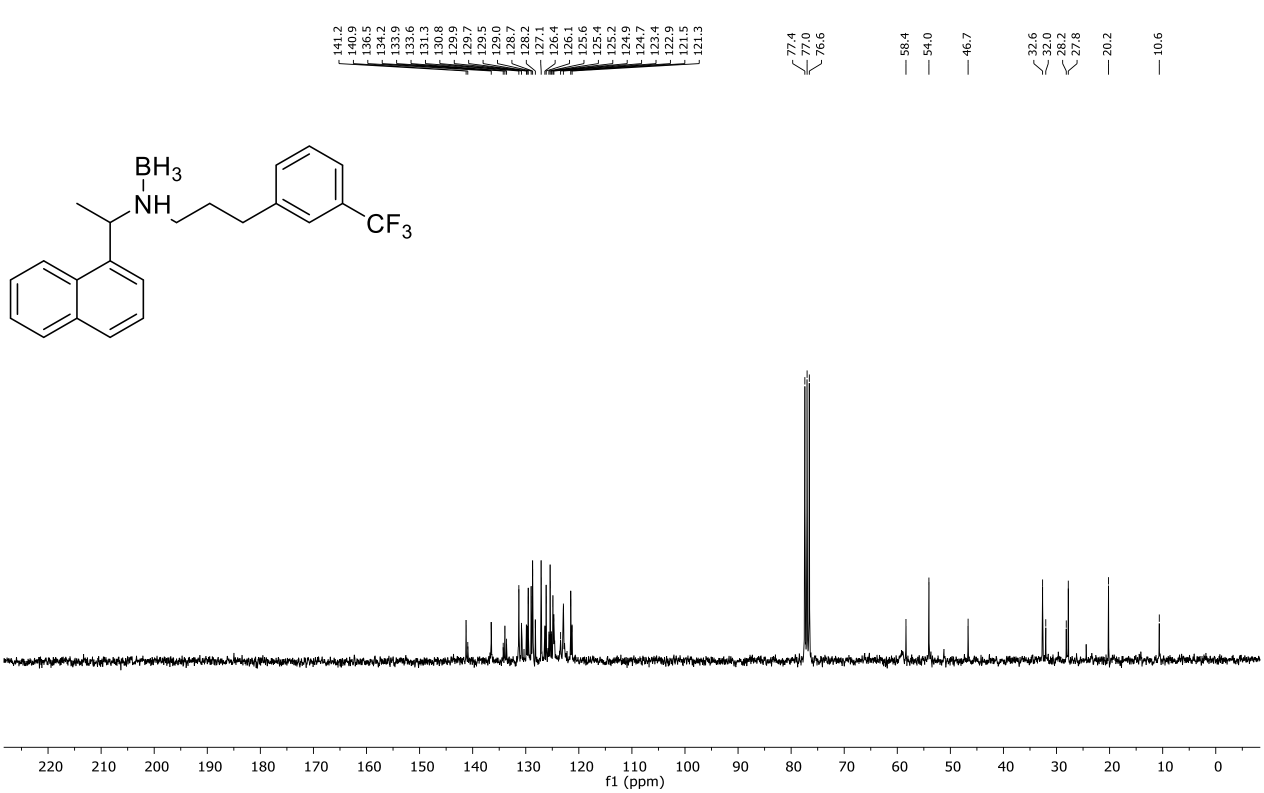

^{13}C {H} NMR (101 MHz, $CDCl_3$) *N*-(1-(naphthalen-1-yl)ethyl)-3-(3-(trifluoromethyl)phenyl)propan-1-amine borane (**4a-BH_3** - *mixture*)

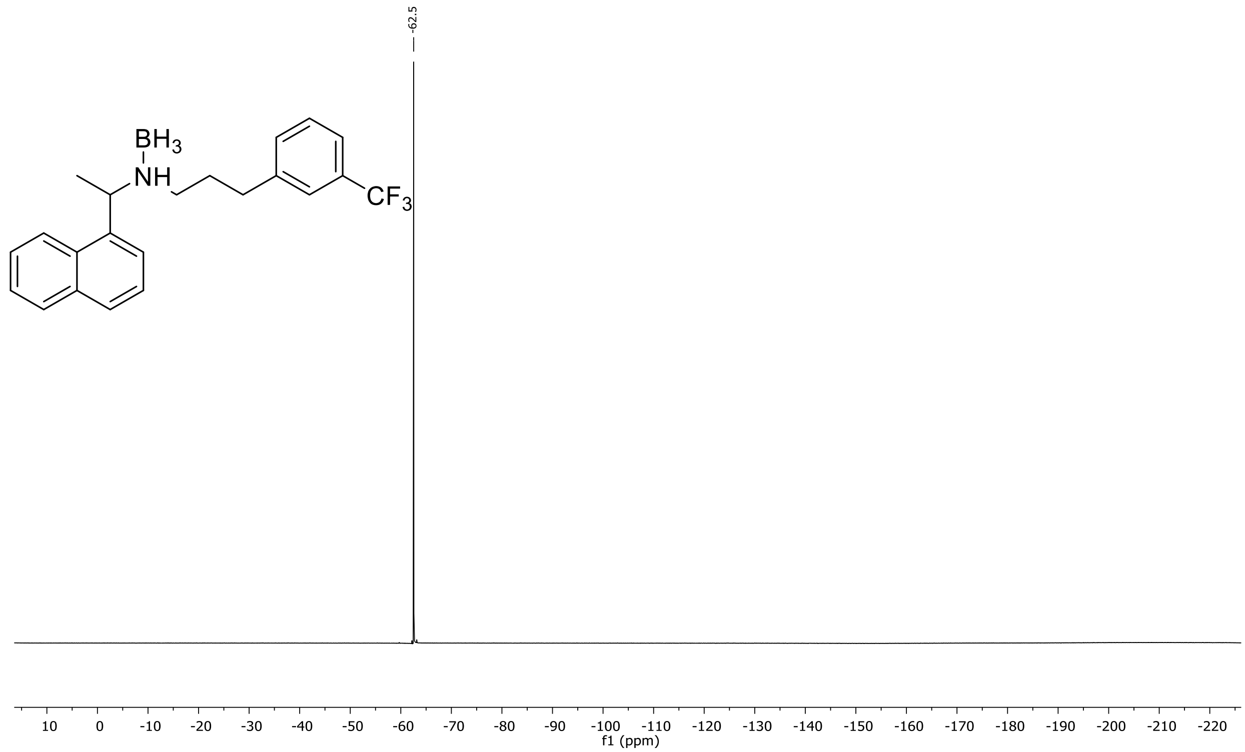

^{19}F NMR (376 MHz, $CDCl_3$) *N*-(1-(naphthalen-1-yl)ethyl)-3-(3-(trifluoromethyl)phenyl)propan-1-amine borane (**4a-BH_3 -** *mixture*)

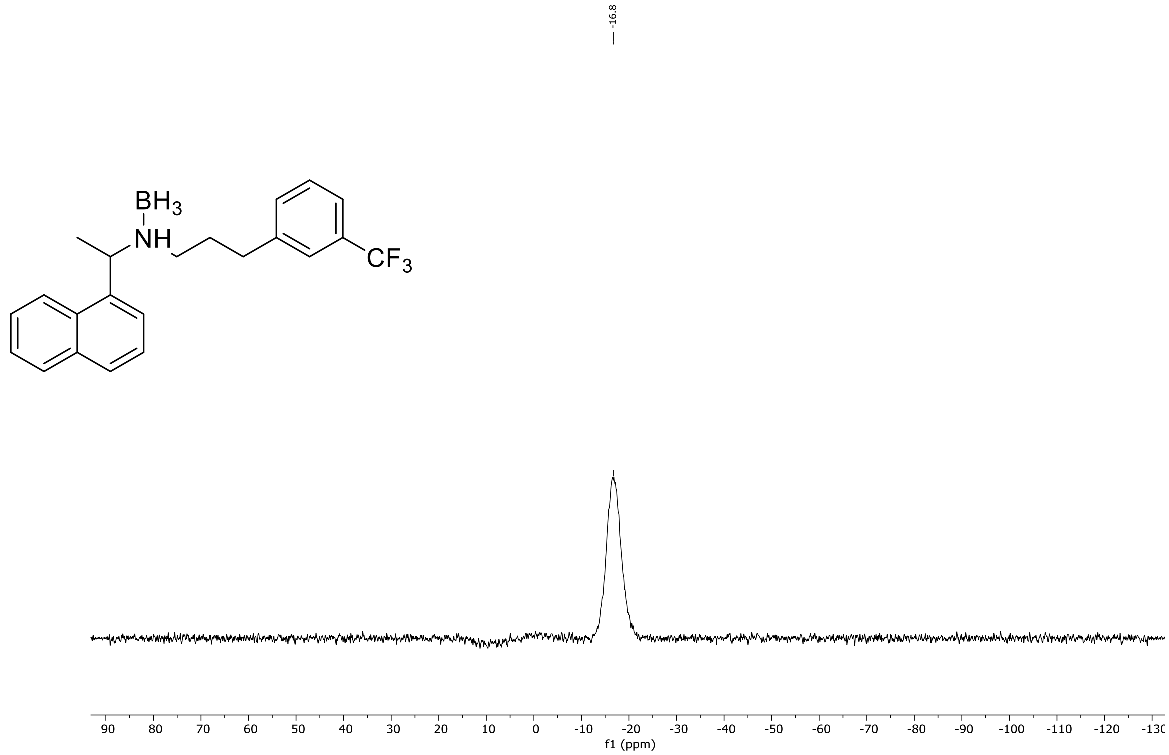

^{11}B NMR (96 MHz, $CDCl_3$) *N*-(1-(naphthalen-1-yl)ethyl)-3-(3-(trifluoromethyl)phenyl)propan-1-amine borane (**4a-BH_3 -** *mixture*)

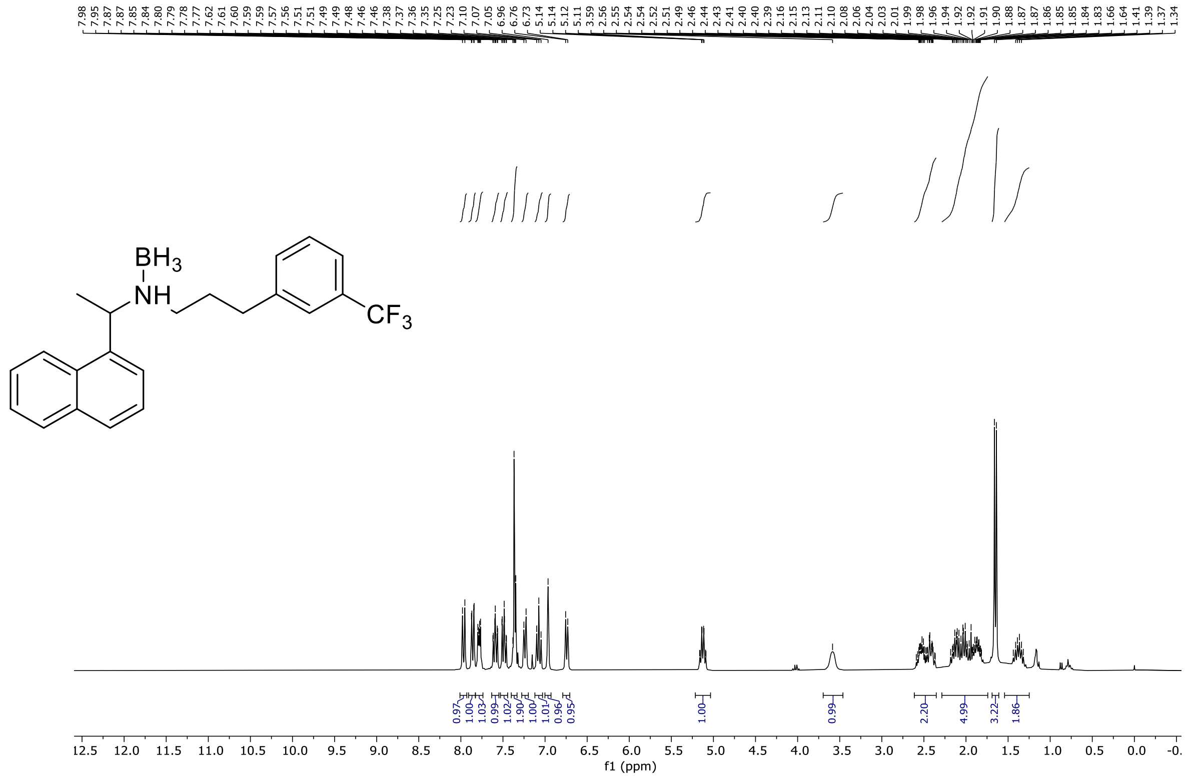

^{1}H NMR (400 MHz, $CDCl_3$) *N*-(1-(naphthalen-1-yl)ethyl)-3-(3-(trifluoromethyl)phenyl)propan-1-amine borane (**4a-BH$_3$ –** *component 1*)

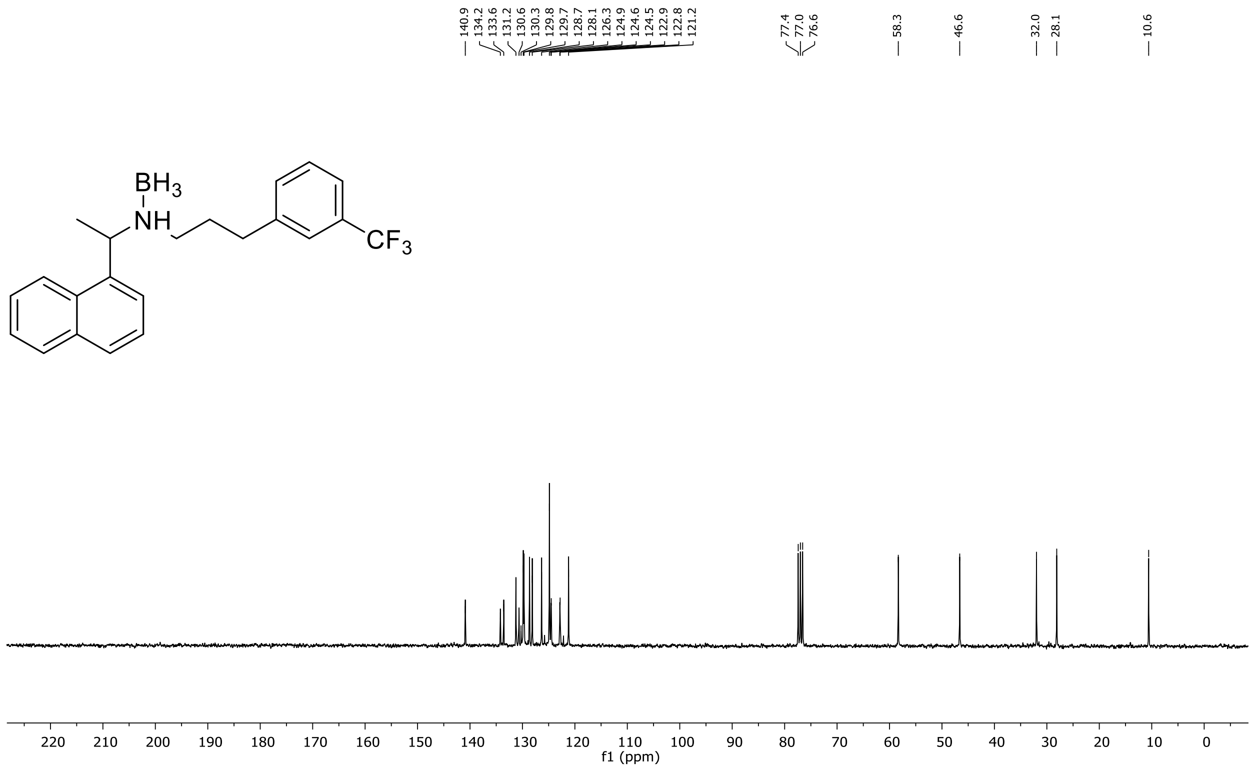

^{13}C {H} NMR (101 MHz, $CDCl_3$) *N*-(1-(naphthalen-1-yl)ethyl)-3-(3-(trifluoromethyl)phenyl)propan-1-amine borane (**4a-BH$_3$ –** *component 1*)

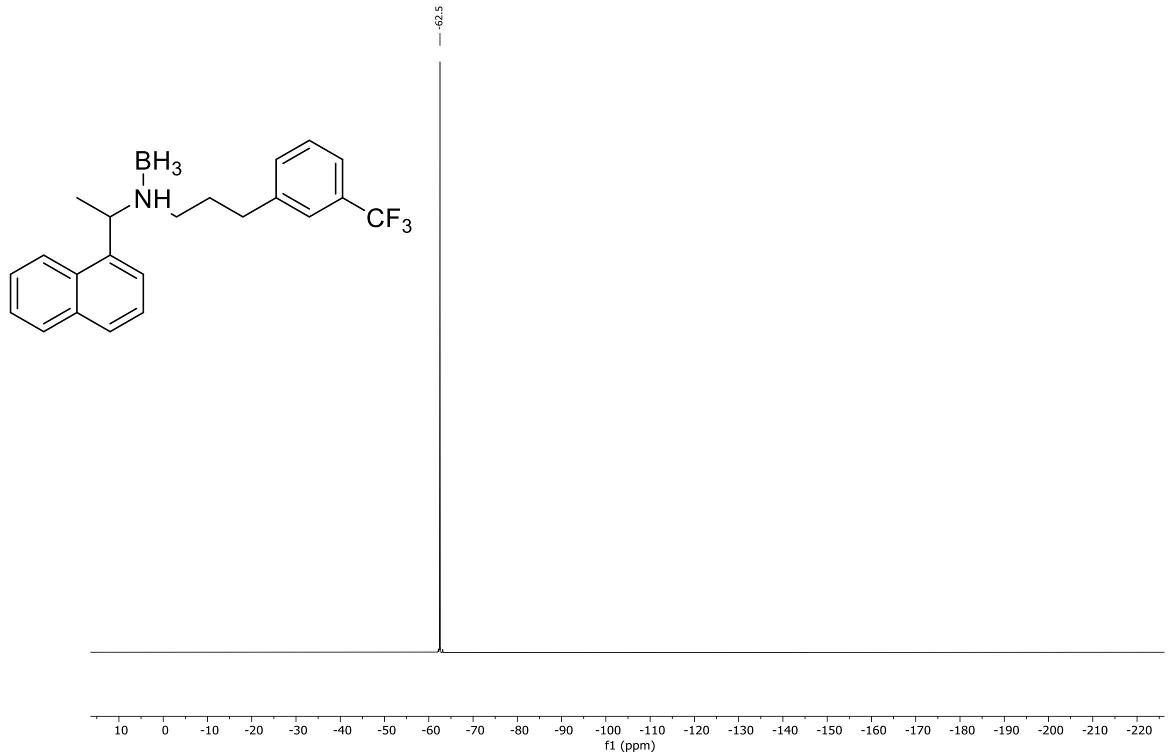

^{19}F NMR (376 MHz, $CDCl_3$) *N*-(1-(naphthalen-1-yl)ethyl)-3-(3-(trifluoromethyl)phenyl)propan-1-amine borane (**4a-BH$_3$ –** *component 1*)

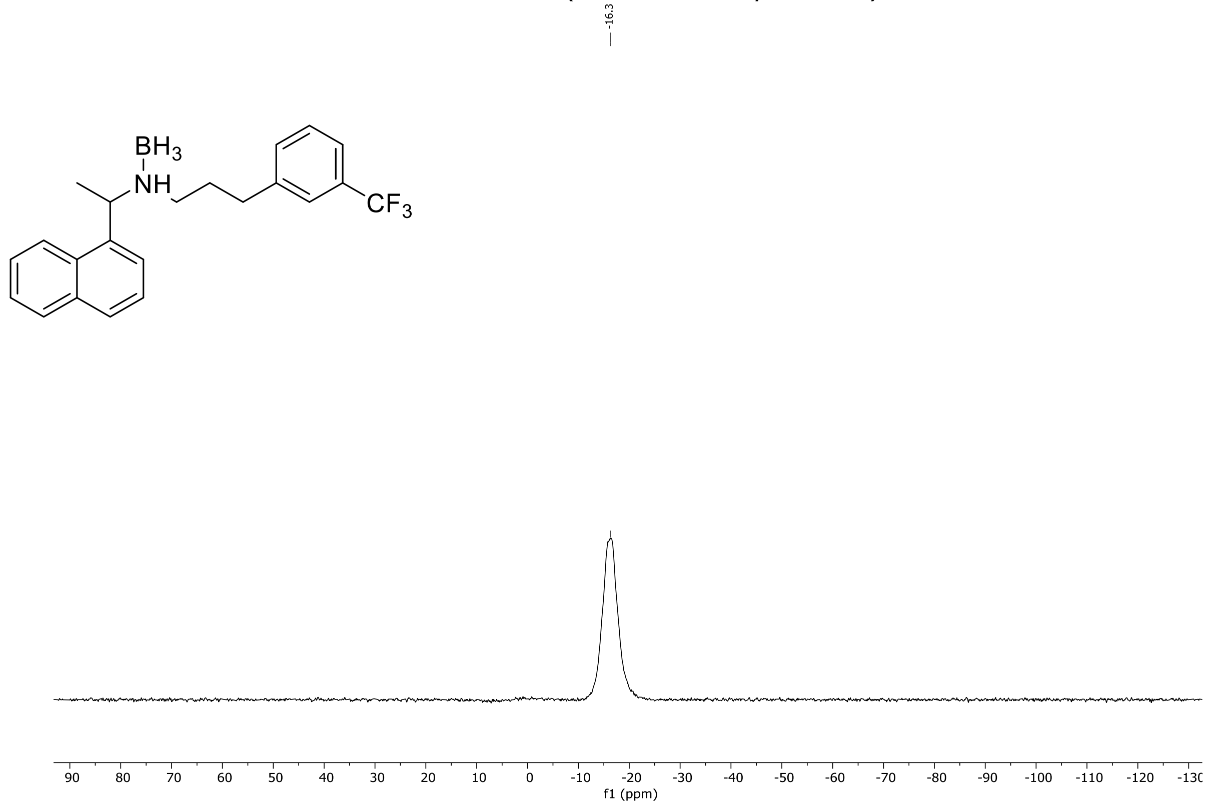

^{11}B NMR (96 MHz, $CDCl_3$) *N*-(1-(naphthalen-1-yl)ethyl)-3-(3-(trifluoromethyl)phenyl)propan-1-amine borane (**4a-BH$_3$ –** *component 1*)

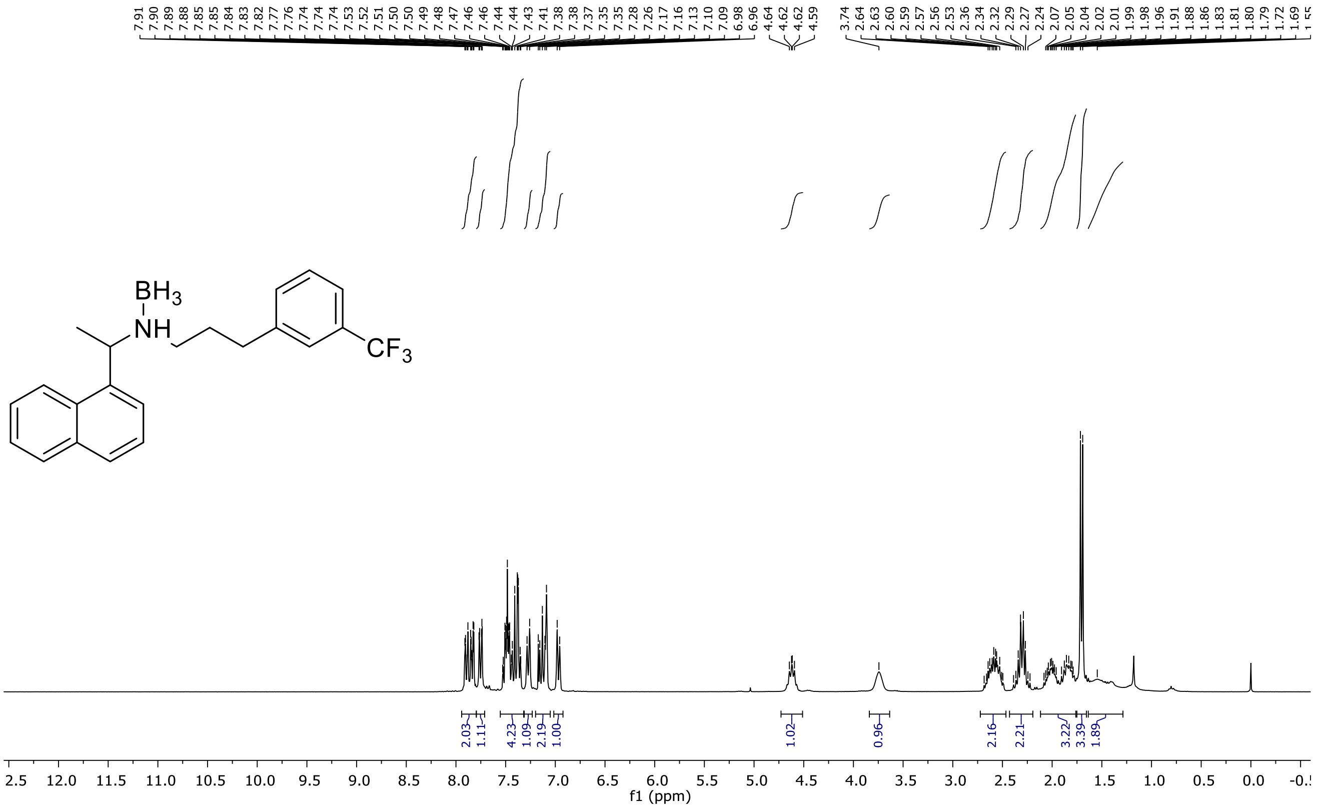

^{1}H NMR (400 MHz, $CDCl_3$) *N*-(1-(naphthalen-1-yl)ethyl)-3-(3-(trifluoromethyl)phenyl)propan-1-amine borane (**4a-BH₃ –** *component 2*)

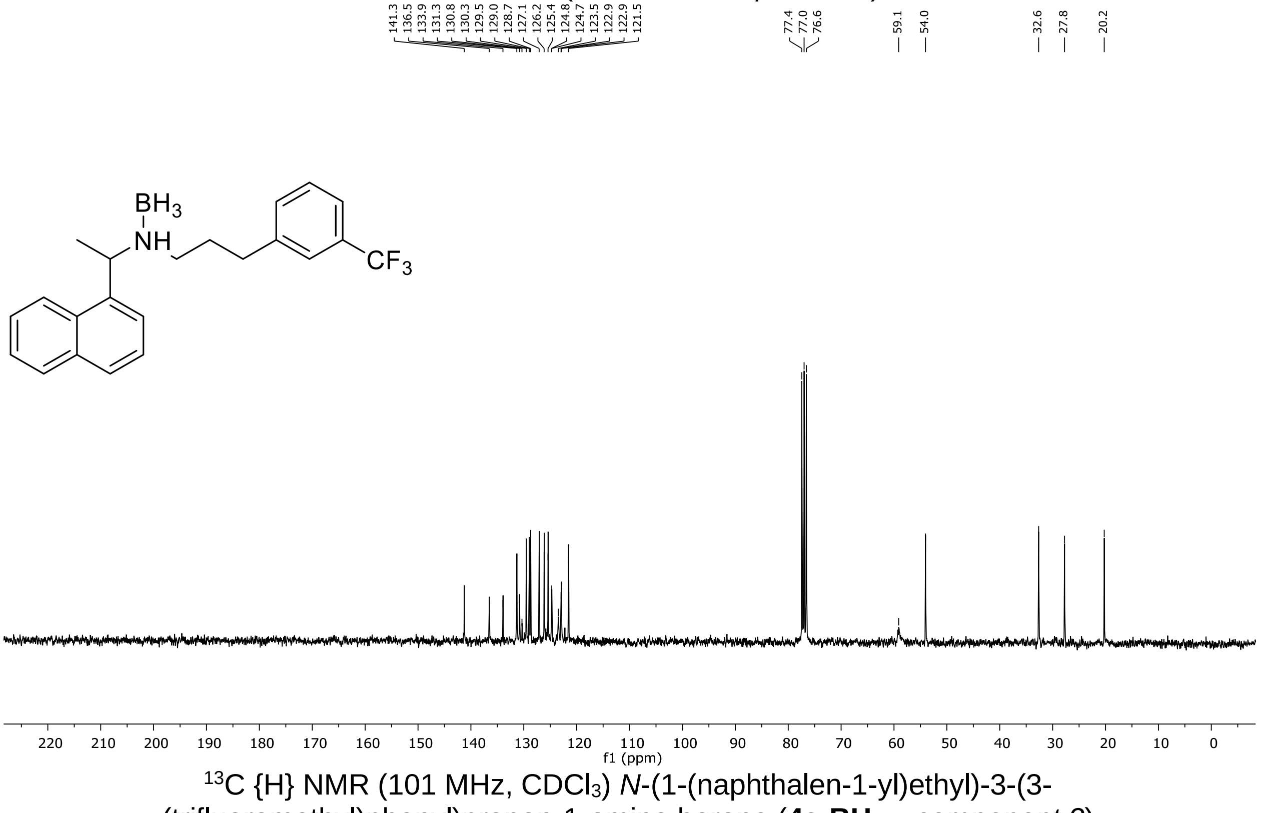

^{13}C {H} NMR (101 MHz, $CDCl_3$) *N*-(1-(naphthalen-1-yl)ethyl)-3-(3-(trifluoromethyl)phenyl)propan-1-amine borane (**4a-BH₃ –** *component 2*)

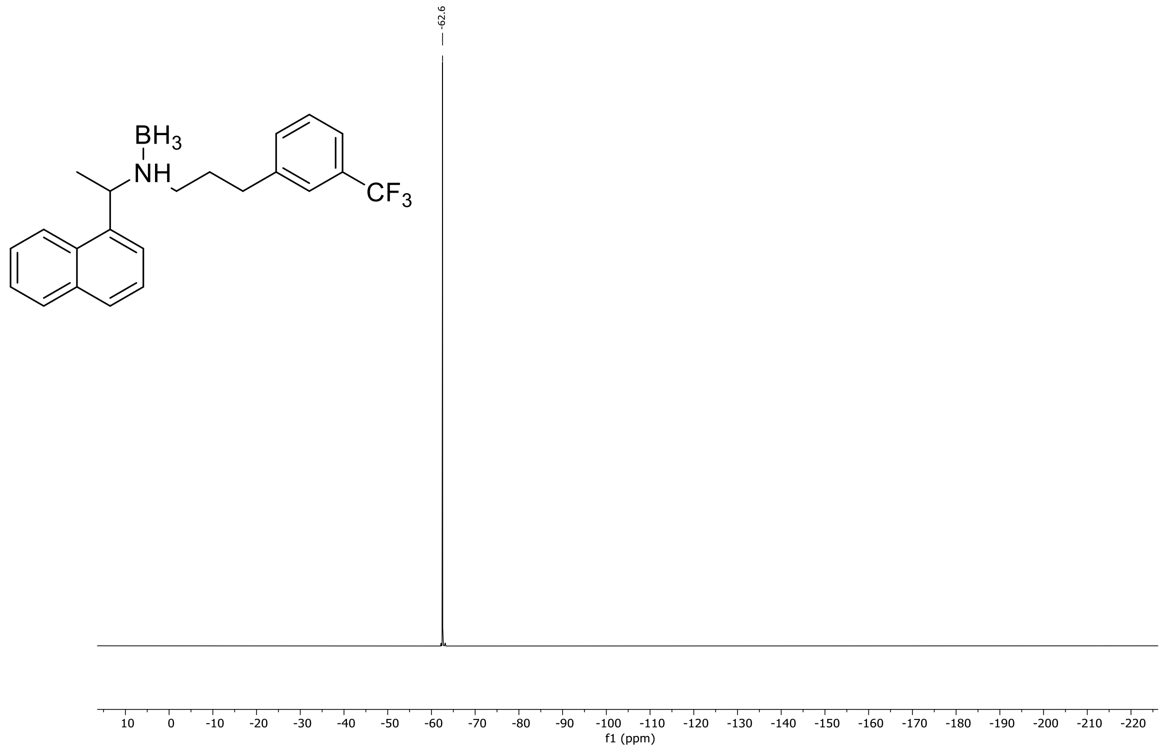

^{19}F NMR (376 MHz, $CDCl_3$) *N*-(1-(naphthalen-1-yl)ethyl)-3-(3-(trifluoromethyl)phenyl)propan-1-amine borane (**4a-BH_3 –** *component 2*)

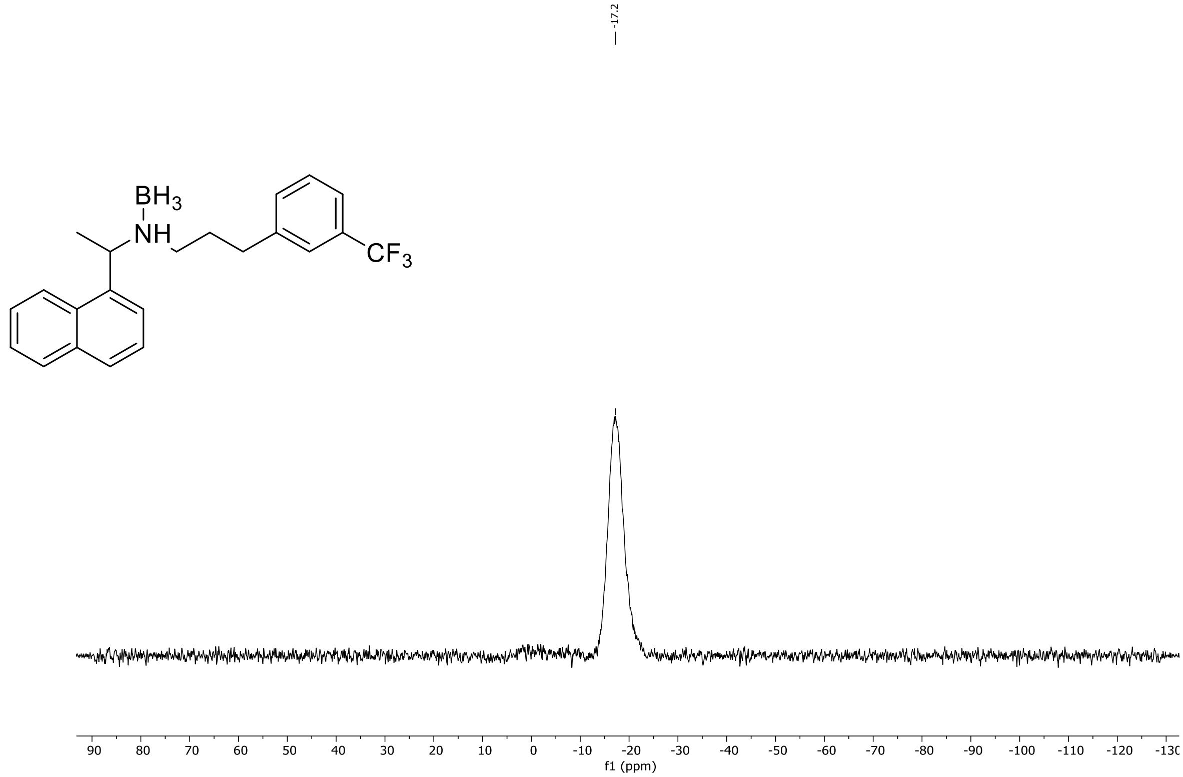

^{11}B NMR (96 MHz, $CDCl_3$) *N*-(1-(naphthalen-1-yl)ethyl)-3-(3-(trifluoromethyl)phenyl)propan-1-amine borane (**4a-BH_3 –** *component 2*)

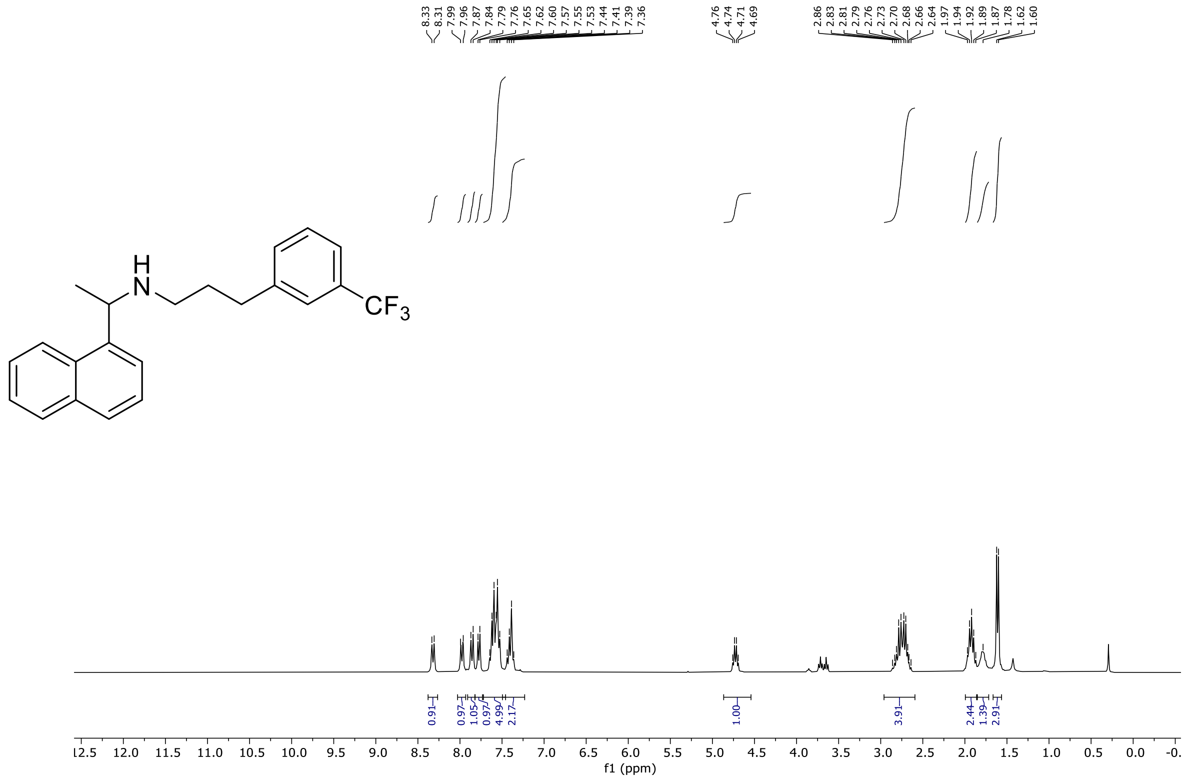

^{1}H NMR (400 MHz, $CDCl_3$) *N*-(1-(naphthalen-1-yl)ethyl)-3-(3-(trifluoromethyl)phenyl)propan-1-amine (racemic Cinacalcet) (**4a**)

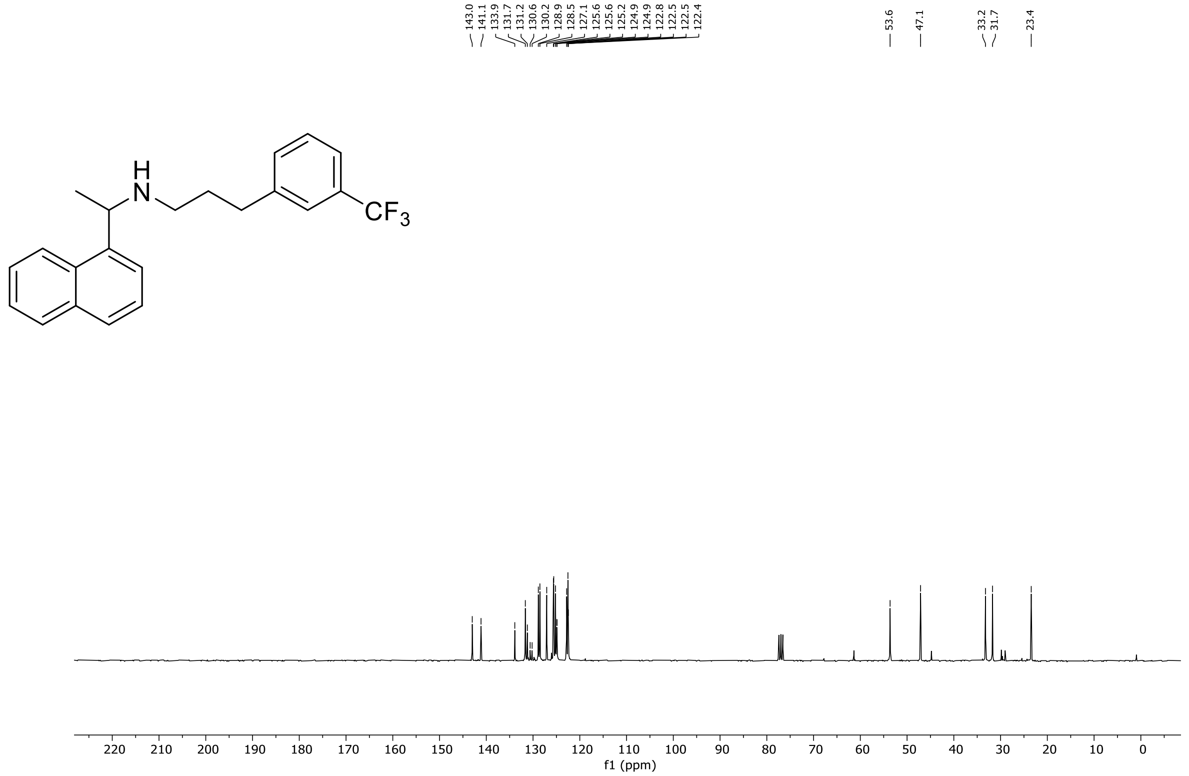

^{13}C {H} NMR (101 MHz, $CDCl_3$) *N*-(1-(naphthalen-1-yl)ethyl)-3-(3-(trifluoromethyl)phenyl)propan-1-amine (racemic Cinacalcet) (**4a**)

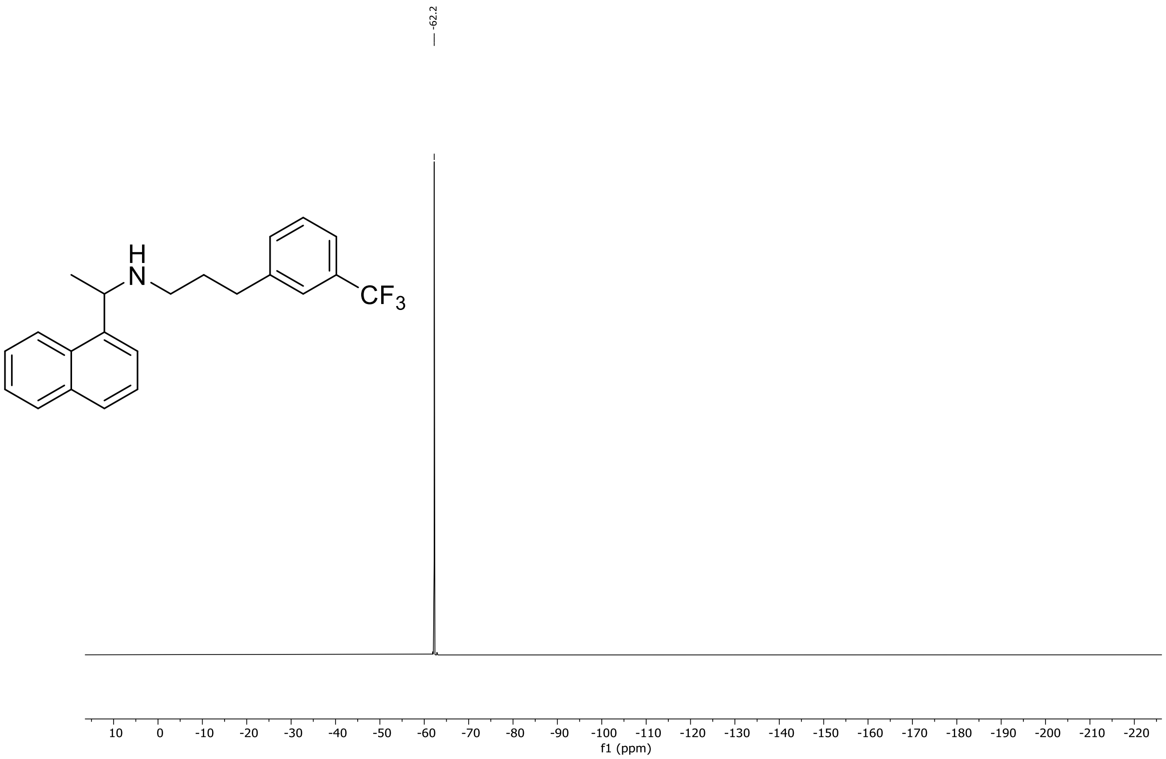

^{19}F NMR (376 MHz, $CDCl_3$) *N*-(1-(naphthalen-1-yl)ethyl)-3-(3-(trifluoromethyl)phenyl)propan-1-amine (racemic Cinacalcet) (**4a**)
